# Supplementary figures and images for: Time series analyses based on the joint lagged effect analysis of pollution and meteorological factors of hemorrhagic fever with renal syndrome and the construction of prediction model
Source: PLoS Negl Trop Dis. 2023 Jul 24;17(7):e0010806. doi: 10.1371/journal.pntd.0010806 (PMC10399869; doi:10.1371/journal.pntd.0010806)

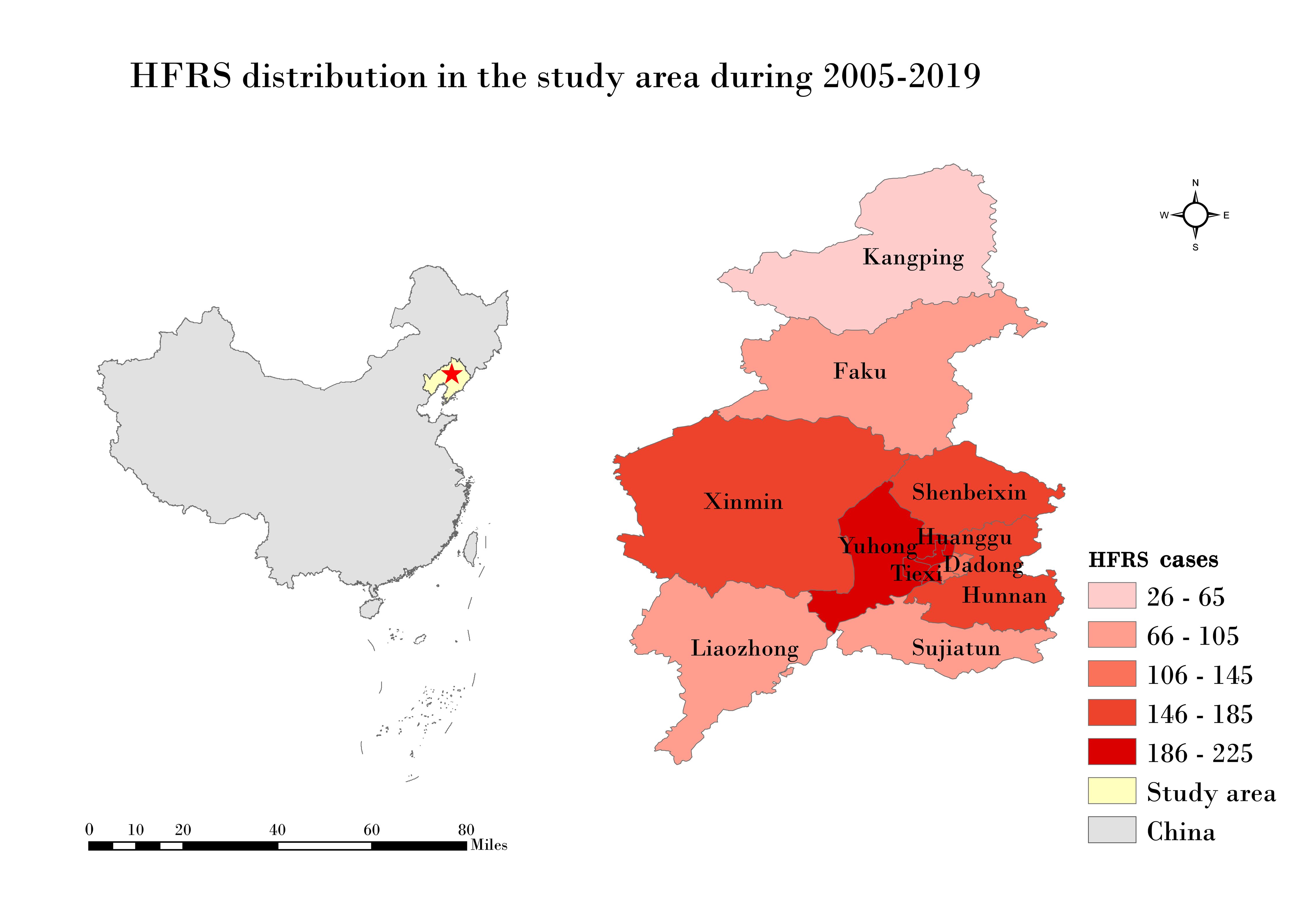

Supplement: S1 Fig — The map was created by ArcGIS 10.3 (Environmental Systems Research Institute; Redlands, CA, USA). The base map was acquired from the data center for geographic sciences and natural sources research, CAS (http://www.resdc.cn/data.aspx?DATAID=201). (TIF) [file pntd.0010806.s004.tif]

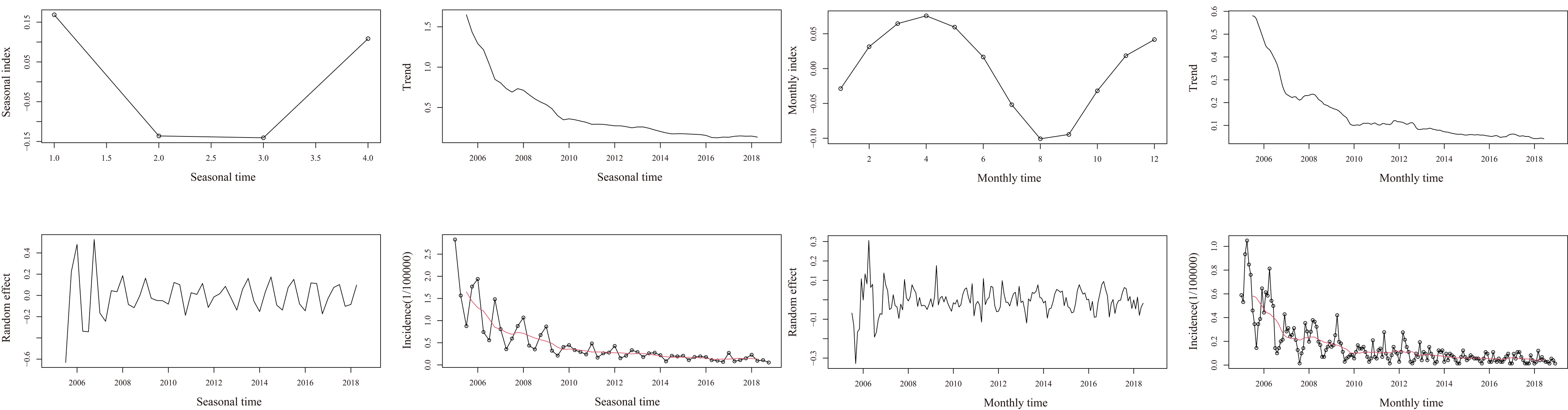

Supplement: S2 Fig — (TIF) [file pntd.0010806.s005.tif]

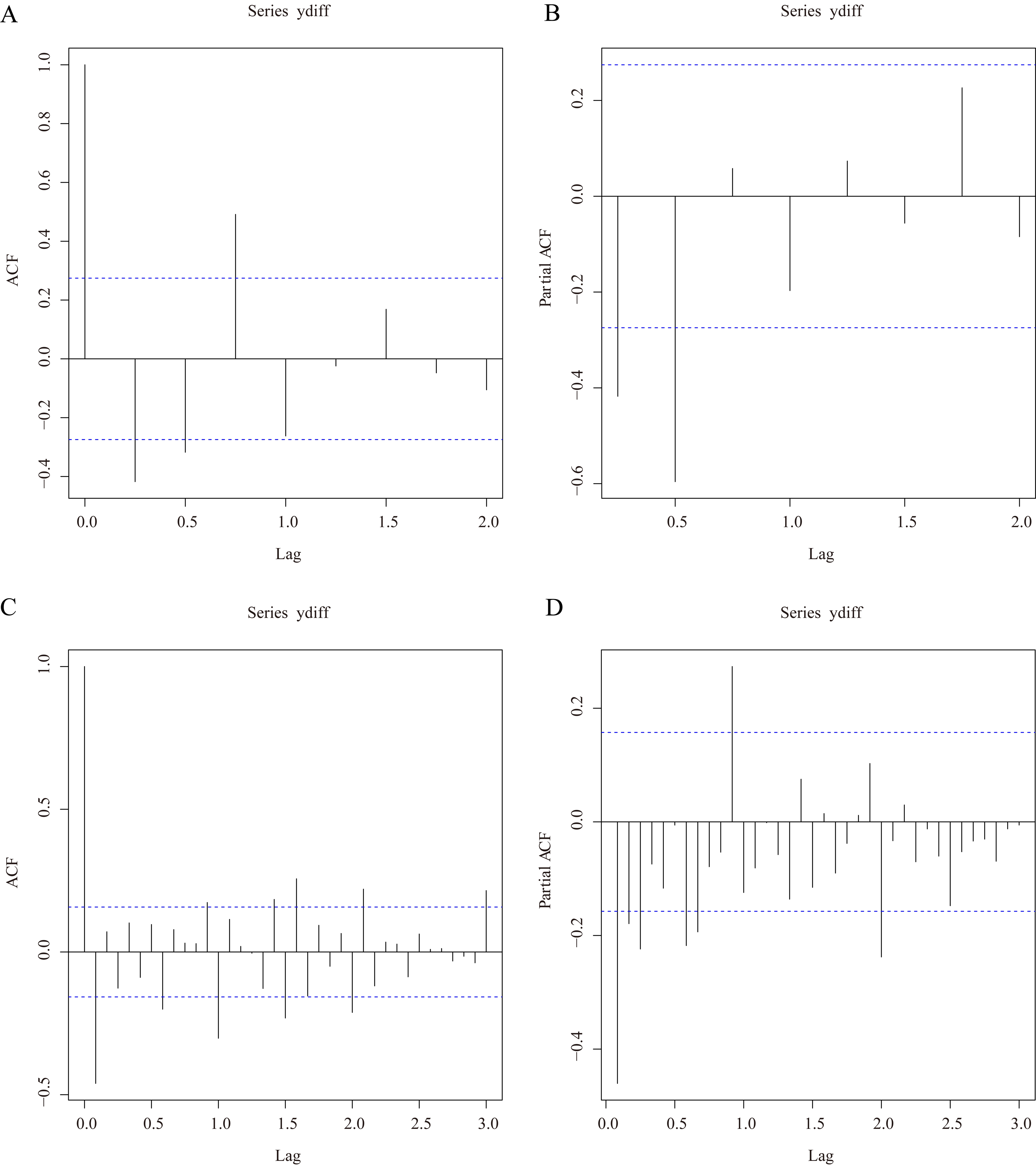

Supplement: S3 Fig — (TIF) [file pntd.0010806.s006.tif]

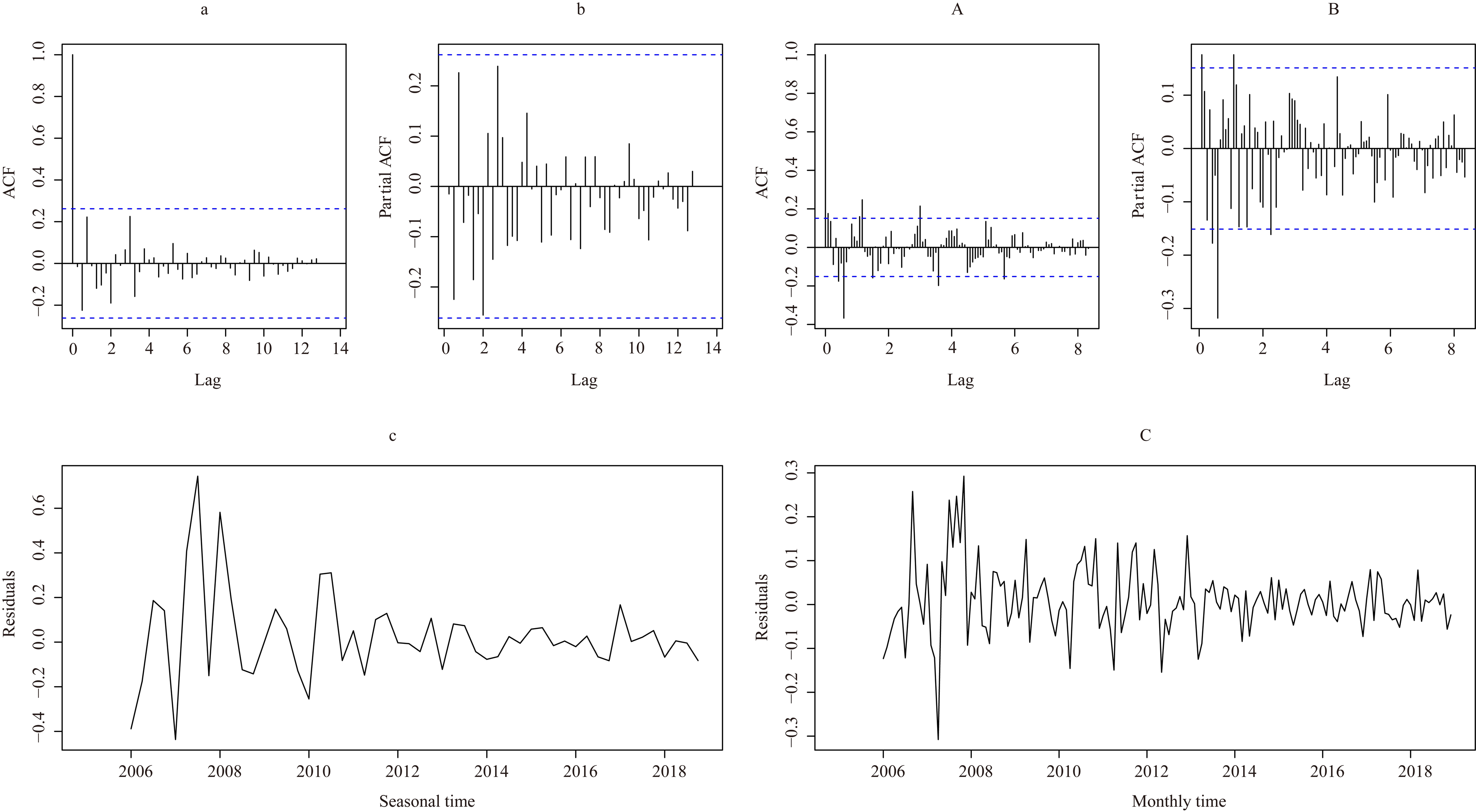

Supplement: S4 Fig — (a)Autocorrelation function (ACF) plot for the seasonal Holt-Winters residual series; (A)Autocorrelation function (ACF) plot for the monthly Holt-Winters residual series; (b) Partial autocorrelation function (PACF) plot for the seasonal Holt-Winters residual series; (B) Partial autocorrelation function (PACF) plot for the monthly Holt-Winters residual series; (c) Standardized residual seasonal Holt-Winters series; (C) Standardized residual monthly Holt-Winters series. These manifested its adequacy and suitability of this data-driven hybrid model for the data. (TIF) [file pntd.0010806.s007.tif]

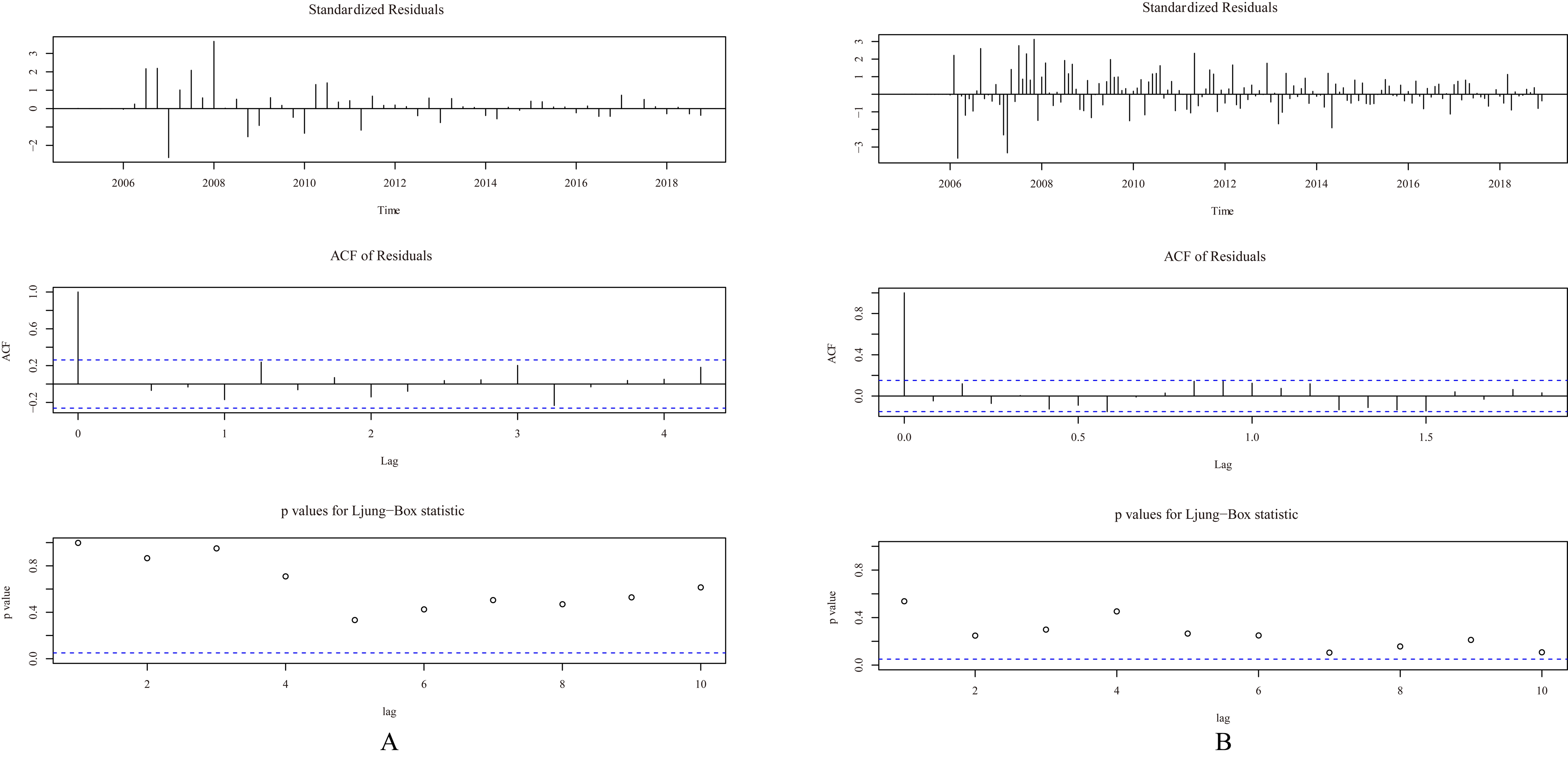

Supplement: S5 Fig — (TIF) [file pntd.0010806.s008.tif]

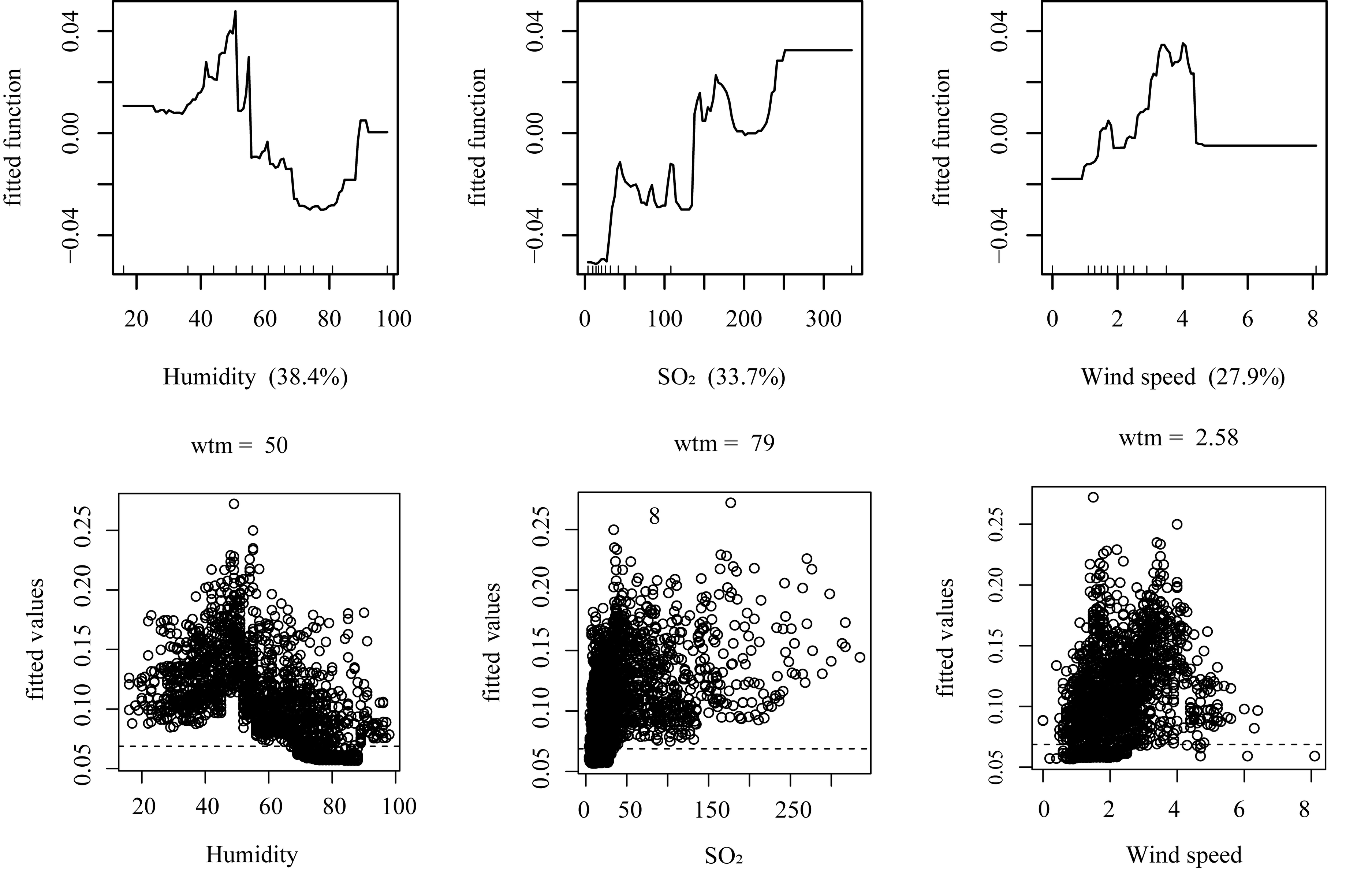

Supplement: S6 Fig — (TIF) [file pntd.0010806.s009.tif]

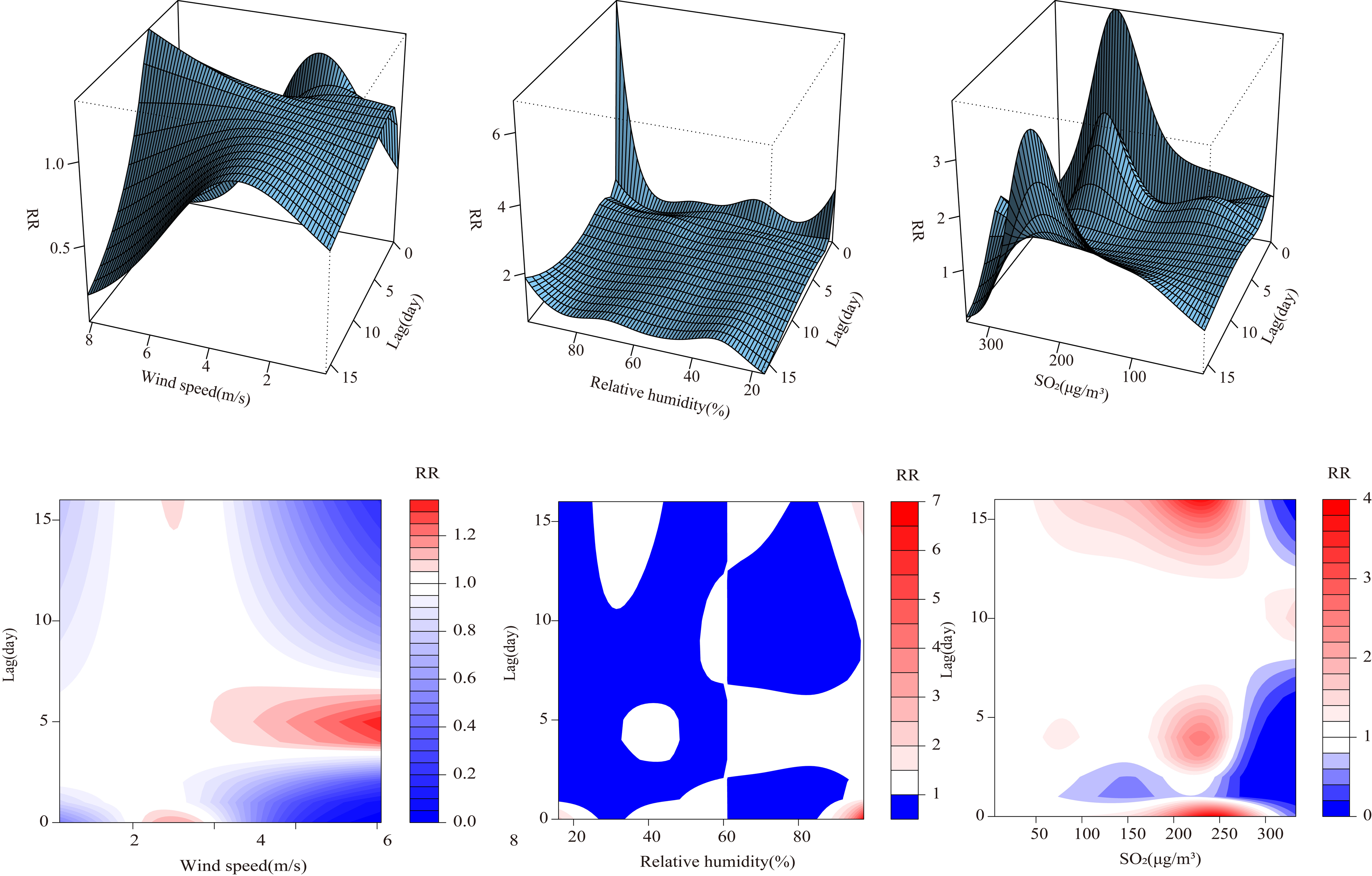

Supplement: S7 Fig — (TIF) [file pntd.0010806.s010.tif]

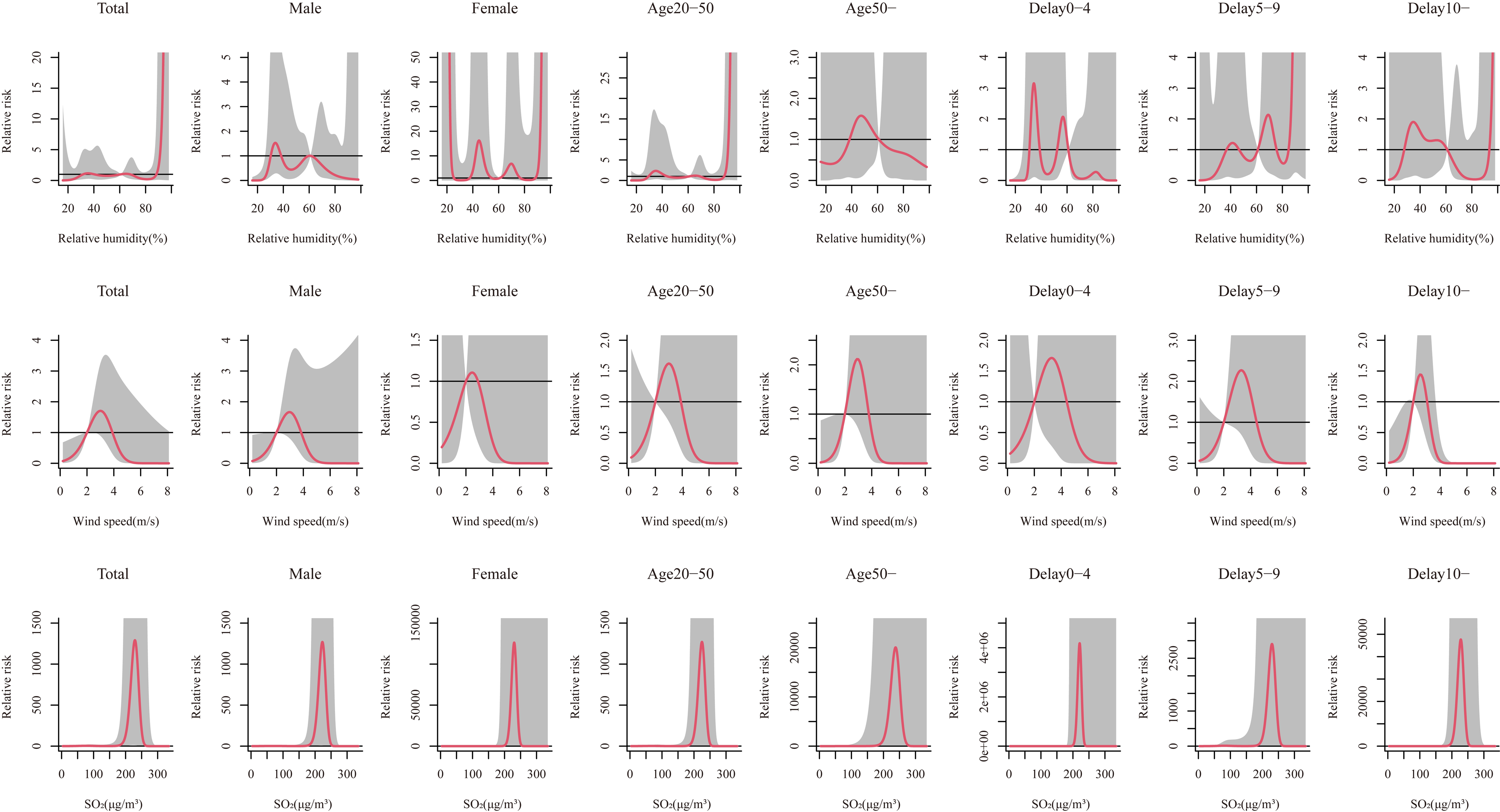

Supplement: S8 Fig — (TIF) [file pntd.0010806.s011.tif]
