# Supplementary material for: Time series analyses based on the joint lagged effect analysis of pollution and meteorological factors of hemorrhagic fever with renal syndrome and the construction of prediction model
Source: PLoS Negl Trop Dis. 2023 Jul 24;17(7):e0010806. doi: 10.1371/journal.pntd.0010806 (PMC10399869; doi:10.1371/journal.pntd.0010806)
Supplement: Attachment — Submitted filename: PNTD-D-22-01175_editorial_input.pdf [file pntd.0010806.s012.pdf]

PLOS Neglected Tropical Diseases

Prediction, impacts and interaction of meteorological and pollution variables for the development of renal syndrome hemorrhagic fever

--Manuscript Draft--

Manuscript Number:	PNTD-D-22-01175
Full Title:	Prediction, impacts and interaction of meteorological and pollution variables for the development of renal syndrome hemorrhagic fever
Short Title:	Correlation between pollutants and hemorrhagic Fever with Renal Syndrome
Article Type:	Research Article
Keywords:	HFRS, Air pollution, Distribution lag nonlinear model, Interaction
Corresponding Author:	Jing Dong<br>Department of Occupational and Environmental Health, School of Public Health, China Medical University, No.77 Puhe Road, 110122, Shenyang, Peoples' Republic of China.<br>Shenyang, CHINA
Corresponding Author Secondary Information:	
Corresponding Author's Institution:	Department of Occupational and Environmental Health, School of Public Health, China Medical University, No.77 Puhe Road, 110122, Shenyang, Peoples' Republic of China.
Corresponding Author's Secondary Institution:	
First Author:	Ye Chen
First Author Secondary Information:	
Order of Authors:	Ye Chen
	Weiming Hou
	Jing Dong
Order of Authors Secondary Information:	
Abstract:	Background:<br>Haemorrhagic fever with renal syndrome (HFRS) is a rodent-related zoonotic disease induced by hantavirus. Previous studies have identified the influence of meteorological factors on the onset of HFRS, but few studies have focused on the stratified analysis of the lagged effects and interactions of pollution and meteorological factors on HFRS.<br>Methods:<br>We collected meteorological, contaminant and epidemiological data on cases of HFRS in Shenyang from 2005-2019. A seasonal autoregressive integrated moving average (SARIMA) model was used to predict the incidence of HFRS and compared with Holt-Winters three-parameter exponential smoothing model. A distributed lag nonlinear model (DLNM) with a maximum lag period of 16 weeks was applied to assess the lag, stratification and extreme effects of pollution and meteorological factors on HFRS cases, followed by a generalized additive model (GAM) to explore the interaction of SO_2 and two other meteorological factors on HFRS cases.<br>Results:<br>The SARIMA monthly model has better fit and forecasting power than its own quarterly model and the Holt-Winters model, with an optimal model of (1,1,0) $(2,1,0)_{12}$. Overall, environmental factors including humidity, windspeed and SO_2 were correlated with the onset of HFRS and there was a non-linear exposure-lag-response association. Extremely high SO_2 increased the risk of HFRS incidence, with the maximum RR values: 2.583 (1.145,5.827). Extremely windless and low SO_2 played a significant protective role on HFRS infection, with the minimum RR values: 0.487 (0.260,0.912) and 0.577 (0.370,0.898), respectively. Interaction indicated that the risk of HFRS infection reached its highest when increasing daily SO_2 and decreasing humidity.<br>Conclusion:

	It had shown that environmental factors such as humidity and SO_2 had a delayed effect on the occurrence of HFRS. Public health professionals should take greater care in controlling HFRS in low humidity, windless conditions and 2-3 days after SO_2 levels above 200 μg/m^3.
**Suggested Reviewers:**	Xiujun Li<br>Shandong University School of Medicine: Shandong University Cheeloo College of Medicine<br>xjli@sdu.edu.cn
	Wenyi Zhang<br>Center for Disease Control and Prevention of PLA<br>zwy0419@126.com
	Sharon Daniel<br>Department of Public Health, Beer-Sheva, Israel Pediatrics and Obstetrics and Gynecology, Beer-Sheva, Israel Soroka University Medical Center<br>daniels07@gmail.com
	Qingfeng Tian<br>School of Public Health, Zhengzhou University<br>zzutqf@126.com
**Opposed Reviewers:**	
**Additional Information:**	
**Question**	**Response**
**Financial Disclosure**<br><br>Enter a financial disclosure statement that describes the sources of funding for the work included in this submission. Review the submission guidelines for detailed requirements. View published research articles from *PLOS NTDs* for specific examples.<br><br>This statement is required for submission and **will appear in the published article** if the submission is accepted. Please make sure it is accurate.	Unfunded studies

**Unfunded studies**<br><br>Enter: *The author(s) received no specific funding for this work.*<br><br>**Funded studies**<br><br>Enter a statement with the following details:<br>• Initials of the authors who received each award<br>• Grant numbers awarded to each author<br>• The full name of each funder<br>• URL of each funder website<br>• Did the sponsors or funders play any role in the study design, data collection and analysis, decision to publish, or preparation of the manuscript?<br>• **NO** - Include this sentence at the end of your statement: *The funders had no role in study design, data collection and analysis, decision to publish, or preparation of the manuscript.*<br>• **YES** - Specify the role(s) played.<br><br>* typeset	
**Competing Interests**<br><br>Use the instructions below to enter a competing interest statement for this submission. On behalf of all authors, disclose any competing interests that could be perceived to bias this work—acknowledging all financial support and any other relevant financial or non-financial competing interests.<br><br>This statement **will appear in the published article** if the submission is accepted. Please make sure it is accurate. View published research articles from *PLOS NTDs* for specific examples.	NO authors have competing interests

Powered by Editorial Manager® and ProduXion Manager® from Aries Systems Corporation

**NO authors have competing interests**<br><br>Enter: *The authors have declared that no competing interests exist.*<br><br>**Authors with competing interests**<br><br>Enter competing interest details beginning with this statement:<br><br>*I have read the journal's policy and the authors of this manuscript have the following competing interests: [insert competing interests here]*<br><br>* typeset<br><br>This statement is required for submission and **will appear in the published article** if the submission is accepted. Please make sure it is accurate and that any funding sources listed in your Funding Information later in the submission form are also declared in your Financial Disclosure statement.	
**Data Availability**<br><br>Authors are required to make all data underlying the findings described fully available, without restriction, and from the time of publication. PLOS allows rare exceptions to address legal and ethical concerns. See the PLOS Data Policy and FAQ for detailed information.	No - some restrictions will apply

A Data Availability Statement describing where the data can be found is required at submission. Your answers to this question constitute the Data Availability Statement and **will be published in the article**, if accepted.<br><br>**Important:** Stating 'data available on request from the author' is not sufficient. If your data are only available upon request, select 'No' for the first question and explain your exceptional situation in the text box.<br><br>Do the authors confirm that all data underlying the findings described in their manuscript are fully available without restriction?	
**Describe where the data may be found in full sentences. If you are copying our sample text, replace any instances of XXX with the appropriate details.**<br><br>• If the data are **held or will be held in a public repository**, include URLs, accession numbers or DOIs. If this information will only be available after acceptance, indicate this by ticking the box below. For example: *All XXX files are available from the XXX database (accession number(s) XXX, XXX.*).<br>• If the data are all contained **within the manuscript and/or Supporting Information files**, enter the following: *All relevant data are within the manuscript and its Supporting Information files.*<br>• If neither of these applies but you are able to provide **details of access elsewhere**, with or without limitations, please do so. For example:<br><br>*Data cannot be shared publicly because*	Data cannot be shared publicly because of the data secret. Data are available from the Shenyang CDC Institutional Data Access for researchers who meet the criteria for access to confidential data.

Powered by Editorial Manager® and ProduXion Manager® from Aries Systems Corporation

of [XXX]. Data are available from the XXX Institutional Data Access / Ethics Committee (contact via XXX) for researchers who meet the criteria for access to confidential data.<br><br>*The data underlying the results presented in the study are available from (include the name of the third party and contact information or URL).*<br>• This text is appropriate if the data are owned by a third party and authors do not have permission to share the data.<br><br>* typeset	
Additional data availability information:	Tick here if the URLs/accession numbers/DOIs will be available only after acceptance of the manuscript for publication so that we can ensure their inclusion before publication.

Powered by Editorial Manager® and ProduXion Manager® from Aries Systems Corporation

Dear Editor,

Enclosed for your consideration is an original research article, entitled "Prediction, impacts and interaction of meteorological and pollution variables for the development of renal syndrome hemorrhagic fever".

A seasonal autoregressive integrated moving average (SARIMA) model was used to predict the incidence of HFRS and comparing with Holt-Winters three-parameter exponential smoothing model to assess its prediction effect. Boosted regression tree (BRT) was applied to validate the fit and interaction between the factors, followed by a distributed lag nonlinear model (DLNM) with a maximum lag period of 16 weeks to assess the lag, stratification and extreme effects of pollution and meteorological factors on HFRS cases. A generalized additive model (GAM) then was established to explore the interaction of SO2 and two other meteorological factors on HFRS cases.

The SARIMA model might help to enhance the forecast of monthly HFRS incidence based on a long-range dataset. It had shown that environmental factors such as humidity and SO_2 had a delayed effect on the occurrence of HFRS and that the effect of humidity could be influenced by SO_2 and windspeed. Public health professionals should take greater care in controlling HFRS in low humidity, windless conditions and 2-3 days after SO_2 levels above 200 $\mu g/m^3$.This study may provide a reference for the establishment of a targeted HFRS control early warning system.

All authors have read the manuscript and conflict of interest statement, and approved the submission for publication; the work is original, has not been published, and is not being considered for publication elsewhere, in whole or in part, in any language. We would be very grateful if you could initiate the reviewing process. We are looking forward to hearing from you in due time.

Yours sincerely

Jing Dong

School of Public Heath, China Medical University

Shenyang, Peoples' Republic of China.

Email address: jdong@cmu.edu.cn

Manuscript Click here to access/download;Manuscript;Manuscript.doc

Prediction, impacts and interaction of meteorological and pollution variables for the development of renal syndrome hemorrhagic fever

Ye Chen [1, †], Weiming Hou [2, †], Jing Dong [2,*]

[1] Department of Infectious Disease, Shenyang Center for Disease Control and Prevention, Shenyang 110100, Liaoning Province, PR China.

[2] Department of Occupational and Environmental Health, School of Public Health, China Medical University, No.77 Puhe Road, 110122, Shenyang, Peoples' Republic of China.

† Ye Chen and Weiming Hou contributed equally to this work.

* Corresponding author: Jing Dong Email: jdong@cmu.edu.cn

Abstract

Background:

Haemorrhagic fever with renal syndrome (HFRS) is a rodent-related zoonotic disease induced by hantavirus. Previous studies have identified the influence of meteorological factors on the onset of HFRS, but few studies have focused on the stratified analysis of the lagged effects and interactions of pollution and meteorological factors on HFRS.

Methods:

We collected meteorological, contaminant and epidemiological data on cases of HFRS in Shenyang from 2005-2019. A seasonal autoregressive integrated moving average (SARIMA) model was used to predict the incidence of HFRS and compared with Holt-Winters three-parameter exponential smoothing model. A distributed lag nonlinear model (DLNM) with a maximum lag period of 16 weeks was applied to assess the lag, stratification and extreme effects of pollution and meteorological factors on HFRS cases, followed by a generalized additive model (GAM) to explore the interaction of SO_2 and two other meteorological factors on HFRS cases.

Results:

The SARIMA monthly model has better fit and forecasting power than its own quarterly model and the Holt-Winters model, with an optimal model of (1,1,0) $(2,1,0)_{12}$. Overall, environmental factors including humidity, windspeed and SO_2 were correlated with the onset of HFRS and there was a non-linear exposure-lag-response association. Extremely high SO_2 increased the risk of HFRS incidence, with the maximum RR values: 2.583 (1.145,5.827). Extremely windless and low SO_2 played a significant protective role on HFRS infection, with the minimum RR values: 0.487 (0.260,0.912) and 0.577 (0.370,0.898), respectively. Interaction indicated that the risk of HFRS infection reached its highest when increasing daily SO_2 and decreasing humidity.

Conclusion:

It had shown that environmental factors such as humidity and SO_2 had a delayed effect on the occurrence of HFRS. Public health professionals should take greater care in controlling HFRS in low humidity, windless conditions and 2-3 days after SO_2 levels above 200 μg/m^3.

Keywords:

HFRS, Air pollution, Distribution lag nonlinear model, Interaction

1.Introduction

This hantavirus-like infection has attracted world attention during the Korean War since it was first described in Chinese texts 900 years ago(Avsic-Zupancet al. 2019). Two clinical syndromes caused by hantavirus infection have been characterized: hantavirus cardiopulmonary syndrome(HCPS), prevalent mainly in America, and Hemorrhagic fever with renal syndrome(HFRS), found in Eurasia(Gutet al. 2013). HFRS(Avsic-Zupancet al. 2019), characterized by headache, fever, back pain, abdominal pain and acute renal insufficiency(Sunet al. 2021), has caused a variety of public problems, with 30,000-60,000 cases per year in mainland China in the 1990s(Zhanget al. 2010b). In Europe, over 9000 cases of HFRS are reported annually, and most cases associated with HFRS are diagnosed in parts of Europe in Russia, Finland and Sweden(Heymanet al. 2009; Vapalahtiet al. 2003). China has the highest rate of HFRS disease in the world, with domestic HFRS cases constituting 90% of the total number of cases worldwide each year(Heet al. 2018; Schmaljohn and Hjelle 1997). HFRS is a zoonotic disease associated with rodents and a legally reported disease in China(Chen and Qiu 1993).

In the past, descriptive statistical methods were mostly used for the analysis of infectious diseases such as HFRS. Nonetheless, effective prediction of short and medium term HFRS incidence can provide a reliable basis for the Center of Disease Control (CDC), as well as scientific theory and support for national infectious disease prevention and control planning(Liuet al. 2011a). With the application of big data prediction technology in various fields, it also supplies approaches for the development of HFRS prediction technology. At present, there are many ways to study data prediction technology at home and abroad, and they have been broadly applied good results(Frazeret al. 2021). Regarding infectious disease forecasting, autoregressive integrated moving average (ARIMA) and Holt-Winters models are one of the most representative and widely used models in time series forecasting(Qiuet al. 2021). Today, predictive modeling has been used in many studies in epidemiological research. Some reseachers applied the seasonal autoregressive fractionally integrated moving average (SARFIMA) model to predict renal syndrome hemorrhagic fever(Qiet al. 2020). Furthermore, a European study had applied mathematical modeling to explore the pathogenesis and impact of influenza and pathogens(Opatowskiet al. 2018).

Although the ~~amount~~ of HFRS cases dropped significantly from over 1 million cases between 1950 and 1995 to 10,000 cases in 2009, the geographical dispersion of HFRS cases further increased, with cases notified in all 31 provinces of mainland China(Xiaoet al. 2016a). Seven hantavirus serotypes/genotypes have been identified in China(Zouet al. 2008). Of these, Hantaan virus and Seoul virus are the main pathogens of HFRS, and cases caused by Hantaan virus account for about 70% of domestic HFRS cases(Zhanget al. 2011). Climate and environmental changes might impact the reservoir ecology and dynamics of rodent carriers, thereby triggering the spread of hantavirus transmission(Tianet al. 2017; Zhanget al. 2010b).

Climatic conditions are broadly regarded as one of the most pivotal factors

affecting rodent population dynamics and contributing to more cases of HFRS in humans(Liuet al. 2011b). Some studies have found a relevance of climatic factors to HFRS. For instance, a systematic evaluation of climate variability and human hantavirus infection in Europe was previously carried out by J. Roda Gracia et al. In 2010, some researches in China used time-series Poisson regression model to examine the independent effect of climate variables on the spread of HFRS, pointing out the important role of climate variation in the transmission of HFRS in northeastern China(Zhanget al. 2010a).It will contribute to future international discussions on zoonotic diseases in the context of climate change(Roda Graciaet al. 2015). Yet little research has been done on the correlation between pollutants and HFRS epidemics. And few studies have synthesized the lagged effects of diverse environmental variables on the onset of HFRS and analyzed the interactions among them. Therefore, we explored the relationship between meteorological and pollutant factors and the onset of HFRS, and speculated that the interaction among environmental factors is of attention ~~for the onset of~~ HFRS.

In this study, the incidence rate was predicted by comparison using time series analysis using HFRS surveillance data in Shenyang, followed by the application of Boosted regression tree (BRT) verifing the fit and interaction among the environmental factors, and the lag effect and interaction of meteorological and pollutant factors were investigated using distributed lag nonlinear model (DLNM) and generalized additive model (GAM).

2.Methods

2.1 Setting

Shenyang is the capital city of Liaoning Province. It is a district-level city in China, covering both urban and rural locations. Shenyang is located in latitude 41°11′–43°02′N and longitude 122°25′–123°48′E, measures 12,860 Sq km and composed of 13 districts and 214 towns(Dai and Zhang 2018). In 2019, Shenyang City's average population was 7,511,923. Shenyang city belongs to the temperate semi-humid continental climate zone. The geographical situation of Shenyang is indicated in Figure S1.

2.2 Data collection

We obtained surveillance data on cases of HFRS in Shenyang between 2005 and 2019 from CDC of Shenyang. All patients were diagnosed according to the Criteria and Management Principles of Renal Syndrome Hemorrhagic Fever issued by the Ministry of Health. We obtained the corresponding daily weather data from the China Meteorological Data Sharing Service (www.data.cma.cn). Meteorological, pollutant information were originally from the National Oceanic and Atmospheric Administration (NOAA) and 11 state-controlled environmental air quality automatic monitoring stations through the website of Shenyang Bureau of Ecology and Environment.

2.3 SARIMA and Holt-Winters model construction

Based on the quarterly and monthly data series, additive and multiplicative models were adopted to build factor decomposition models respectively, followed by the application of simple central moving average method to decompose the following four factor maps respectively: (1) long-term trend. (2) cyclical fluctuations. (3) seasonal variations. (4) random fluctuations. Auto regressive integrated moving average model(Liuet al. 2011a) is a time series forecasting method proposed by Geogre Box and Gwilym Jenkins. The ARIMA model is a classical time-series analysis method and is extensively used. The SARIMA model is developed on the foundation of the ARIMA model. The SARIMA model is based on a further development of the ARIMA model, which is particularly suitable for cases where both trend and seasonality are present in the series. The SARIMA model is abbreviated as SARIMA (P, D, Q) $_S$, where p and q are the orders of autoregressive and moving average, P and Q are the orders of seasonal autoregressive and moving average, d is the number of variances, D is the number of seasonal variances, and S is the seasonal period and cycle length(Chen and Qiu 1993). The construction of the SARIMA model is shown in Equation (1).

$$
\begin{aligned}
&\Phi(L)A_P\left(L^s\right)\left(\nabla^d\nabla_s^D x_i\right)=\Theta_q(L)B_q\left(L^s\right)\varepsilon_t,\\
&E\left(\varepsilon_t\right)=0,\operatorname{Var}\left(\varepsilon_t\right)=\sigma_s^2,E\left(\varepsilon_t\middle|\ \varepsilon_s\right)=0,s\neq t,\\
&E\left(x_s\varepsilon_t\right)=0,s<t
\end{aligned}
$$

where L is the delay operator, $A_P(L^s)$ is the p-order autoregressive operator, $A_q(L^s)$ is the q-order seasonal moving average operator, ∇^d=(1-L)^d is the difference operation, and ∇_s^D=(1-L^s)^d is the seasonal difference operation. The order of approximation of the model is determined based on the autocorrelation function. The QAIC information criterion is then used to determine the best combination of parameters for the model, and the model satisfies the residual white noise test.

The Holt-Winters model is a forecasting technique proposed by Holt and Winters in 1960 that is based on speculative smoothing. Unlike ARIMA, Holt's linear equation has a built-in equation for seasonal factors that directly captures seasonality(Zhuet al. 2019).

Three smoothing equations are used to calculate and evaluate deseasonalized series, trends and seasonality variables. The Holt–Winters' additive method can be written as follows:

$$L_t=\alpha\left(y_t-S_{t-s}\right)+(1-\alpha)\left(L_{t-1}+b_{t-1}\right)$$

$$S_t=\delta\left(y_t-L_t\right)+(1-\delta)S_{t-1}\ b_t=\gamma\left(L_t-L_{t-1}\right)+(1-\gamma)b_{t-1}$$

$$S_t=\delta\left(y_t-L_t\right)+(1-\delta)S_{t-1}$$

where t =1, ..., n, S represents the length of seasonality (months), L_t represents the level of the series, and b_t denotes the trend and seasonal components. The constants used in this model are α (horizontal smoothing constant), γ (trend smoothing

constant) and δ (seasonal smoothing constant)(Harrisonet al. 2012).

2.4 BRT model construction

BRT methods have been successfully applied to research fields such as disease modeling(Mazaheriet al. 2017). The BRT method produces a series of trees, each of which grows on the remnant of the previous tree. In this case, the predictions are obtained by weighting the grouped responses of the entire regression tree(Hussainet al. 2021). In the BRT model, f(x) is an evaluation of the response y based on a vector predictor of x, which in turn is integrated as an additive form of b(x; γ_m), as follows:

$$f(x) = \sum_m f_m(x) = \sum_m \beta_m b(x; \gamma_m)$$

where β_m is the expansion factor and $b(x; \gamma_m)$ is a simpli-categorical function with parameters y and variables x. The coefficient β_m reflects the weights allocated to the nodes of each tree and identifies the type of combination predicted for each tree. In this approach, the three regularization parameters, number of trees , learning rate (lr) and tree complexity (tc), should be optimized. The complexity (tc) should be optimized. To this purpose, in this study, various nt, tc (1-10), and lr (0.001, 0.05, and 0.01) are allocated to the training of the BRT model in order to maximize the model performance(Foroughiet al. 2020).

2.5 DLNM model and GAM construction

DLNM has been extensively used to assess the exposure-lag-response relationship between environmental factors and human diseases, such as congenital heart disease, HFRS, non-accidental deaths and so on(Caoet al. 2021; Zhanget al. 2021). The model can be written as follows:

$$\log\left[E\left(Y_t\right)\right] = \alpha + NS(M, df, \text{lag}, df) + \Sigma NS\left(X_i\right) + NS(\text{ Time }, df) + \beta DOW_t + \gamma \text{ Holiday }_t$$

To analyze the lag and extreme effects of environmental factors, Humidity, Windspeed and SO_2 were taken and applied to the cross-basis functions of DLNM. Here, Y_t was the number of daily counts of HFRS cases in daily t; α was the intercept of the whole model; *NS* is a natural cubic spline that acts as a smooth function of the model; *M* represents the estimated environmental variable related to HFRS; X_t is the other environmental variables in the pathogenesis of HFRS that requires nonlinear confounding effect adjustment; *NS* was used to adjust for daily confounding in the model; *DOW* is a categorical variable for day of week; Holiday is a binary variable used to control the effect of chinese public holidays, β and γ are the regression coefficients; The optimal degrees of freedom (*df*) for the spline function were estimated by Akaike information criterion for quasi-Poisson (Q-AIC) and Minimum partial regression coefficient ($PACF_{min}$) criteria; NS of 4,6 and 8 *df* were used for windspeed, SO_2 and relative humidity respectively, and the lag space was set to 3 *df*. NS with 5*df*/year was applied to time variable. In addition, as the incubation period for human hantavirus infection is typically 7-14 days, our model applied the Q-AIC guidelines using a delay of up to 16 days.

In our study, the median environmental variable has been used as a reference

value to compute the relative risk. Furthermore, We estimated the effect of extreme environmental factors on the incidence of HFRS by comparing the 25th or 75th percentile of environmental variables with the median. The impact of environmental factors was analyzed by stratifying by gender, age group and number of diagnostic delayed days in order to identify susceptible populations and their corresponding sensitivities.

Subsequently, the GAM method was used to explore the interaction of meteorological and pollutant factors on the prevalence of HFRS. The model can be written as follows:

$$\log[E(Yt)] = \alpha 2 + s_1(X_1, X_2) + s_2(X_3) + s_3(\text{ day })$$

$\alpha 2$ is the intercept; X_1 indicates one of the environmental factors (Humidity, Windspeed and SO_2) whereas X_2 and X_3 denote the other two; s () indicates penalized spline function. $s_1(X_1, X_2)$ is a spline function of the interaction between the parameters X_1 and X_2. (X_1, X_2, X_3 are all 16 lagged variables.)

2.6 Statistical analysis

As there were missing values in the incidence data of HFRS in Shenyang, we performed linear interpolation to compensate as soon as possible in order to better apply Box-Jenkins and exponential smoothing methods for incidence prediction. For the influence of meteorological and pollutant factors on the number of HFRS cases, Spearman correlation analysis was used for feature selection, followed by BRT to fit the selected features to the variables and interaction tests. We developed a DLNM with a maximum lag of 16 weeks to evaluate the lagged, stratification and extreme effects of pollution and meteorological factors on the cases of HFRS. A GAM then was established to explore the interaction of SO_2 and two other meteorological factors on HFRS cases.

All analyses in our study were performed in R software (version 4.1.3).

3.Results

3.1 Descriptive characteristics of HFRS cases and environmental factors

A total of 1,880 cases of HFRS were reported in Shenyang from 2005-2019, of which the incidence of HFRS was predominantly in young adults aged 20-50, accounting for 71.81% of all cases. Men are more sensible than women at a ratio of 3.67:1 (1477:403). By the end of 2019, the prevalence of HFRS in Shenyang was 25.03(10^{-5}) %, and the mortality rate was 0.691% (Table 1). The onset of HFRS showed significant differences in seasonality, age, and delayed days in diagnosis of onset (p<0.05) (Table S1). Summary statistics of all HFRS cases and environmental variables in Shenyang are shown in Table S2. Figure 1 shows the time series distribution of daily cases of HFRS and environmental factors from 2005-2019. There are distinct seasonal variations in both HFRS and environmental conditions.

3.2 Time-series analysis of HFRS% of Holt-Winters and SARIMA model in monthly and seasonal prediction.

From the factor decomposition diagrams in Figure S2, it can be seen that the annual incidence rate of HFRS is trending down, and after removing the trend effect from the original series, the difference in the average seasonal index among different quarters is the difference caused by the seasonal effect. The incidence rate of HFRS in Shenyang is in the 1st and 4th quarters every year. The monthly HFRS has the characteristics of bimodal distribution, with the first peak in March-May, the second peak in November-December, and the seasonal inelasticity rising from April-August. The incidence of HFRS in Shenyang is characterized by a bimodal monthly distribution, with the first peak in March-May, the second peak in November-December, and an exponential decline in April-August, and a seasonal inverse rise starting in September.

The model fixed-order plots in Figures S3 and S4 allow the SARIMA seasonal and monthly models to be parameterised for the ACF, PACF plots, combined with the “auto.arima()” function to correct for the AR and MA parameters (parameter estimates < 2 times the sample standard deviation). The model parameters and tests are shown in Table 2, it gives the forecasting accuracy of two models for the HFRS series. The SARIMA model has lower values for RMSE, MAE and MAPE, which means the SARIMA is more accurate. After modifying the model according to the model parameters, the incidence of HFRS from 2005-2018 was used as the training set and 2019 as the validation set, and the Holt-Winters and SARIMA models were applied to predict the incidence of HFRS, respectively, and the prediction effects were plotted as obtained in Figure 2 and Table S3. From them it can be seen that the Holt-Winters model predicts trends closer to the actual values than the SARIMA model. the 95% confidence interval for the SARIMA model is narrower than the Holt-Winters model and its interval contains all the actual values. Figure S5-S6 show the tests of goodness of fitness and significance for the series of HFRS incidence from the two methods.

3.3 Feature selection and fitting of environmental factors for HFRS

Spearman correlation analysis showed that HFRS was significantly correlated with Humidity ($r=-0.10$, $p<0.01$), Windspeed ($r=0.07$, $p<0.05$) and SO_2 ($r=0.09$, $p<0.01$) (Table S4). Furthermore, to fit the BRT model, we set the model parameters: tree complexing was 5, learning rate was 0.005, and bag.fraction was 0.5. According to Figure S7, it can be seen that the degree of fit of each of the three environmental factors fitting functions was similar to the Spearman correlation results, and the trend with the number of HFRS incidence was significant.

Depending on the distribution of observations in the environment space, the fitting function can give a distribution of fitted values relating to each predictor. The values at the top of each graph indicate the weighted average of the fitted values associated with each non-factor predictor. According to the interaction fitting function in Figure 5, it is known that environmental factors fit better at moderate levels of interaction.

3.4 The lag relationship between environmental variables and the incidence of HFRS

Figure S8 shows that the non-linear exposure–lag–response association among daily humidity, windspeed, SO_2 and HFRS incidence cases, which indicated that these factors are ~~at~~ high relative risk at lags above moderate levels. Different lag times correspond to different effects, specifically the effect of low wind speed occurs rapidly but lasts for a short time, the effect of high wind speed has a longer lag time but has a greater impact, while high humidity can have a transient effect on HFRS and high levels of SO_2 can have a transient or continuous effect on HFRS, with the effect initially concentrated in the lag time of 0-5 days and after 15 days.

Estimates of the impact of meteorological and pollutant factors on HFRS cases show varied lagging modes. Figure S9 shows the overall effect of environmental variables for total, gender, age and delayed-days HFRS cases within 16 days. Overall, these meteorological and pollutant variables were significantly relevant to HFRS cases. We found that RRs increased with the improvement of humidity, windspeed, and SO_2, suggesting that higher humidity, windspeed, and SO_2 increased the risk of HFRS. Yet humidity and SO_2 separately reached the peak at 98% and 229.1μg/m^3, then began to decrease or stabilize. Windspeed ~~arrived~~ the peak at 3m/s and ~~start~~ decreas~~ing~~. In general, similar trends in exposure-response relationships between environmental variables and cases of HFRS disease by gender, age and delayed-days group compared to total cases are shown in Figure S9. The minimum risk of incidence (RR_{min} values for environmental factors such as humidity, windspeed and SO_2 were 16%, 8.1m/s and 223μg/m^3 respectively.

Generally, analogous trends in exposure-response and lag-response relationships among environmental variables and HFRS cases across gender, age, and delay days of groups compared to total cases are shown in Figures S9, 3, and 4.

3.5 Exposure-response relationships for environmental factors with different lag times

The effects of humidity, SO_2 on HFRS differed across lag times and stratification factors when the study lag time points were 0 and 16 days. The RR of the effect of humidity on HFRS cases tended to increase at lag 0 and 16 days, with humidity RR values reaching a maximum at 20-40% and above 90% at lag 0, while lag 16 days only showed a maximum RR value at high humidity. The effect values of windspeed and SO_2 on HFRS cases at lags 0 and 16 days showed a trend of increasing and then decreasing RR, with RR values in the range of 2-4m/s and 200-250μg/m^3, respectively. Within the different grouping intervals, the trend of RR effect values for humidity within Delay 0-4 days was slightly different from the overall, and the RR effect values for windspeed within Female, Delay 0-4, Delay 5-9 days, and SO_2 within Delay 0-4 days were significantly different.

3.6 Effects of extreme environmental variables on HFRS cases

To determine the effect of extreme environmental factors on the HFRS, the estimated effects were examined by comparing the 25th or 75th percentile of relative humidity, wind speed and SO_2 with their median values. Table 3 shows the cumulative impact of the lag factor extremes on the HFRS at 16 lag days. We found that extreme

high levels of SO_2 were positively linked to the onset of HFRS, while extreme low levels of SO_2, with no wind effect, had a protective effect, and the RR values of cumulative effects of were 2.583 (1.145,5.827) for high SO_2 effect and 0.577 (0.370, 0.898) for cold SO_2 effect. At 16 lag days, significant cumulative effects of windless conditions were observed in males (RR value: 0.490 (0.241,0.997)), in the over 50 years age group (RR value: 0.335 (0.113,0.992)) and in delayed onset for over 10 days (RR value: 0.324 (0.106,0.983)).In turn, women at extreme SO_2 levels and patients with a delayed onset of 5-9 days are susceptible, with their RR values: 8.122 (1.009,65.403) for high SO_2 and 0.285 (0.090,0.898) for low SO_2, 4.491(1.246,16.193) for high SO_2 and 0.427(0.213, 0.858) for low SO_2, respectively.

The distributed lagged effects of extreme environmental factors at various lag days for all groups were showed in Fig 4. We found that the dry effect indicated a maximum RR value on the current day, peaking at 4 lag days and then showed a U-shaped curve along the lag days, and the RR value subsequently decreased for the next days and then turned to rise along the lag days, whereas wet effect showed the opposite trend. The curve of dry and wet effect was roughly similar among different stratified groups. On the windless effect, the 2 lag days is a peak followed by a decline, while the opposite is true for the windy effect. The overall patients reached their highest effect at extreme high or low levels of SO_2, usually at a lag of 1 day, followed by a gradual downward trend. female, aged >1 years and delayed 5-9 days remain the most sensitive people.

Table 4 indicates the cumulative relative risk of HFRS cases related to extreme environmental variables at various lags. Overall, the high level SO_2 effect was positively related to the risk of developing HFRS, and the cumulative impact increased with the number of lag days. The cumulative RR values gradually decreased as the number of lagging days rose. There was no significant correlation within the lag days for humidity and windy effects with HFRS. The windless effect and low levels of SO_2 were protective after 0 and 15 days of lagging.

3.7 Environmental interaction during humidity, windspeed, SO_2 and HFRS cases

Statistically significant GAMs were built to show the interaction effect among humidity, windspeed and SO_2 on HFRS incidence (Figure 5). The program on the top side of Fig 5 shows the interaction effect of windspeed and humidity on HFRS. The HFRS infection risk increased as daily windspeed and humidity decreased. The plot to the bottom of Fig 5 indicates the interaction effect of SO_2 and humidity, HFRS tends to occur in higher SO_2 and lower humidity environmental conditions.

4.Discussion

From the time series and seasonal decomposition of the incidence of HFRS in Shenyang, combined with studies in various regions of China(Wuet al. 2020), it can be seen that there is a clear seasonal trend in the incidence of HFRS in China, with a decreasing trend year upon year. The peak in case reporting differs between single and double peaks across regions, with the main peak occurring between March and May each year. This study also shows a second peak in November to December, which may occur for the following reasons: (1) The peak of population movement is about March each year, when the mobility of urban life becomes more complex and the fast pace of life and consumption leads to a gradual decline in the demand for health. (2) After November, the cooler temperatures in the city lead to larger crowds and a greater temperature difference between indoors and outdoors, allowing host animals to enter human life more closely and the disease to be more contagious.

Currently, many scholars have conducted research on predictive models for the onset of infectious diseases. The SARIMA model had been used to predicted the incidence of HFRS(Qiet al. 2020; Sun and Zou 2018). It shows that SARIMA has the characteristics of being unconstrained by data type and high applicability, integrating factors such as trend, periodicity and random error, so that it can be used in prediction studies of infectious diseases with periodic morbidity characteristics. Pritthijit Nathet al.applied both the SARIMA model and the Holt-winters seasonal model for the prediction of airborne particulate matter in eastern India, the Holt - winters model was considered to be simple in principle and had a high predictive accuracy for diseases with a cyclical pattern of onset(Nathet al. 2021). In this study we discussed the effect of the SARIMA model applied to the HFRS series and compared it with the Holt-Winters model. In terms of model mechanism, the SARIMA model is suitable for predicting series that are smooth and stable over time compared to the Holt - winters model, which is suitable for predicting models with a single trend of change. However, research on time series has limitations, as both methods in this study are extrapolated forecasts based on historical data, usually considering the characteristics of the series itself, and cannot predict sudden changes in the data due to changes in external factors. Moreover, the occurrence and prevalence of infectious diseases are influenced by multiple natural factors, climate and other social factors. Our study thus explored the lagging, interactive and stratified effects of meteorological and pollutant factors on the prevalence of HFRS in Shenyang.

Firstly, we need to select and fit the features using the ~~spearman~~ method combined with a BRT. The results were chosen from three environmental factors, windspeed, humidity and SO_2, in order to achieve a precise study of the influence of environmental factors on HFRS. Subsequently, the DLNM method was applied to examine the exposure-lag-response relationship between the average daily cases of HFRS disease and environmental factors in Shenyang from 2014-2019. The results showed a non-linear lagged relationship among meteorological, pollutant factors and HFRS. Extremely high concentration levels of SO_2 increased the risk of HFRS, while low wind speed and low concentration levels of SO_2 were protective against HFRS

from 0-16d. It was also found that the lagged effects of different climatic and pollutant factors were not identical. The different delay periods reflect the fact that the lagged effect of each environmental variable may be related to the spread of infection influenced by various factors, including the growth of the virus in the external environment, the inclination of people to go outside, and seasonal changes in rodent populations(Zhanget al. 2010a; Zhaoet al. 2019). We demonstrated that high concentrations of SO_2 significantly influenced the spread of HFRS after 0-5 and 15 lag days. Extreme low wind speeds were strongly associated with HFRS from lag 0 to a maximum lag 16 days, suggesting that the incidence of HFRS may be lagged by approximately 16 days at low windspeed. It could be similar to the study by Zhang(Zhanget al. 2016) et al. on HFMD, mainly because low windspeed may inhibit the spread of hantavirus-containing particles(Lighthart and Mohr 1987). The effect of humidity on HFRS cases tended to increase at days 0 and 16 when the lag time was 16 days, with the greatest effect at 20-40% at lag 0 days and at over 90% at lag 16 days. Higher humidity levels may indicate that humidity affects the survival of the rodent host, in addition to affecting the infection and stability of the virus in the in vitro environment(Zhanget al. 2010a). Furthermore, in contrast to previous studies, our study included pollutants in the HFRS influencing factors and explored the non-linear lag between SO_2 and HFRS. Our study found that elevated SO_2 concentrations increased the risk of HFRS infection at levels in the range of 200-250 $\mu g/m^3$ and were significant in women and in patients with a delayed onset of 5-9 days. There is still controversy about the effect of SO_2 on HFRS, which may be related to regional, population differences and the proportion of pollutants in the air. However, regarding the effect of SO_2 on other infectious diseases, there were different reports showing a significant protective effect of SO_2 against influenza(Liet al. 2021)(RR=0.892, 95% CI: 0.840-0.948), which probably due to the higher outdoor pollutants, resulting in a population more dependent on the indoor environment and less exposed to the virus.

Stratified analysis showed that the effects of meteorological and pollutant factors varied by sex, age group, and number of days delayed onset. Men were more sensitive to extreme low wind speeds than women, and women were more sensitive to extreme SO_2 concentration levels than men. Similar results have been reported in several studies on other infectious diseases(Liet al. 2021).Patients over 50 years were more significantly affected by extreme low wind speeds and showed a protective effect, as compared to other age groups. But based on previous studies, windspeed effects were generally significantly associated with lower age(Yuet al. 2019). This study showed that HFRS mainly affected people under 50 years of age at low windspeed, which might be attributed to underlying factors such as social factors, population distribution, etc. In terms of delay days, patients with a delay of 5-9 days were more sensitive to extreme SO_2 concentrations and patients with a delay of over 10 days were more susceptible to extreme low windspeed. The delay between the onset of HFRS and the time of diagnosis led to a lagging effect of environmental factors reinforced by the length of time the patient spent in the environment after onset. Additionally, we did not take temperature, barometric pressure into account in our study of the correlation

between environmental factors and HFRS. Some studies had shown that mean and extreme temperatures were negatively correlated with cases of HFRS(Liuet al. 2011b). This study did not discuss the relationship with HFRS cases in terms of temperature, barometric pressure, and rainfall factors, showing different findings of HFRS in Shenyang before 2011 and after 2014, suggesting possible spatial and temporal variability.

The results of the interaction analysis showed that higher SO_2 and lower humidity environments were the dangerous environmental conditions for the occurrence of HFRS. It was demonstrated that NO_X and SO_2 in the air showed strong seasonal variations and that their concentrations were closely related to meteorological factors such as wind speed, temperature and relative humidity. Air pollution may impact the frequency of HFRS cases by modifying viral infectivity and immunity in humans and rodents(Ciencewicki and Jaspers 2007; Weber and Stilianakis 2008).The combined effect of low windspeed and low humidity also affected the development of HFRS disease. Analysis of the geographical distribution of the country suggested that this result could be attributed to the region's location in a climatic zone(Xiaoet al. 2016b).

Our research benefits cover: (1) the study period is long, and the study collected case and environmental data over many years. (2) For the time series analysis, we applied two different models for comparison and also split the data into monthly and seasonal data for accurate comparison and forecasting. (3) Our study applies advanced statistical methods, not only applying spearman to feature selection, but also applying the BRT method to fit the screened variables and their interactions, followed by DLNM and GAM to analyse the lagged, extreme and cumulative effects of environmental factors. Our findings can provide evidence and guidance on the lagged effects and interactions of environmental factors on HFRS. It is worth pointing out that there were some limitations to our study. Firstly, there are cases of HFRS in this study that have been diagnosed both clinically and through the laboratory, which may be subject to diagnostic bias and are under-reported. Moreover, due to the regional limitations of this study, other regions should be referred to with caution in studying the impact and prediction of HFRS disease, taking into account regional characteristics, and making changes in model selection, parameters and factor selection.

5.Conclusion

The SARIMA model may help to enhance the forecast of monthly HFRS incidence based on a long-range dataset. Our study had shown that environmental factors such as humidity and SO_2 have a delayed effect on the occurrence of HFRS and that the effect of humidity can be influenced by SO_2 and windspeed. Public health professionals should take greater care in controlling HFRS in low humidity, windless conditions and 2-3 days after SO_2 levels ~~above~~ 200 μg/m^3.

References

Avsic-Zupanc, T.; Saksida, A.; Korva, M. Hantavirus infections. Clin Microbiol Infect 2019;21S:e6-e16

Cao, R.; Wang, Y.; Huang, J.; He, J.; Ponsawansong, P.; Jin, J.; Xu, Z.; Yang, T.; Pan, X.; Prapamontol, T.; Li, G. The Mortality Effect of Apparent Temperature: A Multi-City Study in Asia. Int J Environ Res Public Health 2021;18

Chen, H.X.; Qiu, F.X. Epidemiologic surveillance on the hemorrhagic fever with renal syndrome in China. Chin Med J (Engl) 1993;106:857-863

Ciencewicki, J.; Jaspers, I. Air pollution and respiratory viral infection. Inhal Toxicol 2007;19:1135-1146

Dai, M.Y.; Zhang, C.Q. Analysis on epidemiological characteristics of scarlet fever,Shenyang city,2013-2017. Preventive Medicine Tribune 2018;

Foroughi, M.; Ahmadi Azqhandi, M.H.; Kakhki, S. Bio-inspired, high, and fast adsorption of tetracycline from aqueous media using Fe(3)O(4)-g-CN@PEI-β-CD nanocomposite: Modeling by response surface methodology (RSM), boosted regression tree (BRT), and general regression neural network (GRNN). J Hazard Mater 2020;388:121769

Frazer, J.; Notin, P.; Dias, M.; Gomez, A.; Min, J.K.; Brock, K.; Gal, Y.; Marks, D.S. Disease variant prediction with deep generative models of evolutionary data. Nature 2021;599:91-95

Gut, A.K.; Gut, R.; Pencuła, M.; Jarosz, M.J. New cases of suspected HFRS (Hantavirus infection) in south-eastern Poland. Ann Agric Environ Med 2013;20:544-548

Harrison, E.; Uchendu, B.; Victor-Edema, U. Arima Fit to Nigerian Unemployment Data.

2012;

He, J.; Christakos, G.; Wu, J.; Cazelles, B.; Qian, Q.; Mu, D.; Wang, Y.; Yin, W.; Zhang, W. Spatiotemporal variation of the association between climate dynamics and HFRS outbreaks in Eastern China during 2005-2016 and its geographic determinants. PLoS Negl Trop Dis 2018;12:e0006554

Heyman, P.; Vaheri, A.; Lundkvist, Å.; Avsic-Zupanc, T. Hantavirus infections in Europe: from virus carriers to a major public-health problem. Expert Review of Anti-infective Therapy 2009;7:205-217

Hussain, S.; Khan, H.; Gul, S.; Steter, J.R.; Motheo, A.J. Modeling of photolytic degradation of sulfamethoxazole using boosted regression tree (BRT), artificial neural network (ANN) and response surface methodology (RSM); energy consumption and intermediates study. Chemosphere 2021;276:130151

Li, X.; Xu, J.; Wang, W.; Liang, J.J.; Deng, Z.H.; Du, J.; Xie, M.Z.; Wang, X.R.; Liu, Y.; Cui, F.; Lu, Q.B. Air pollutants and outpatient visits for influenza-like illness in Beijing, China. PeerJ 2021;9:e11397

Lighthart, B.; Mohr, A.J. Estimating downwind concentrations of viable airborne microorganisms in dynamic atmospheric conditions. Appl Environ Microbiol 1987;53:1580-1583

Liu, Q.; Liu, X.; Jiang, B.; Yang, W. Forecasting incidence of hemorrhagic fever with renal syndrome in China using ARIMA model. BMC Infect Dis 2011a;11:218

Liu, X.; Jiang, B.; Gu, W.; Liu, Q. Temporal trend and climate factors of hemorrhagic fever with renal syndrome epidemic in Shenyang City, China. BMC Infect Dis 2011b;11:331

Mazaheri, H.; Ghaedi, M.; Ahmadi Azqhandi, M.H.; Asfaram, A. Application of machine/statistical learning, artificial intelligence and statistical experimental design for the modeling and optimization of methylene blue and Cd(ii) removal from a binary aqueous solution by natural walnut carbon. Phys Chem Chem Phys 2017;19:11299-11317

Nath, P.; Saha, P.; Middya, A.I.; Roy, S. Long-term time-series pollution forecast using statistical and deep learning methods. Neural Comput Appl 2021;33:12551-12570

Opatowski, L.; Baguelin, M.; Eggo, R.M. Influenza interaction with cocirculating pathogens and its impact on surveillance, pathogenesis, and epidemic profile: A key role for mathematical modelling. PLoS Pathog 2018;14:e1006770

Qi, C.; Zhang, D.; Zhu, Y.; Liu, L.; Li, C.; Wang, Z.; Li, X. SARFIMA model prediction for infectious diseases: application to hemorrhagic fever with renal syndrome and comparing with SARIMA. BMC Med Res Methodol 2020;20:243

Qiu, H.; Zhao, H.; Xiang, H.; Ou, R.; Yi, J.; Hu, L.; Zhu, H.; Ye, M. Forecasting the incidence of mumps in Chongqing based on a SARIMA model. BMC Public Health 2021;21:373

Roda Gracia, J.; Schumann, B.; Seidler, A. Climate Variability and the Occurrence of Human Puumala Hantavirus Infections in Europe: A Systematic Review. Zoonoses Public Health 2015;62:465-478

Schmaljohn, C.; Hjelle, B. Hantaviruses: a global disease problem. Emerg Infect Dis 1997;3:95-104

Sun, L.; Zou, L.X. Spatiotemporal analysis and forecasting model of hemorrhagic fever with renal syndrome in mainland China. Epidemiol Infect 2018;146:1680-1688

Sun, W.; Liu, X.; Li, W.; Mao, Z.; Sun, J.; Lu, L. Effects and interaction of meteorological factors on hemorrhagic fever with renal syndrome incidence in Huludao City, northeastern China, 2007-2018. PLoS Negl Trop Dis 2021;15:e0009217

Tian, H.; Yu, P.; Bjørnstad, O.N.; Cazelles, B.; Yang, J.; Tan, H.; Huang, S.; Cui, Y.; Dong, L.; Ma, C.; Ma, C.; Zhou, S.; Laine, M.; Wu, X.; Zhang, Y.; Wang, J.; Yang, R.; Stenseth, N.C.; Xu, B. Anthropogenically driven environmental changes shift the ecological dynamics of hemorrhagic fever with renal syndrome. PLoS Pathog 2017;13:e1006198

Vapalahti, O.; Mustonen, J.; Lundkvist, Å.; Henttonen, H.; Plyusnin, A.; Vaheri, A. Hantavirus Infections in Europe. The Lancet Infectious Diseases 2003;3:653-661

Weber, T.P.; Stilianakis, N.I. Inactivation of influenza A viruses in the environment and modes of transmission: a critical review. J Infect 2008;57:361-373

Wu, G.; Xia, Z.; Wang, F.; Wu, J.; Cheng, D.; Chen, X.; Liu, H.; Du, Z. Investigation on risk factors of haemorrhagic fever with renal syndrome (HFRS) in Xuancheng City in Anhui Province, Mainland China. Epidemiol Infect 2020;148:e248

Xiao, H.; Huang, R.; Gao, L.-D.; Huang, C.-R.; Lin, X.-L.; Li, N.; Liu, H.-N.; Tong, S.-L.; Tian, H.-Y. Effects of Humidity Variation on the Hantavirus Infection and Hemorrhagic Fever with Renal Syndrome Occurrence in Subtropical China. The American Society of Tropical Medicine and Hygiene 2016a;94:420-427

Xiao, H.; Huang, R.; Gao, L.D.; Huang, C.R.; Lin, X.L.; Li, N.; Liu, H.N.; Tong, S.L.; Tian, H.Y. Effects of Humidity Variation on the Hantavirus Infection and Hemorrhagic Fever with Renal Syndrome Occurrence in Subtropical China. Am J Trop Med Hyg 2016b;94:420-427

Yu, G.; Li, Y.; Cai, J.; Yu, D.; Tang, J.; Zhai, W.; Wei, Y.; Chen, S.; Chen, Q.; Qin, J. Short-term effects of meteorological factors and air pollution on childhood hand-foot-mouth disease in Guilin, China. Sci Total Environ 2019;646:460-470

Zhang, Q.; Sun, S.; Sui, X.; Ding, L.; Yang, M.; Li, C.; Zhang, C.; Zhang, X.; Hao, J.; Xu, Y.; Lin, S.; Ding, R.; Cao, J. Associations between weekly air pollution exposure and congenital heart disease. Sci Total Environ 2021;757:143821

Zhang, W.Y.; Guo, W.D.; Fang, L.Q.; Li, C.P.; Bi, P.; Glass, G.E.; Jiang, J.F.; Sun, S.H.; Qian, Q.; Liu, W.; Yan, L.; Yang, H.; Tong, S.L.; Cao, W.C. Climate variability and hemorrhagic fever with renal syndrome transmission in Northeastern China. Environ Health Perspect 2010a;118:915-920

Zhang, X.; Chen, H.Y.; Zhu, L.Y.; Zeng, L.L.; Wang, F.; Li, Q.G.; Shao, F.J.; Jiang, H.Q.; Liu, S.J.; Ma, Y.J.; Zhu, Y.; Ma, Y.J. Comparison of Hantaan and Seoul viral infections among patients with hemorrhagic fever with renal syndrome (HFRS) in Heilongjiang, China. Scand J Infect Dis 2011;43:632-641

Zhang, Y.-Z.; Zou, Y.; Fu, Z.F.; Plyusnin, A. Hantavirus Infections in Humans and Animals, China. Emerging Infectious Diseases 2010b;16:1195-1203

Zhang, Z.; Xie, X.; Chen, X.; Li, Y.; Lu, Y.; Mei, S.; Liao, Y.; Lin, H. Short-term effects of meteorological factors on hand, foot and mouth disease among children in Shenzhen, China: Non-linearity, threshold and interaction. Sci Total Environ 2016;539:576-582

Zhao, Q.; Yang, X.; Liu, H.; Hu, Y.; He, M.; Huang, B.; Yao, L.; Li, N.; Zhou, G.; Yin, Y.; Li, M.; Gong, P.; Liu, M.; Ma, J.; Ren, Z.; Wang, Q.; Xiong, W.; Fan, X.; Guo, X.; Zhang, X. Effects of climate factors on hemorrhagic fever with renal syndrome in Changchun,

2013 to 2017. Medicine (Baltimore) 2019;98:e14640

Zhu, Y.; Zhao, Y.; Zhang, J.; Geng, N.; Huang, D. Spring onion seed demand forecasting using a hybrid Holt-Winters and support vector machine model. PLoS One 2019;14:e0219889-e0219889

Zou, Y.; Xiao, Q.Y.; Dong, X.; Lv, W.; Zhang, S.P.; Li, M.H.; Plyusnin, A.; Zhang, Y.Z. Genetic analysis of hantaviruses carried by reed voles Microtus fortis in China. Virus Res 2008;137:122-128

Figure 1 The time series distribution of daily HFRS cases, meteorological and air-pollution factors in Shenyang from 2005 – 2019.

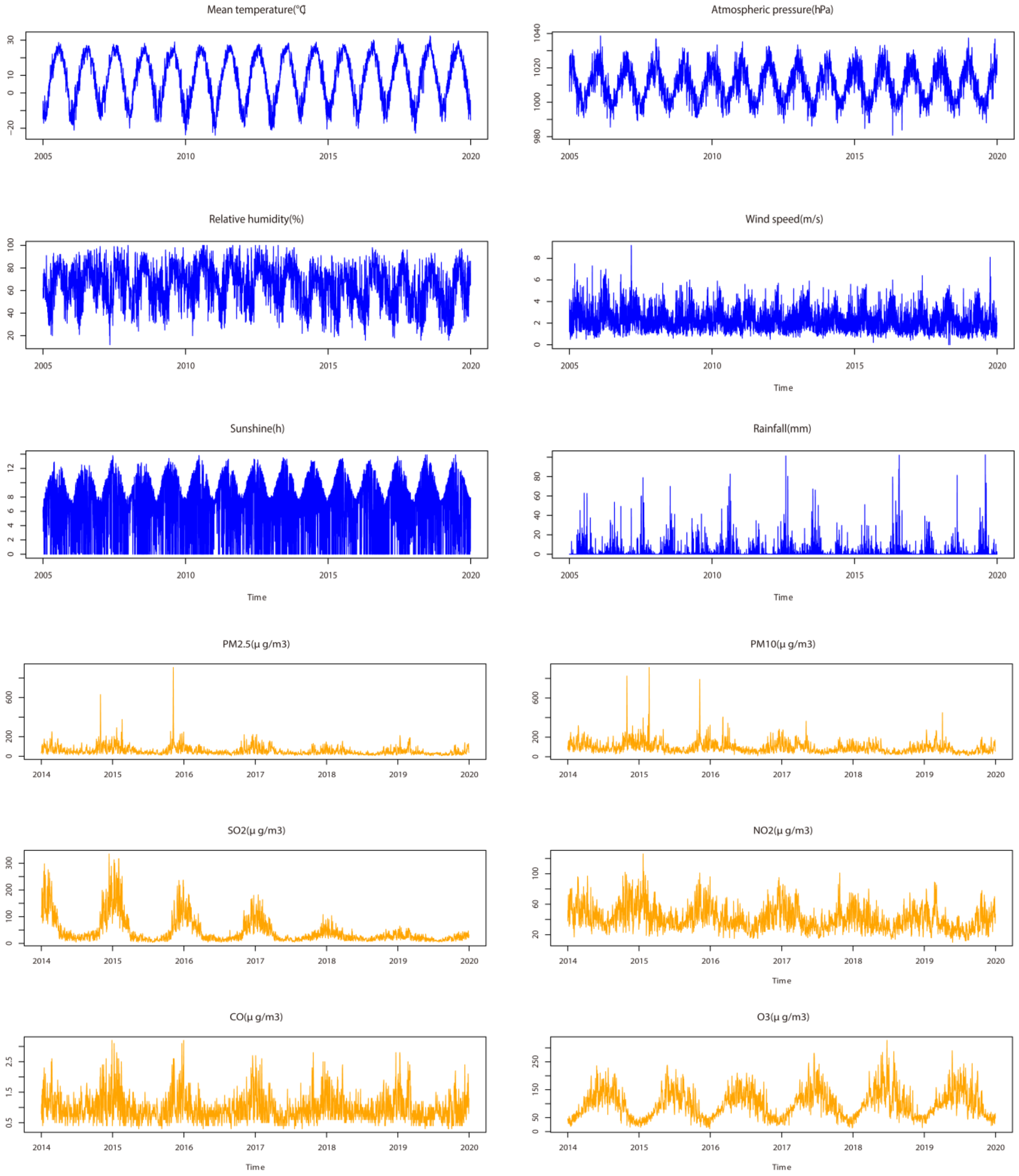

Figure 2 Resulting comparisons of the HFRS seasonal and monthly incidences using the preferred two models. The deep shaded regions indicate 80% confidence intervals , the light shaded regions indicate 95% confidence intervals.

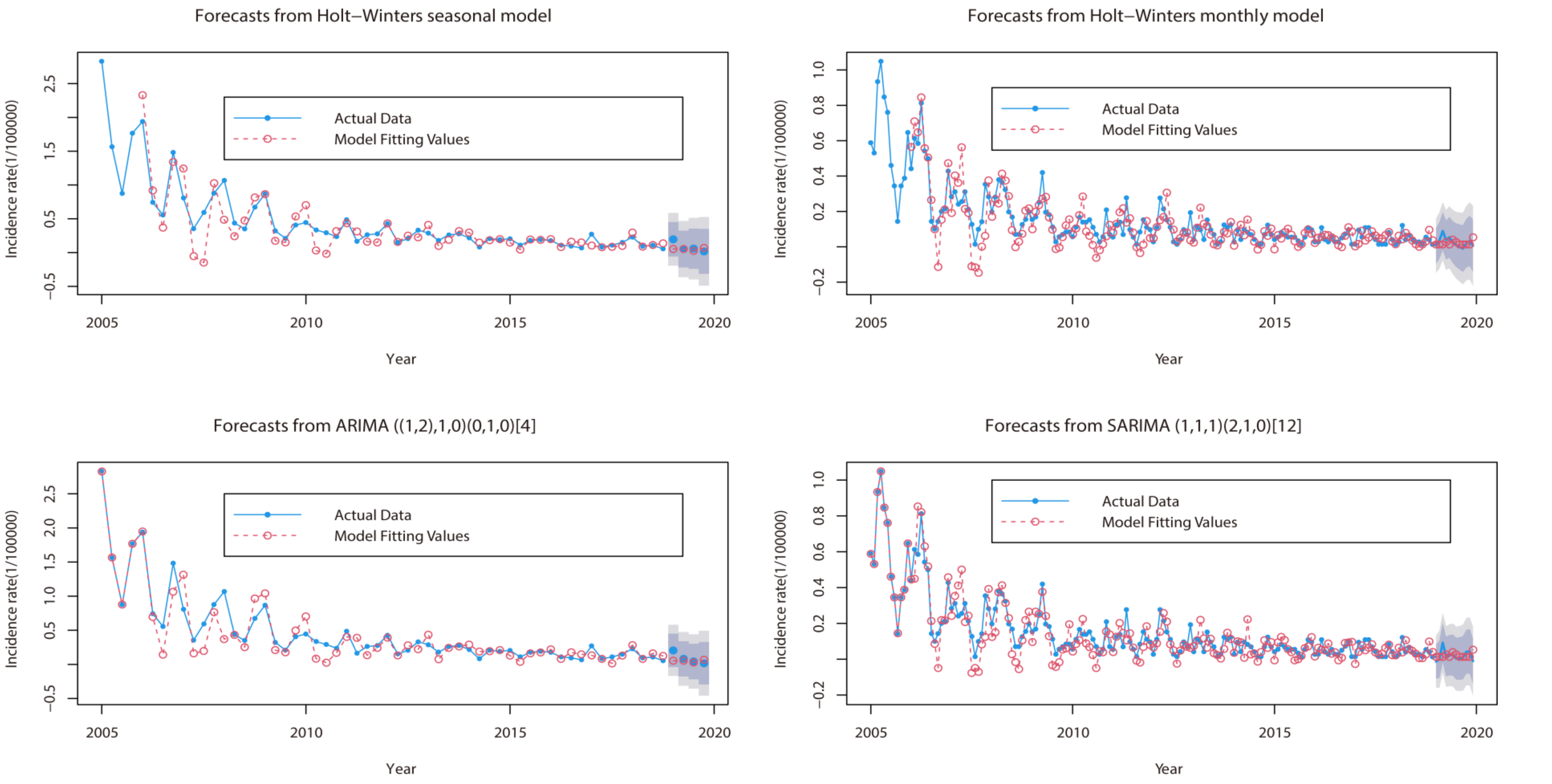

Figure 3 Summary of slices lag1-16 days exposure-response relationship between meteorological factors, air-pollution and HFRS cases for total, gender (male, female), age (20-50years, and 50-years) and delay groups (0-4days, 5-9days and 10-days) in Shenyang.

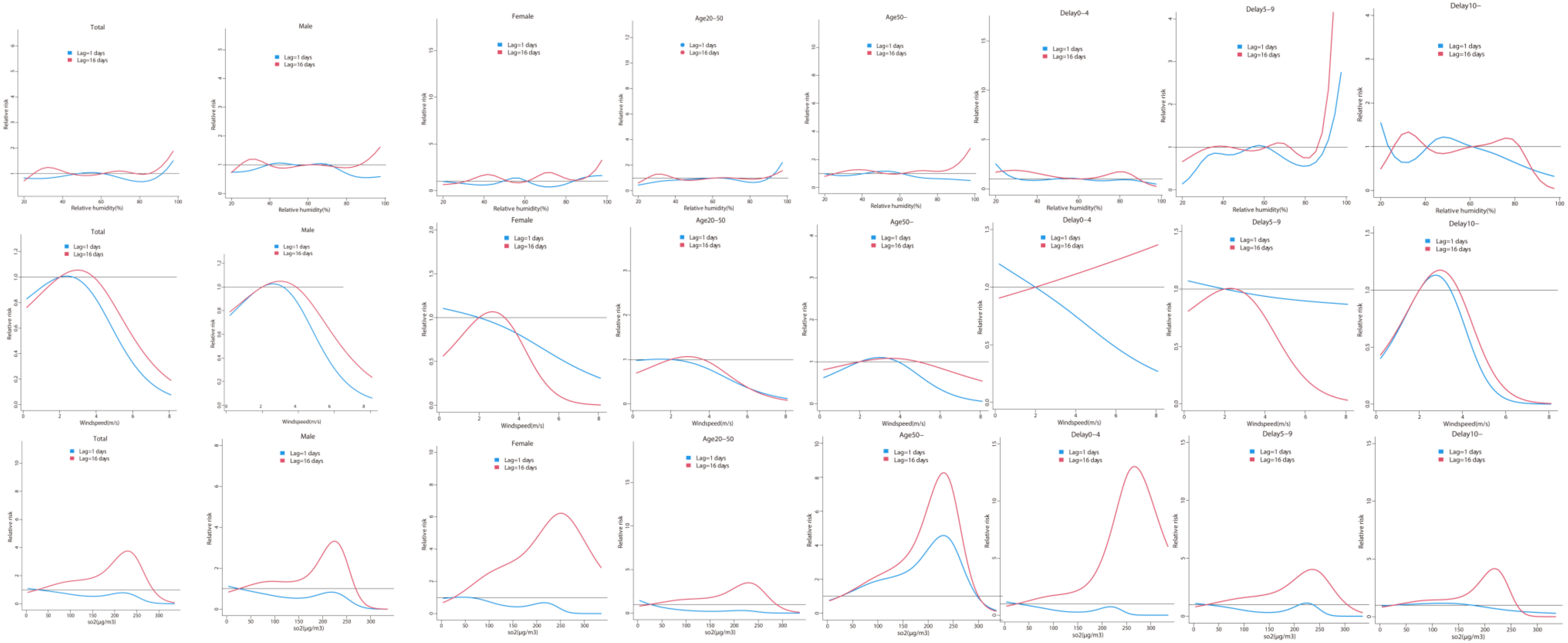

Figure 4 Summary of estimated extreme effects at the 25th and the 75th percentile of relative humidity, wind speed and SO2 on HFRS cases for total, gender (male, female), age (20-50years, and 50-years) and delay groups (0-4days, 5-9days and 10-days) at different lag days. The median value of each meteorological factor (relative humidity: 61%, wind speed: 2 m/s, SO2: 26μg/m^3) is as a reference level.

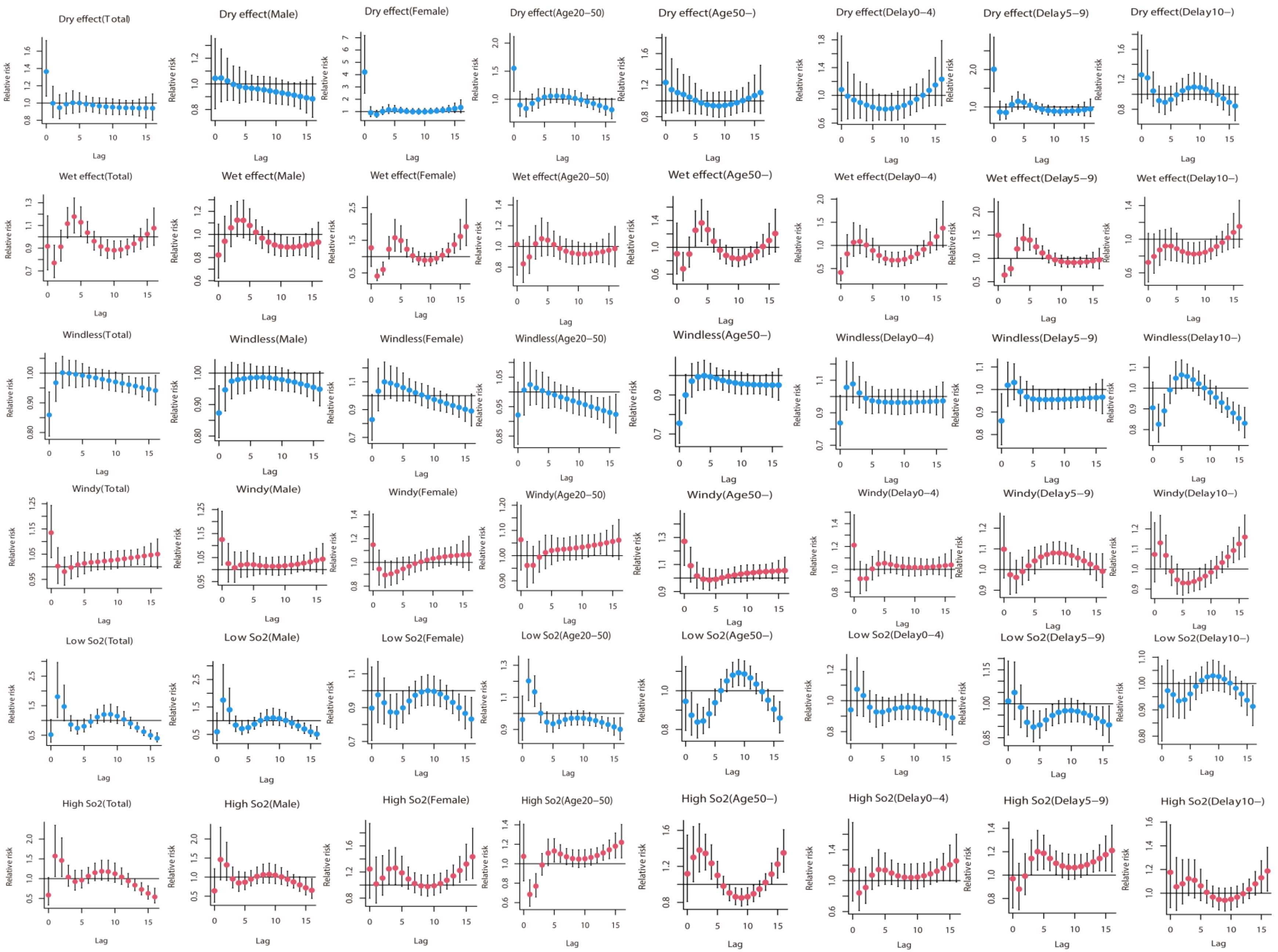
Dry effect(Total)
Dry effect(Male)
Dry effect(Female)
Dry effect(Age20–50)
Dry effect(Age50–)
Dry effect(Delay0–4)
Dry effect(Delay5–9)
Dry effect(Delay10–)
Wet effect(Total)
Wet effect(Male)
Wet effect(Female)
Wet effect(Age20–50)
Wet effect(Age50–)
Wet effect(Delay0–4)
Wet effect(Delay5–9)
Wet effect(Delay10–)
Windless(Total)
Windless(Male)
Windless(Female)
Windless(Age20–50)
Windless(Age50–)
Windless(Delay0–4)
Windless(Delay5–9)
Windless(Delay10–)
Windy(Total)
Windy(Male)
Windy(Female)
Windy(Age20–50)
Windy(Age50–)
Windy(Delay0–4)
Windy(Delay5–9)
Windy(Delay10–)
Low So2(Total)
Low So2(Male)
Low So2(Female)
Low So2(Age20–50)
Low So2(Age50–)
Low So2(Delay0–4)
Low So2(Delay5–9)
Low So2(Delay10–)
High So2(Total)
High So2(Male)
High So2(Female)
High So2(Age20–50)
High So2(Age50–)
High So2(Delay0–4)
High So2(Delay5–9)
High So2(Delay10–)
Relative risk
Lag

Figure 5 The ~~fitting~~ interactions of the association among humidity, windspeed, SO_2 and HFRS cases in Shenyang, 2014–2019 based on the growth regression tree model and the generalized additive model.

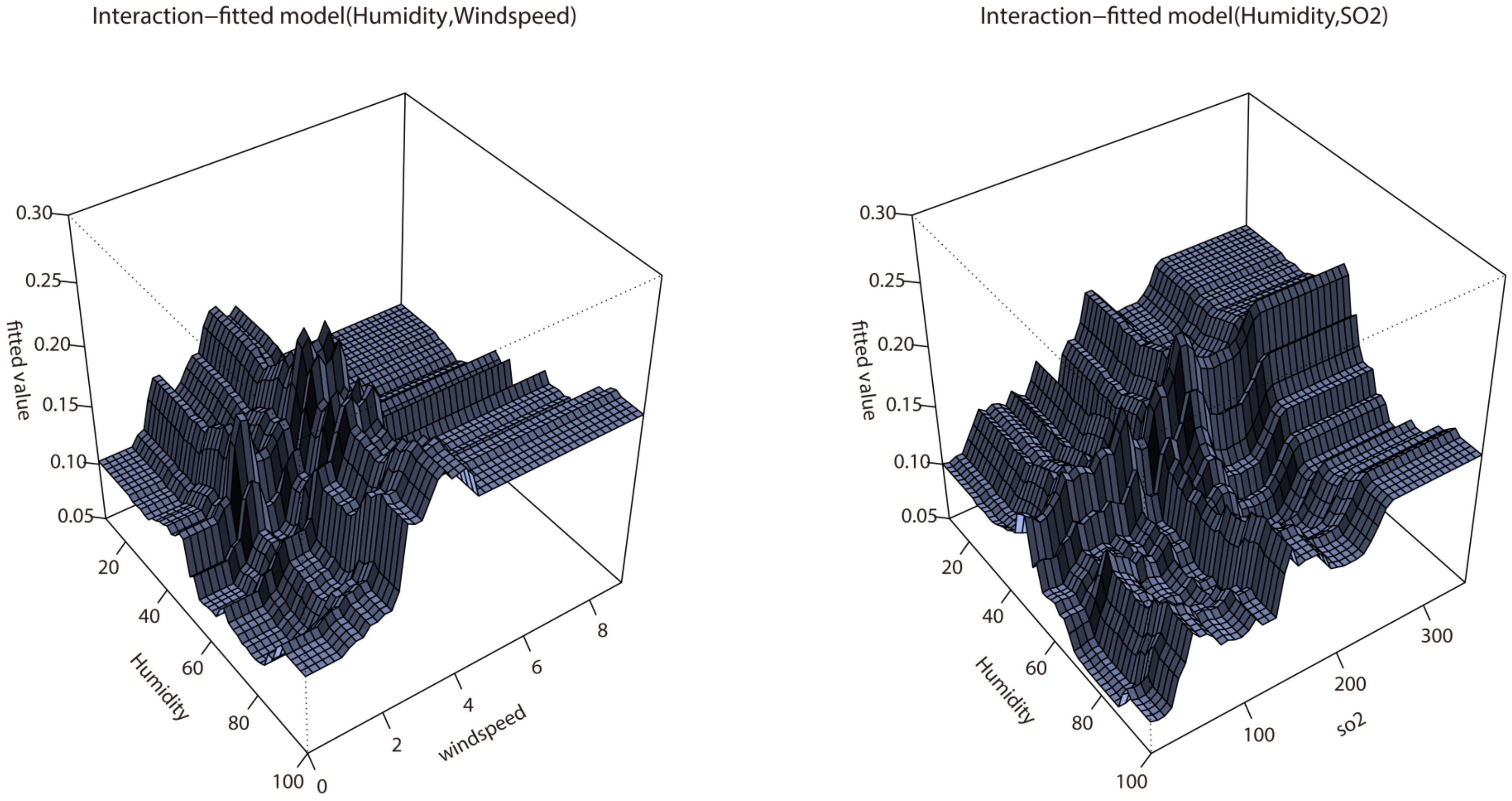

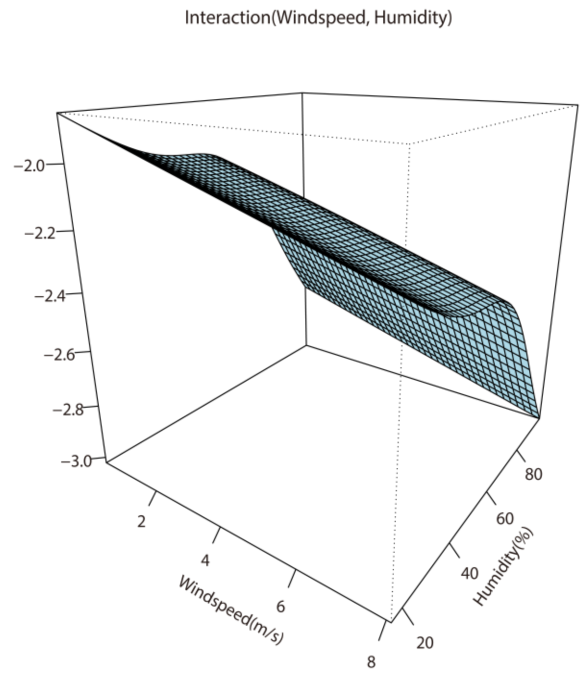
Interaction(Windspeed, Humidity)
−2.0
−2.2
−2.4
−2.6
−2.8
−3.0
2
4
6
8
Windspeed(m/s)
20
40
60
80
Humidity(%)

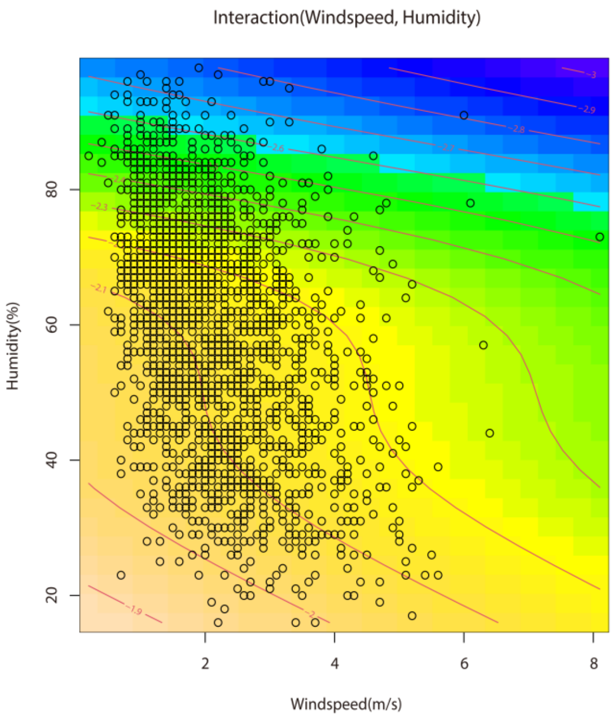
Interaction(Windspeed, Humidity)
Humidity(%)
20
40
60
80
2
4
6
8
Windspeed(m/s)

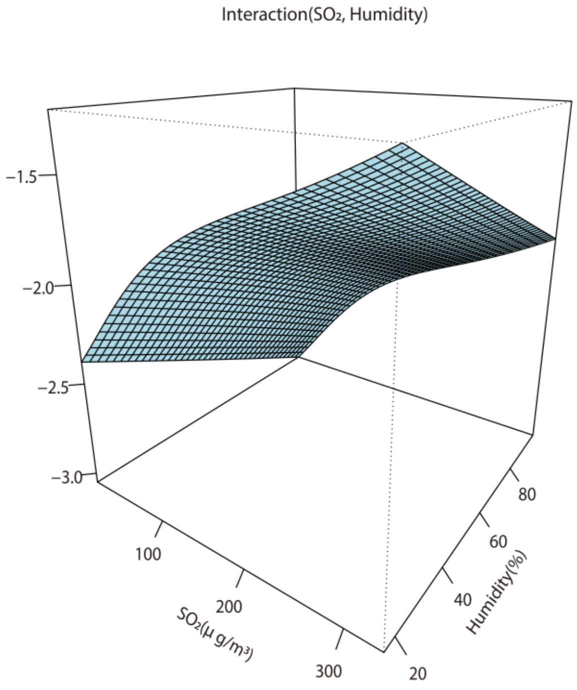
Interaction(SO₂, Humidity)
−1.5
−2.0
−2.5
−3.0
100
200
300
SO₂(μ g/m³)
20
40
60
80
Humidity(%)

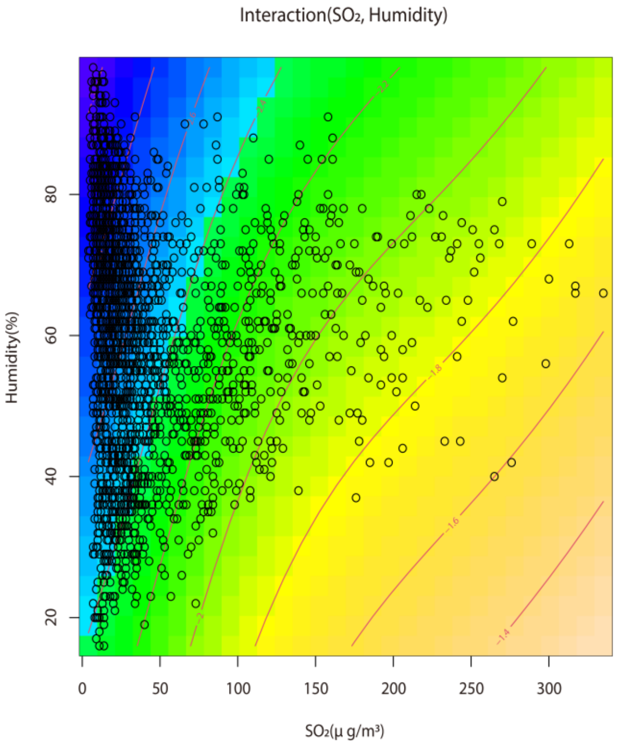
Interaction(SO₂, Humidity)
Humidity(%)
20
40
60
80
0
50
100
150
200
250
300
SO₂(μ g/m³)

Table

Click here to access/download;Table;Table.doc

Table 1 Distribution of the hemorrhagic fever with renal syndrome (HFRS) cases by age and season group in Shenyang, 2005-2019

Characteristic		0-20	20-50	＞50	Total	Population	Incidence (10^{-5}%)	No of Deaths	Mortality (10^{-5}%)	Case fatality (10^{-2}%)
		No of HFRS cases (%)								
Year	2005	31(6.33 %)	368(75.10%)	91(18.57 %)	490	6962186	7.04	4	0.057	0.816
	2006	18(5.44 %)	250(75.53%)	63(19.03 %)	331	7010640	4.72	3	0.043	0.906
	2007	11(5.91 %)	139(74.73 %)	36(19.35 %)	186	7066666	2.63	2	0.028	1.075
	2008	5(2.78 %)	139(77.22 %)	36(20%)	180	7116384	2.53	0	0.000	0.000
	2009	6(4.65 %)	88(68.22 %)	35(27.13 %)	129	7150272	1.80	1	0.014	0.775
	2010	6(6.38 %)	60(63.83 %)	28(29.79%)	94	7180769	1.31	0	0.000	0.000
	2011	5(5.81 %)	59(68.60 %)	22(25.58 %)	86	7211479	1.19	0	0.000	0.000
	2012	3(3.70 %)	59(72.84 %)	19(23.46%)	81	7237420	1.12	0	0.000	0.000
	2013	1(1.37 %)	49(67.12 %)	23(31.51 %)	73	7259528	1.01	0	0.000	0.000
	2014	0(0.%)	28(54.90 %)	23(45.10 %)	51	7289761	0.70	0	0.000	0.000
	2015	1(2%)	35(70%)	14(28%)	50	7306224	0.68	0	0.000	0.000
	2016	2(6.06 %)	25(75.76%)	6(18.18 %)	33	7324009	0.45	1	0.014	3.030
	2017	1(2.22 %)	23(51.11 %)	21(46.67 %)	45	7356745	0.61	0	0.000	0.000
	2018	3(8.33%)	21(58.33 %)	12(33.33%)	36	7414719	0.49	2	0.027	5.556
	2019	1(6.67 %)	7(46.67 %)	7(46.67 %)	15	7511923	0.20	0	0.000	0.000
Seasons	Spring(Mar-May)	14(4.53 %)	209(67.64 %)	86(27.83 %)	309	-	-	4	-	1.294
	Summer(Jun-Aug)	33(4.51 %)	543(74.18 %)	156(21.31 %)	732	-	-	3	-	0.410
	Autumn(Sep-Nov)	24(7.08 %)	223(65.78 %)	92(27.14 %)	339	-	-	2	-	0.590
	Winter(Dec-Feb)	23(4.60%)	375(75%)	102(20.40%)	500	-	-	4	-	0.800
Total		94(5%)	1350(71.81%)	436(23.19%)	1880	7511923	25.03	13	0.173	0.691

Table 2 Performance measures of time series techniques for the hemorrhagic fever with renal syndrome (HFRS) incidence in Shenyang

Model	Best parameters	Method	Box-Ljung test			RMSE	MAE	MAPE
			X-squared	Df	p-value			
Holt-Winters seasonal model	α= 0.27, β=0.38 ,γ=1	Additive	0.087	2	0.957	0.198	0.13	40.215
Holt-Winters monthly model	α= 0.24, β=0.05 , γ=0.75	Additive	7.386	6	0.287	0.078	0.054	71.535
SARIMA seasonal model	((1,2),1,0) (0,1,0) [4]	Additive	$6.59*10^{-6}$	1	0.998	0.182	0.116	38.031
SARIMA monthly model	(1,1,0) (2,1,0) [12]	Multiple	0.381	1	0.537	0.071	0.049	65.968

Table 3 The cumulative effects of extreme meteorological and air-pollution factors on HFRS cases of children by sex, age and delay

Series	Variables	Cumulative effects(95%CI)					
		Dry effect	Wet effect	Windless effect	Windy effect	Low SO_2 effect	High SO_2 effect
Sex	Total cases	0.761(0.158,3.673)	0.557(0.129,2.405)	**0.487(0.260,0.912)**	1.617(0.982,2.663)	**0.577(0.370,0.898)**	**2.583(1.145,5.827)**
	Male	0.461(0.078,2.737)	0.462(0.088,2.428)	**0.490(0.241,0.997)**	1.593(0.906,2.801)	0.692(0.425,1.126)	1.832(0.750,4.474)
	Female	9.817(0.203,473.684)	4.091(0.131,127.899)	0.683(0.153,3.039)	1.075(0.347,3.334)	**0.285(0.090,0.898)**	**8.122(1.009,65.403)**
Age	20-50 years	0.745(0.094,5.870)	0.535(0.083,3.451)	0.523(0.225,1.213)	1.544(0.787,3.032)	0.645(0.363,1.146)	2.031(0.706,5.845)
	50- years	1.574(0.097,25.407)	0.723(0.053,9.803)	**0.335(0.113,0.992)**	2.007(0.855,4.711)	0.530(0.240,1.167)	3.287(0.767,14.079)
Delay	0-4 days	0.303(0.005,19.164)	0.061(0.002,2.207)	0.589(0.129,2.677)	1.515(0.460,4.993)	0.408(0.126,1.319)	3.652(0.438,30.408)
	5-9 days	0.897(0.088,9.191)	1.540(0.175,13.525)	0.451(0.180,1.133)	1.874(0.884,3.971)	**0.427(0.213,0.858)**	**4.491(1.246,16.193)**
	10- days	1.285(0.094,17.643)	0.149(0.012,1.899)	**0.324(0.106,0.983)**	1.392(0.596,3.254)	0.638(0.314,1.297)	2.104(0.570,7.762)

Table 4 The cumulative relative risk of HFRS cases associated with extreme meteorological and air-pollution variables at various lag days during 2014-2019 in Shenyang

Variables	Relative risk (95% CI)				
	Lag0	Lag0-1	Lag0-14	Lag0-15	Lag0-16
Dry effect	1.364(0.954,1.950)	1.359(0.860,2.146)	0.860(0.215,3.444)	0.809(0.186,3.511)	0.761(0.158,3.673)
Wet effect	0.917(0.618,1.359)	0.706(0.436,1.143)	0.505(0.134,1.899)	0.518(0.130,2.060)	0.557(0.129,2.405)
Windless effect	**0.829(0.705,0.975)**	**0.795(0.642,0.986)**	**0.562(0.317,0.995)**	**0.525(0.289,0.952)**	**0.487(0.260,0.912)**
Windy effect	1.135(0.987,1.305)	1.138(0.946,1.368)	1.471(0.932,2.322)	1.539(0.959,2.471)	1.617(0.982,2.663)
Low SO_2 effect	0.960(0.815,1.131)	1.001(0.822,1.220)	0.693(0.461,1.042)	**0.640(0.420,0.975)**	**0.577(0.370,0.898)**
High SO_2 effect	1.083(0.797,1.470)	0.983(0.680,1.420)	1.814(0.860,3.828)	2.114(0.978,4.568)	**2.583(1.145,5.827)**

Click here to access/download
Supporting Information
Supplement file.doc

Figure S1 The geographical location of Shenyang City in China.

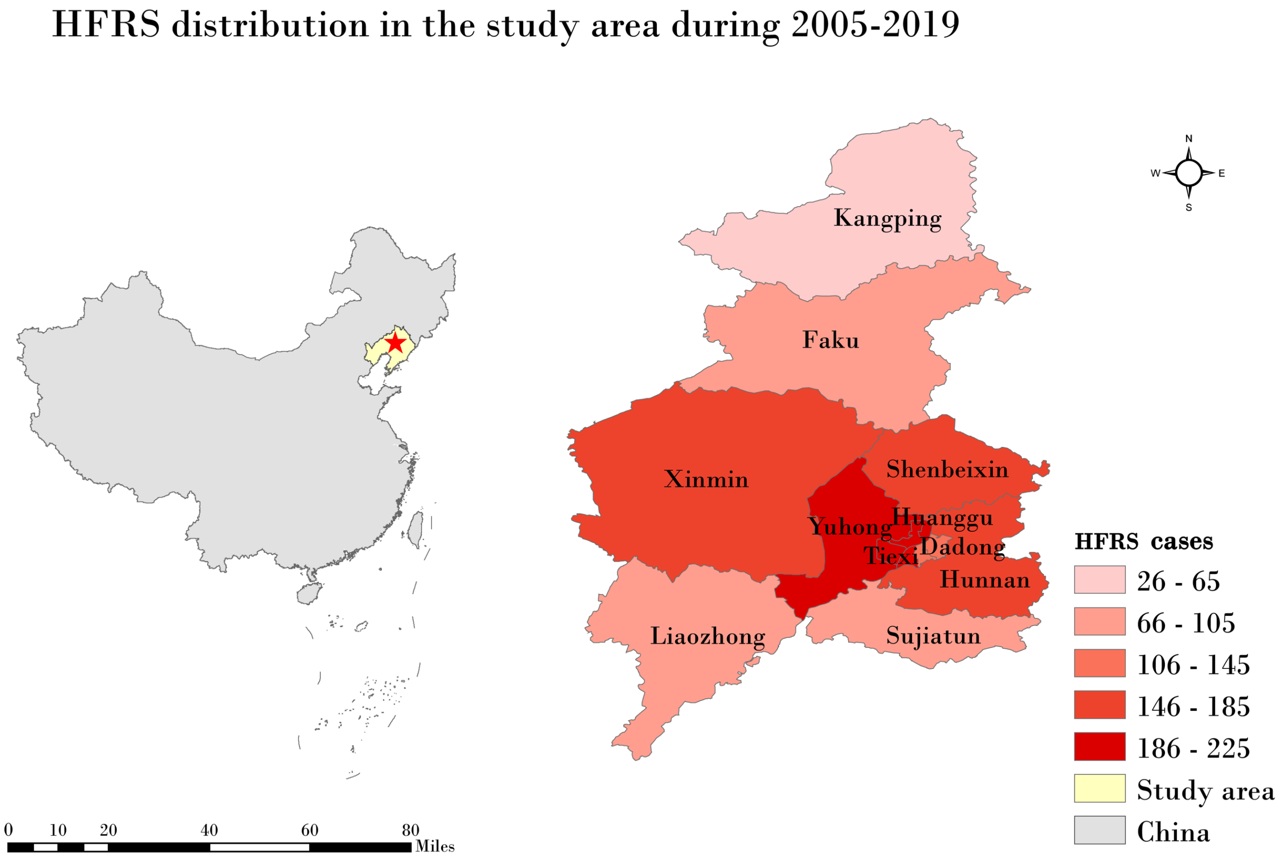

Figure S2 Decomposition of determinants in HFRS cases with seasonal and monthly model.

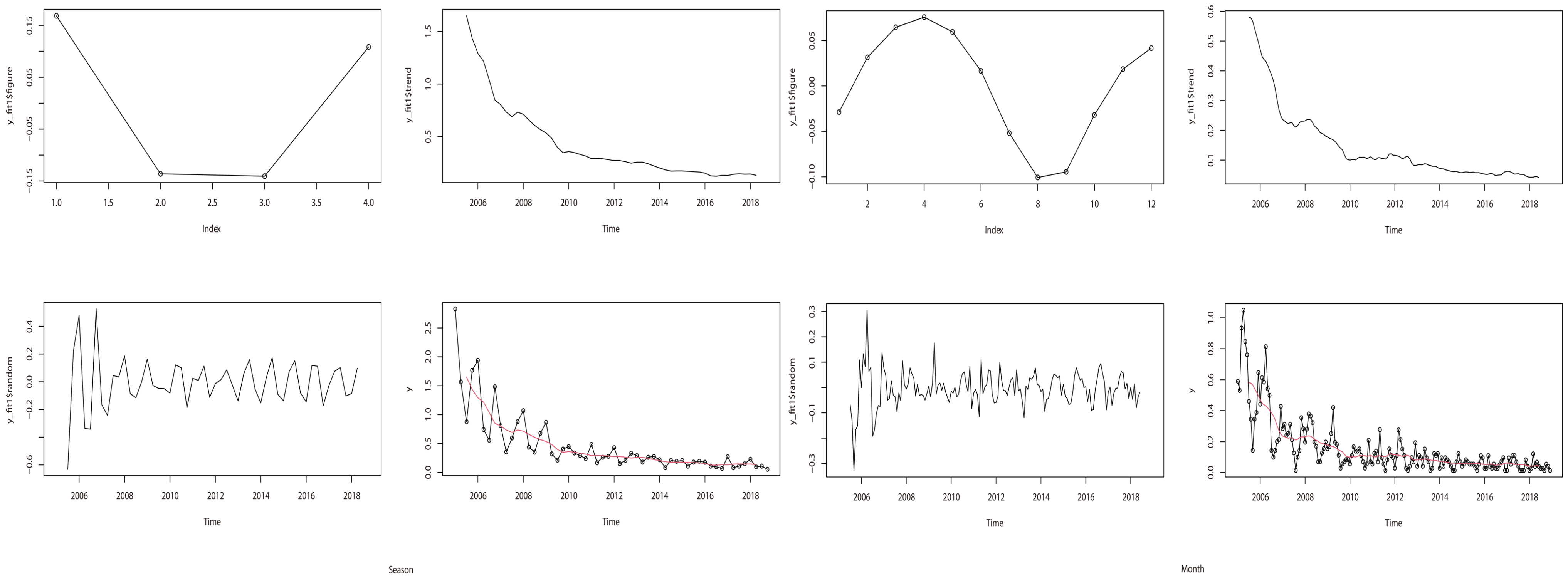

Figure S3 Autocorrelation function (ACF) and partial ACF charts of monthly HFRS incidence with SARIMA model

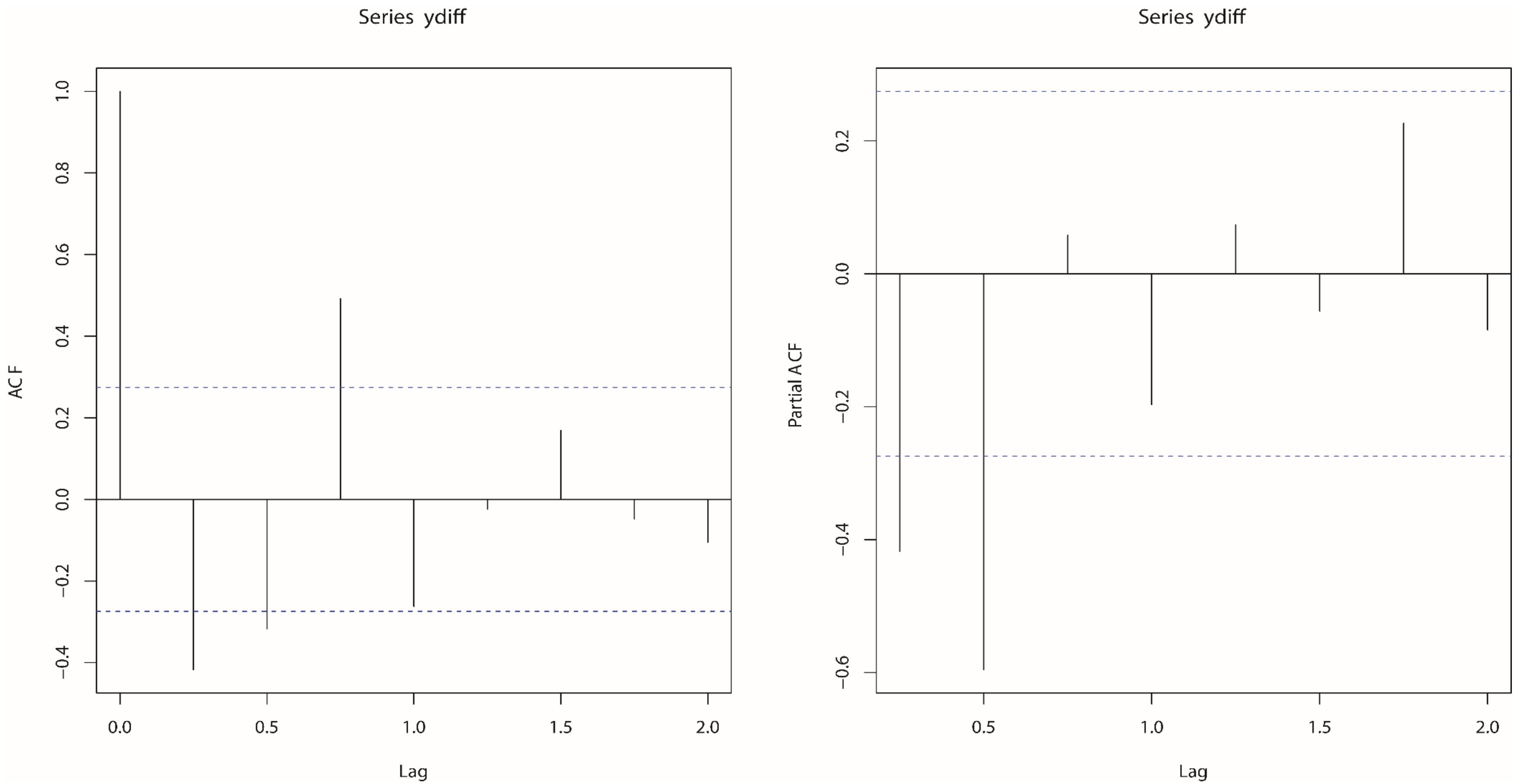

Figure S4 Autocorrelation function (ACF) and partial ACF charts of seasonal HFRS incidence with SARIMA model

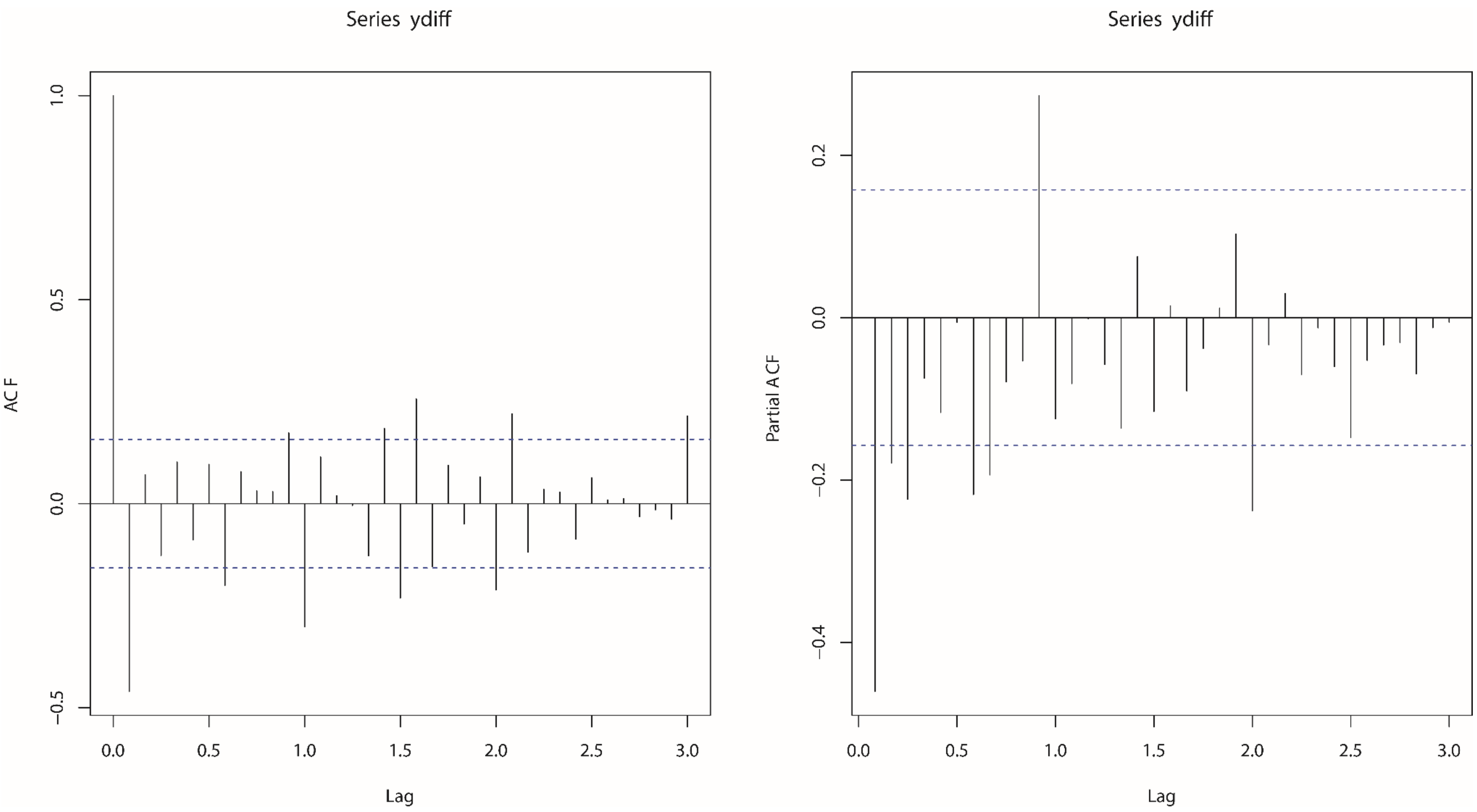

Figure S5 Tests of goodness of fit for the error series of HFRS incidence from the Holt-Winters method. (a)Autocorrelation function (ACF) plot for the seasonal Holt-Winters residual series; (A)Autocorrelation function (ACF) plot for the monthly Holt-Winters residual series; (b) Partial autocorrelation function (PACF) plot for the seasonal Holt-Winters residual series; (B) Partial autocorrelation function (PACF) plot for the monthly Holt-Winters residual series; (c) Standardized residual seasonal Holt-Winters series; (C) Standardized residual monthly Holt-Winters series. These manifested its adequacy and suitability of this data-driven hybrid model for the data.

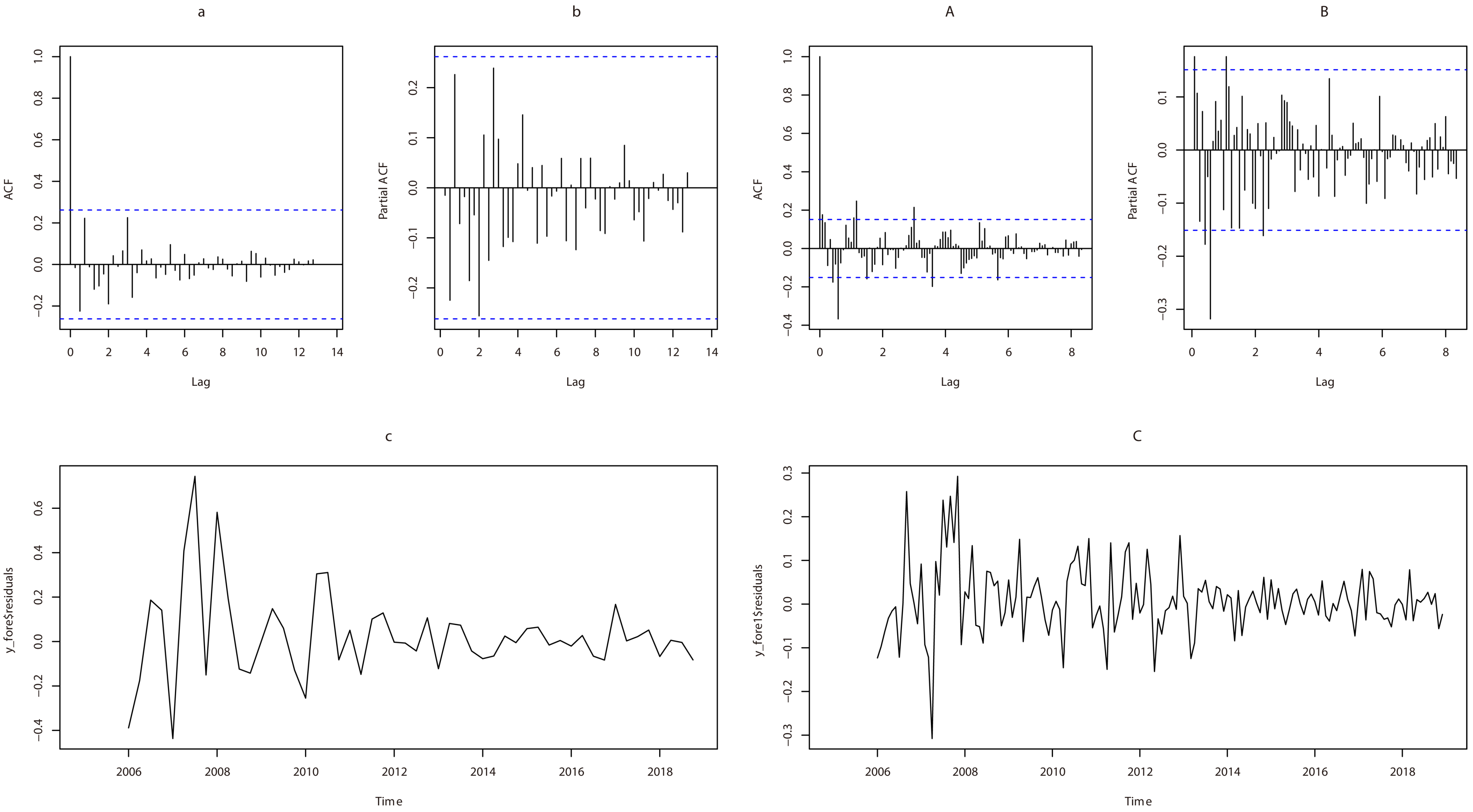
a
b
A
B
ACF
Partial ACF
ACF
Partial ACF
Lag
Lag
Lag
Lag
c
C
y_fore$residuals
y_fore1$residuals
Time
Time

Figure S6 The significance of SARIMA seasonal and monthly model

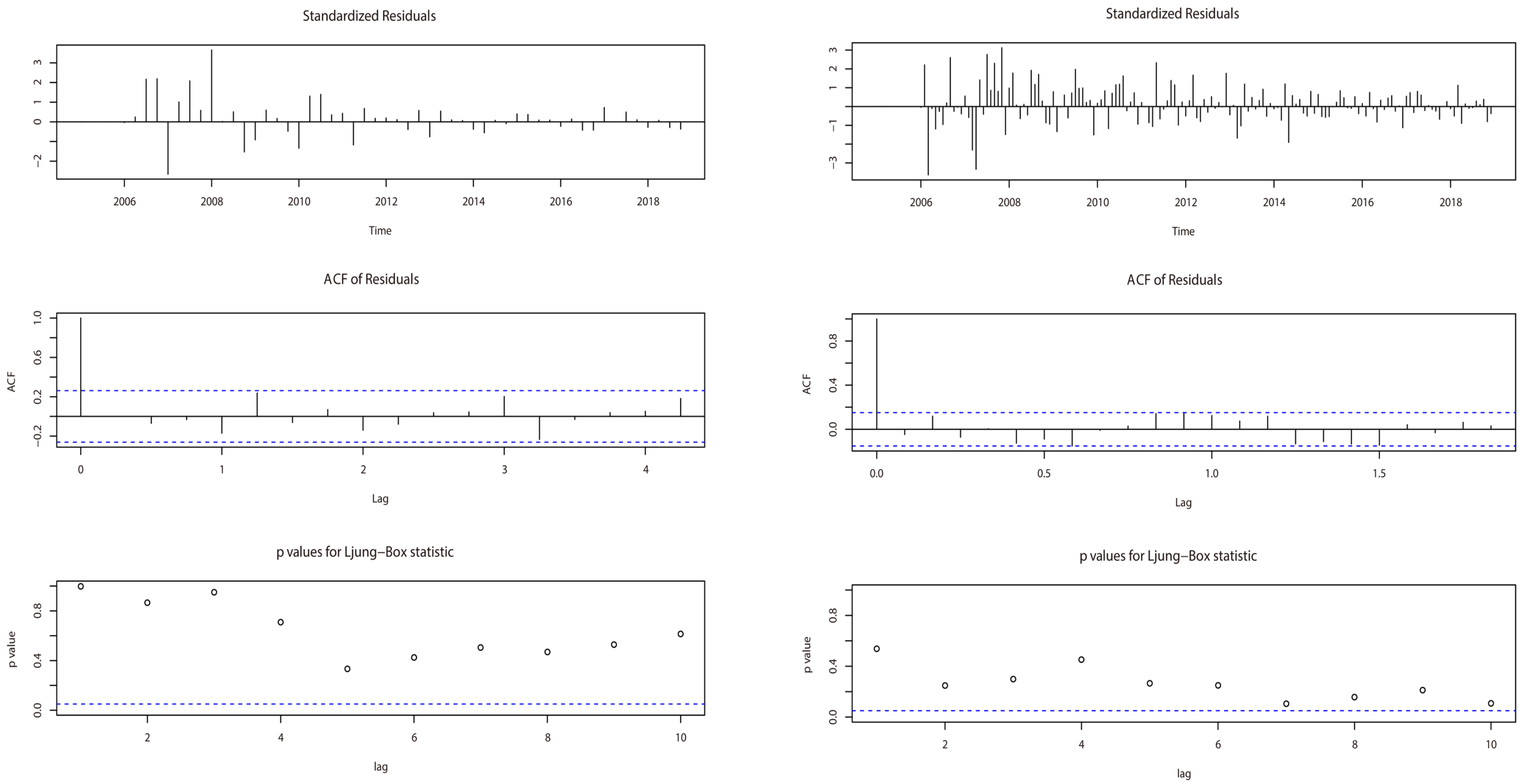

Figure S7 Fitting function variables and distribution of fitted values created based on the growth regression tree model.

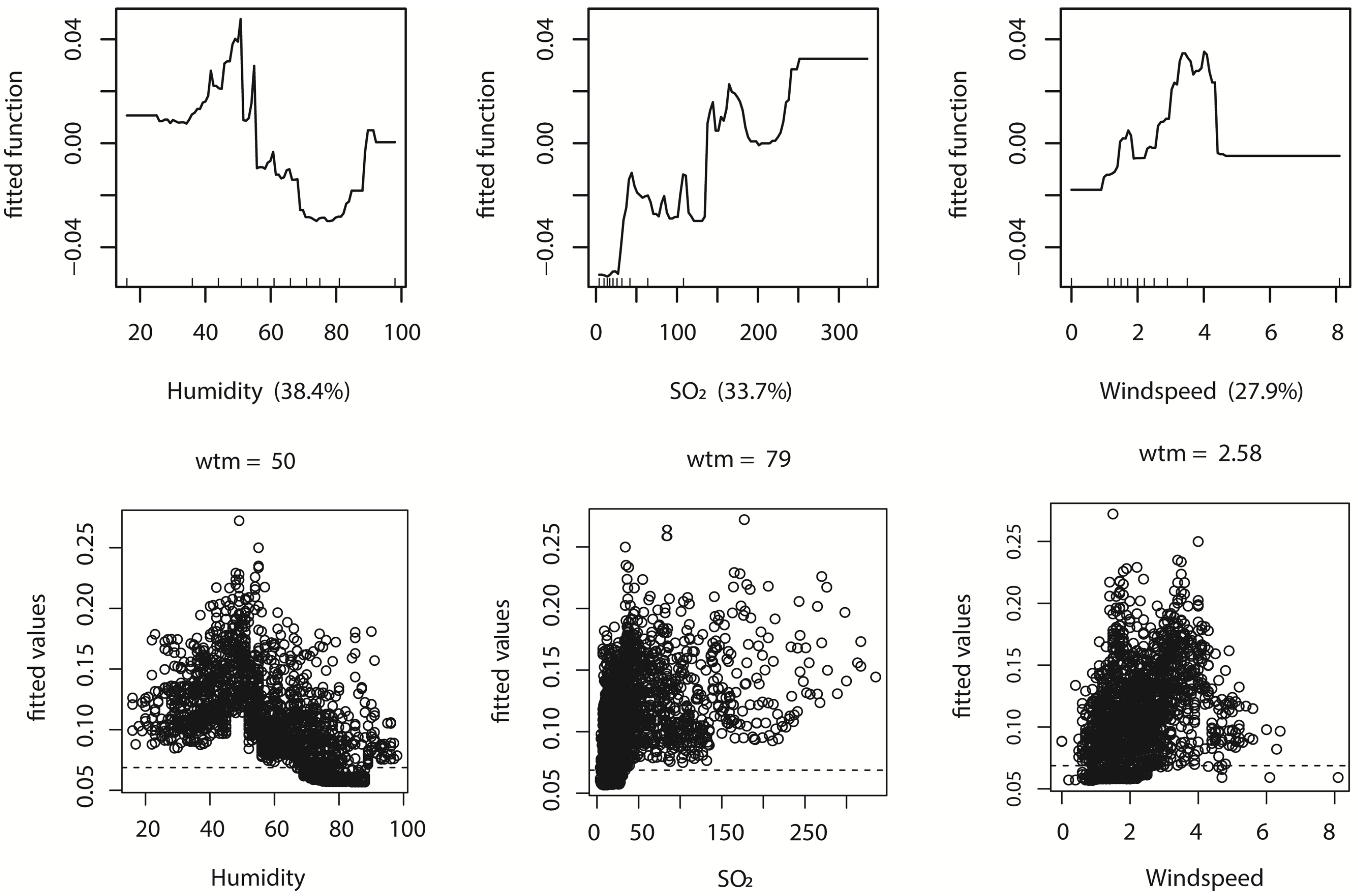

Figure S8 Relative risk of meteorological and air-pollution variables on HFRS incidence over 16 lag days, including relative humidity, wind speed and SO2.

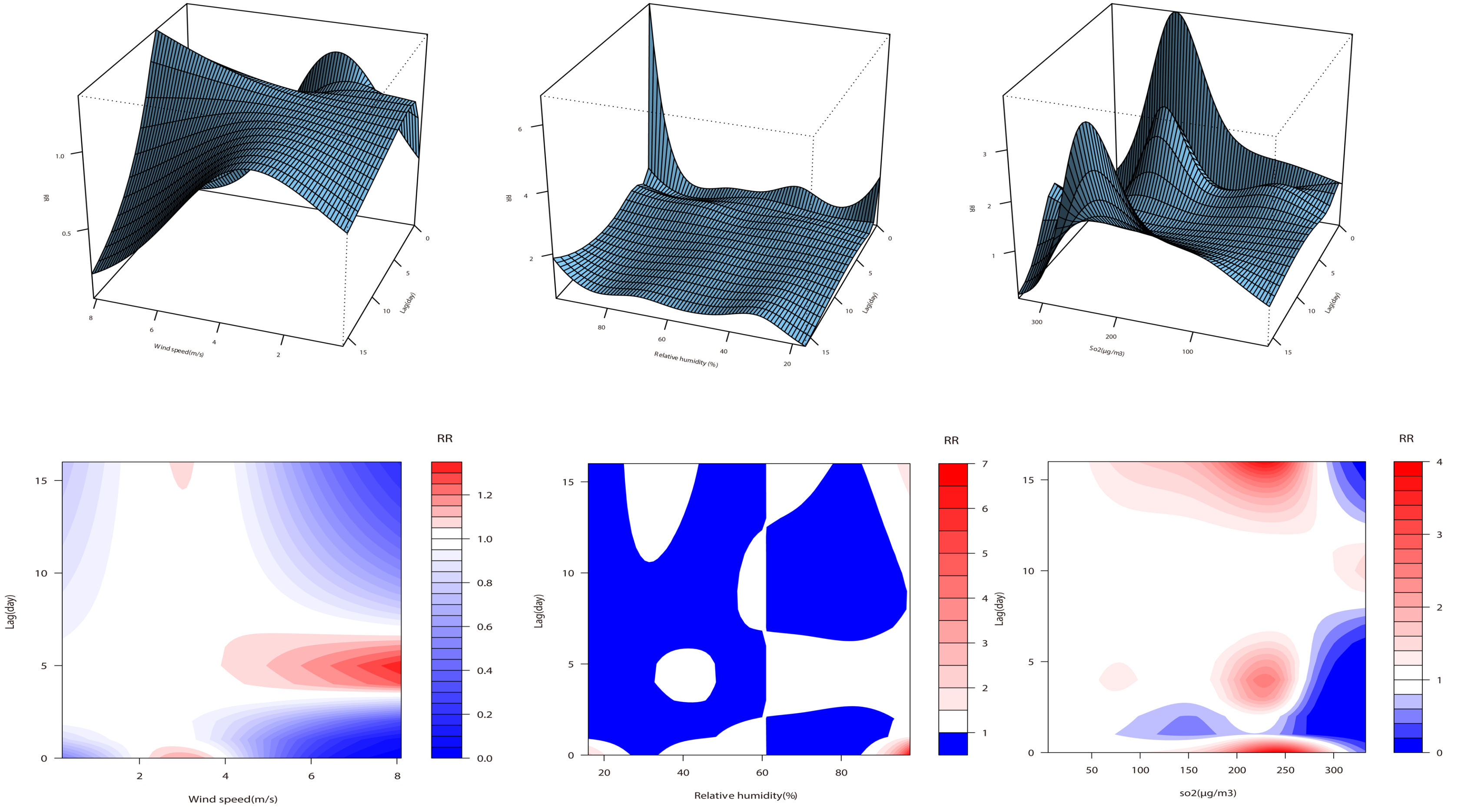

Figure S9 Effect of different meteorological and air-pollution variables on the incidence of HFRS at different days for total, gender (male, female), age (20-50 years, and 50-years) and delay groups (0-4 days, 5-9days and 10-days) in Shenyang.

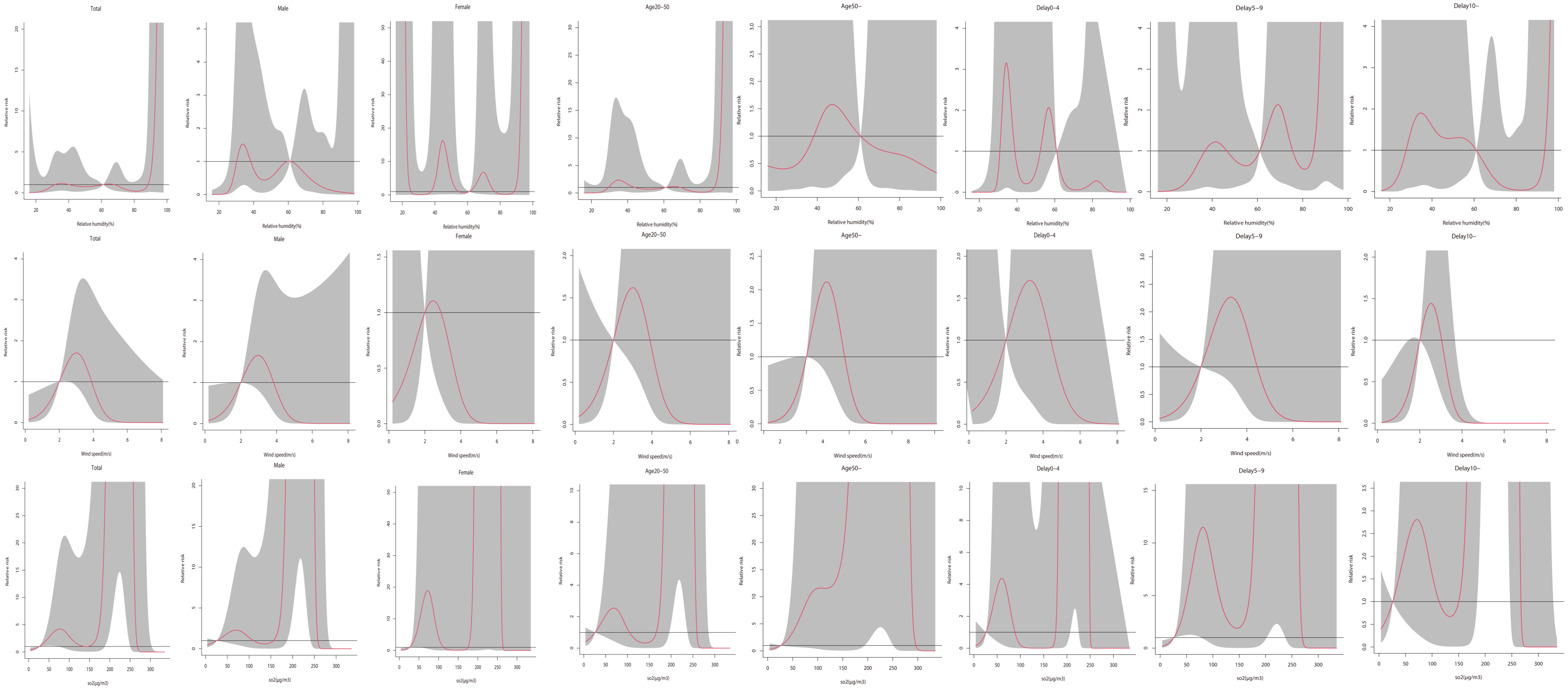

Table S1 Stratified characteristics of the hemorrhagic fever with renal syndrome (HFRS) seasonal cases of Shenyang

Characteristic		Spring	Summer	Autumn	Winter	Total	*P*-value
		No of HFRS cases (%)					
Age	0-20	14(4.53 %)	33(4.51 %)	24(7.08 %)	23(4.60%)	94	＜0.05
	20-50	209(67.64 %)	543(74.18 %)	223(65.78 %)	375(75%)	1350	
	＞50	86(27.83 %)	156(21.31 %)	92(27.14 %)	102(20.40%)	436	
The interval of days	0-4	428(38.35%)	219(19.62%)	169(15.14%)	300(26.88%)	1116	＜0.05
	5-9	248(42.18%)	96(16.33%)	94(15.99%)	150(25.51%)	588	
	10-	56(31.82%)	24(13.64%)	46(26.14%)	50(28.41%)	176	
Occupation	Farmer	210(33.49%)	135(21.53%)	109(17.38%)	173(27.59%)	627	>0.05
	Home	219(35.44%)	116(18.77%)	119(19.26%)	164(26.54%)	618	
	Other	303(47.72%)	88(13.86%)	81(12.76%)	163(25.67%)	635	

Table S2 Descriptive statistics for daily HFRS cases, meteorological and air-pollution factors in Shenyang, 2005–2019.

Series	Variables	Total	Mean±SD	Min	P25	Median	P75	Max
Daily HFRS case	Total cases	1880	0.34±0.72	0	0	0	0	7
	Male	1477	0.27±0.62	0	0	0	0	5
	Female	403	0.07±0.28	0	0	0	0	3
	Aged 1-20 years	94	0.02±0.13	0	0	0	0	2
	Aged 20-50 years	1350	0.25±0.60	0	0	0	0	5
	Aged 50- years	436	0.08±0.30	0	0	0	0	5
	Delay 0-4 days	1116	0.20±0.54	0	0	0	0	6
	Delay 5-9 days	588	0.11±0.36	0	0	0	0	4
	Delay 10- days	176	0.03±0.18	0	0	0	0	2
Meteorological parameters	Airpressure	-	1010.32±9.59	980.8	1002.4	1010	1017.8	1038.6
	Sunlight	-	6.74±3.94	0	3.7	7.7	9.8	13.9
	Meantemperature	-	8.50±13.40	-24	-3.1	10.6	20.9	32.4
	Humidity	-	64.43±16.52	12	53	66	77	100
	Wind speed	-	2.28±1.02	0	1.5	2.1	2.875	9.2
	Rainfall	-	1.87±7.04	0	0	0	0	102.4
Air pollution parameters	$PM_{2.5}$	-	55.17±46.14	4	28	42	69	908
	PM_{10}	-	94.48±61.32	9	55	80	117	912
	SO_2	-	44.15±48.32	4	15	26	49	335
	NO_2	-	41.94±16.45	10	30	39	51.5	126
	CO	-	0.98±0.45	0.3	0.7	0.9	1.2	3.2
	O_3	-	97.41±49.81	10	57	89	129	327

Table S3 Comparison of the prediction results of the two models for 2019 in Shenyang

	Time	Actual incidence	Holt-Winters	SARIMA	Holt-Winters	SARIMA	Holt-Winters	SARIMA
			Forecasted incidence		Standard Error		95%CI	
Month	January 2019	0.013	0.005	-0.013	0.007	0.027	(-0.148,0.159)	(-0.158,0.131)
	February 2019	0.013	0.035	-0.001	-0.022	0.014	(-0.124,0.193)	(-0.161,0.158)
	March 2019	0.013	0.089	0.092	-0.076	-0.079	(-0.075,0.253)	(-0.073,0.257)
	April 2019	0.027	0.038	0.015	-0.011	0.012	(-0.131,0.208)	(-0.154,0.184)
	May 2019	0.013	0.054	0.022	-0.041	-0.009	(-0.121,0.229)	(-0.150,0.195)
	June 2019	0.04	0.027	0.023	0.013	0.016	(-0.155,0.208)	(-0.152,0.200)
	July 2019	0.026	0.009	0.003	0.018	0.023	(-0.179,0.197)	(-0.175,0.182)
	August 2019	0.013	-0.0008	0.004	0.014	0.009	(-0.196,0.194)	(-0.178,0.185)
	September 2019	0.013	-0.01	0.008	0.023	0.005	(-0.213,0.192)	(-0.176,0.193)
	October 2019	0.013	0.02	0.041	-0.007	0.028	(-0.190,0.229)	(-0.146,0.229)
	November 2019	0.013	0.033	0.044	-0.02	-0.031	(-0.184,0.251)	(-0.146,0.234)
	December 2019	0.053	0.005	-0.011	0.047	0.063	(-0.220,0.231)	(-0.203,0.182)
Season	Spring 2019	0.053	0.196	0.205	-0.143	-0.152	(-0.193,0.586)	(-0.169,0.580)
	Summer 2019	0.053	0.051	0.081	0.001	-0.028	(-0.364,0.467)	(-0.313,0.475)
	Autumn 2019	0.027	0.058	0.042	-0.031	-0.015	(-0.397,0.513)	(-0.356,0.440)
	Winter 2019	0.067	0.017	0.017	0.049	0.05	(-0.490,0.525)	(-0.458,0.492)

Table S4 Spearman correlation between daily HFRS cases and the affected variables in Shenyang

	HFRS cases	Humidity	Wind speed	SO_2
HFRS cases	1.00			
Humidity	-0.10**	1.00		
Wind speed	0.07*	-0.4**	1.00	
SO_2	0.09**	-0.2**	-0.11**	1.00

** $P < 0.01$，* $P < 0.05$
