# Supplementary material for: Time series analyses based on the joint lagged effect analysis of pollution and meteorological factors of hemorrhagic fever with renal syndrome and the construction of prediction model
Source: PLoS Negl Trop Dis. 2023 Jul 24;17(7):e0010806. doi: 10.1371/journal.pntd.0010806 (PMC10399869; doi:10.1371/journal.pntd.0010806)
Supplement: Attachment — Submitted filename: Responses to Reviewers Comments(Letter).doc [file pntd.0010806.s013.doc]

**Ms. Ref. No.: PNTD-D-22-01175**

**Title:** **Time series analyses based on the joint lagged effect analysis of pollution and meteorological factors of HFRS and the construction of prediction model**

**Journal: PLOS Neglected Tropical Diseases**

**Dear Editor and the Reviewers,**

Thank you very much for your time and effort in reviewing our manuscript entitled “Time series analyses based on the joint lagged effect analysis of pollution and meteorological factors of HFRS and the construction of its prediction model”. To aid in the re-review of this manuscript, we have included a point-by-point response to each comment. The reviewers’ comments are highlighted using **bold font**. In addition, we have used "*italic text*" to denote major revisions in response to the reviewers’ comments. The changes in the revised manuscript were marked in red. We also provided a clean copy of the revised manuscript.

We appreciate the excellent suggestions and comments by the reviewers and the editor. As a consequence of the valuable suggestions, we believe that our manuscript has been improved.

**Here are my answers to the reviewers' questions as follows：**

- **1 Reviewer#1: The only ethical concern is the ethical or review board approval for access to medical surveillance data. No mention was made as to whether the data was de-identified prior to supply of data or not. It likely was but this should be made clear.**
- Thanks. Hemorrhagic fever is a Class B infectious disease under China's Infectious Disease Prevention and Control Law, and each case reported by a medical institution is reported through the direct reporting system of the infectious disease network and requires epidemiological investigation and surveillance testing to further clarify the source of the pathogen and infection. Specimens are first tested by the laboratories of medical institutions, of which positive specimens are reviewed by disease prevention and control institutions and the results are fed back to the sending units. Therefore, this study received ethical approval from the Shenyang Center for Disease Control and Prevention. For confidentiality reasons, all renal syndrome hemorrhagic fever data were analyzed anonymously.

*The present study was approved by Shenyang Center for Disease Control and Prevention (CDC). All the HFRS data were anonymously analyzed for the consideration of confidentiality.*

(Materials and methods, page 5, line 148)

- **2 Reviewer#1: Abstract - there are spaces between SO and 2 and also 12 as a subscript is missing.**
- Thanks. We have revised accordingly.
- **3 Reviewer#1: J. Roda Gracia reference should have the year in parenthesis.All references should have a space between the last word and reference.Boosted regression tree (BRT) 'verifing' should be 'verifying'. 3rd paragraph 'Zhanget al' and 'Zhaoet al' should be 'Zhang et al' and 'Zhao et al'. 4th paragraph 'Liet al. 2021' should be 'Li et al. 2021'.**
- Thanks. We have revised accordingly. We have imported a first draft that conforms to the correct literature format of the Plos journal.
- **1** **Reviewer#2: The title does not perfectly represent the statistical analysis performed in this study. The statistical analysis was partly covered for temporal analysis. I do suggest the author improved the title and correct writing of the full name of hemorrhagic fever with renal syndrome.**
- Thanks for your advice. We have revised the title.

*Time series analyses based on the joint lagged effect analysis of pollution and meteorological factors of HFRS and the construction of prediction model.*

(Title, page 1, line 1)

- **2** **Reviewer#2: It is recommended to add references to the prediction model of HFRS and analysis of related factors in 2022.**
- Thanks for the comment. We have listed some examples in regard to the prediction model of HFRS and analysis of related factors in 2022.

*Junyu He et al. applied prophet and ARIMA models in 2022 to evaluate the predictive effect of HFRS incidence in the Chinese region from 1950-2018 [15]. Yizhe Luo et al. found that temperature with a lag of 6 months (RR=3.05) and precipitation with a lag of 0 months (RR=2.08) had the greatest effect on the incidence of HFRS [23]. Also, recent Chinese studies have shown that temperature and relative humidity have an approximately parabolic or linear effect on the incidence of HFRS in 2022 [24].*

(INTRODUCTION, page 4, line 108、128)

- **3 Reviewer#2: Authors have to show ethical approve (approval No.) about patient’s specimens. Also, It is unclear by what criteria the clinical HFRS case was classified and lab diagnose was conducted (the ‘Diagnose criteria’ in ‘Methods’ section is not enough to explain). More detailed explanations were needed.**
- Thanks. We have added the diagnostic criteria for the onset of HFRS selected for this study, and ethical issues have been explained. Hemorrhagic fever is a Class B infectious disease under the Chinese Infectious Disease Control Law, and each case reported by a medical institution is reported through the direct infectious disease network reporting system and requires epidemiological investigation and surveillance testing to further define the pathogen and source of infection. Specimens are first tested by the laboratories of medical institutions, of which positive specimens are reviewed by disease prevention and control institutions and the results are fed back to the sending units. Therefore, this study received ethical approval from the Shenyang Center for Disease Control and Prevention. For confidentiality reasons, all renal syndrome hemorrhagic fever data were analyzed anonymously.

*The present study was approved by Shenyang Center for Disease Control and Prevention (CDC). All the HFRS data were anonymously analyzed for the consideration of confidentiality.*

*HFRS is divided into suspected cases, clinically diagnosed cases and confirmed cases. The diagnostic criteria for suspected cases are: a history of travel to the infected area within 2 months prior to the onset of the disease or a history of direct or indirect contact or suspected contact with rodents or their excreta (feces, urine) or secretions within 2 months prior to the onset of the disease, and does not support the diagnosis of other febrile diseases. The diagnostic cases of clinical diagnosis cases are: suspected cases with both renal damage, hypotensive shock, typical course of disease divided into febrile phase, hypotensive shock phase, oliguric phase, polyureic phase and recovery phase (five phases elapsed), blood routine examination of febrile phase with increased peripheral blood leukocyte count and thrombocytopenia, the presence of heterogeneous lymphocytes: hemoconcentration or hemodilution, urine routine: positive urine protein, blood biochemical examination: blood Creatinine, urea nitrogen elevation in at least one of the Confirmed cases: clinically diagnosed or suspected cases with positive serum specific IgM antibodies, 4-fold or higher titers of serum specific IgG antibodies in the recovery phase compared to the acute phase, detection of hantavirus RNA in patient specimens, and isolation of at least one hantavirus from patient specimens.*

(Materials and methods, page 5, line 148、163)

- **4 Reviewer#2: Please explain the division of seasons in Table S1.**
- Thanks. We have explained the following things about the division of seasons.

*From Table 2 meaningful subgroups differed in seasonal distribution, showing that different age groups were mainly concentrated in summer and winter, while groups with different days of delayed onset were mainly concentrated in spring and winter.*

(Results, page 10, line 304)

- **5 Reviewer#2:** **Can some graphic parameters in Figure S9 be further optimized?**
- Thanks. We have optimized the mapping parameters and recreated the dose-response relationship graph. (Figure S8: The order of the original Figure S9 has become Figure S8)
- **6 Reviewer#2: Result 3.4 indicate that high levels of SO2 can have a transient or continuous effect on HFRS, with the effect initially concentrated in the lag time of 0-5 days and after 15 days. The effect of high SO2 concentration on the disease is not very clear, so the conclusion that SO2 in the range of 200-250 μg/m3 increases the risk of HFRS infection and is significant in women and patients with delayed onset of 5-9 days is questionable.**
- Thanks. In this study, the lag of SO_2_ on the onset of HFRS is investigated. It can be obtained from Figures S7-S8 that the overall lag and exposure response relationship of SO_2_ showed a strong effect mainly at the level of 200-250 μg/m^3^, and the lag days were concentrated after 0-5 d and 15 d. This may be because SO_2_ may increase the chance and risk of hemorrhagic fever transmission in the aerosol pathway and influence host and population-related habits. For example, in Shenyang, a city located in northeastern China, the use of boilers for heating leads to high levels of SO_2_ in air pollution, especially in winter and spring, which resulted in severe outdoor air pollution. So, people prefer to stay indoors which leads to their exposure to rodents increased risk.
- **7 Reviewer #2: It is necessary to indicate the specific chart in which Result 3.5 is presented. It is recommended to highlight meaningful research results in Figure 3.**
- Thanks. The initial first draft did not indicate the markings of Figure 3 in result 3.5, resulting in the results of Figure 3 not being highlighted, and we apologize for this. We have labeled the results in 3.3 and 3.5 with the corresponding graphs and highlighted the focus of Figure 3 in the entire paragraph of result 3.5.
- **8 Reviewer #2:** **Does not represent at all from the result and discussion. With only three lines, i don't see it fits to be a conclusion.**
- Thanks. We have revised accordingly.

*The SARIMA model may help to enhance the forecast of monthly HFRS incidence based on a long-range dataset. Our study had shown that environmental factors such as humidity and SO_2_ have a delayed effect on the occurrence of HFRS and that the effect of humidity can be influenced by SO_2_ and wind speed. Public health professionals should take greater care in controlling HFRS in low humidity, low windy conditions and 2-3 days after SO_2_ levels above 200 μg/m^3^.*

(Author summary, page 2, line 45)

**Here are my answers to the questions posed on manuscript as follows:**

**Introduction:**

- **1: Citations through the manuscript are not properly formatted to follow PLoS NTDs guidelines.**
- We apologize for the error. We have added the Author summary ahead of the introduction.

*China has the highest number of people infected with hemorrhagic fever with renal syndrome (HFRS) in the world, and Shenyang, located in the northeast, is a high prevalence area for infection in China. Previous studies have found that there are several analytical methods on outbreak prediction and that HFRS infection is climate-related. However, HFRS has been less studied in terms of comparative time series prediction, and the link between outbreaks and atmospheric pollution and the identification of the joint effects of meteorological factors affecting this link have not been studied. These are the two main focuses of this study. A synchronous periodicity and seasonality between pollutants, climate change and HFRS infection were found throughout the study area, both located in spring-summer and winter-related. Specifically, on the one hand, high sulfur dioxide concentrations increase the risk of developing HFRS. On the other hand, the combined effect of climate and pollutants on HFRS became increasingly sensitive over time, showing as the highest risk of contracting HFRS when increasing daily sulfur dioxide and decreasing humidity. Time series analysis showed that seasonal SARIMA models are more suitable for prediction, and the association between climate and pollution and HFRS infection has been confirmed within the time series analysis. The above findings help to improve the understanding of the transmission effects of HFRS in different meteorological and pollution levels and the prediction of HFRS outbreak epidemics.*

(Author summary, page 3, line 58)

- **2: this is 46 years of data compared to only one for 2009 - how can you say this is a real or important drop based on this? and what would be the plausible explanation for such a drop in total cases, even as human populations at risk, awareness of the causative pathogens, and emergences of similar pathogens have increased?**
- We apologize for the wrong statement. We have made changes and now use the 1980 to 2009 downward trend as a description of the overall trend.

*Although HFRS cases dropped significantly from about 40,000 to 60,000 cases in the 1980s to 10,000 cases in 2009, the geographical dispersion of HFRS cases further increased, with cases notified in all 31 provinces of mainland China [4, 16].*

(INTRODUCTION, page 4, line 111)

- **3: these appear to be correlative associations, and causality should not be implied.**
- We apologize for the error. We have changed the relation of causal association between environmental factors and morbidity to a correlation.

*Some studies have found a correlation between the climatic factors and HFRS.*

(INTRODUCTION, page 4, line 121)

- **4: as above, these are not necessarily causal relationships, as the word "effects" implies. This should be changed to "associations between diverse environmental variables and HFRS." Also, the introduction needs to provide more information with references about the hypothesized causal mechanisms by which climate or other environmental conditions may affect vulnerability to HFRS.**
- Thanks for the comment. We have listed some examples in regard to the effects and mechanisms of environmental factors on the effects of HFRS.

*Yizhe Luo et al. found that temperature with a lag of 6 months (RR=3.05) and precipitation with a lag of 0 months (RR=2.08) had the greatest effect on the incidence of HFRS [23]. Also, recent Chinese studies have shown that temperature and relative humidity have an approximately parabolic or linear effect on the incidence of HFRS in 2022 [24].*

(INTRODUCTION, page 4, line 128)

- **5: The diverse set of modeling methods applied in this paper creates some confusion. Why are so many modeling approaches needed to answer the fundamental questions about the effects of environmental variables on HFRS? Why do you need ARIMA/SARIMA, BRTs, DLNMs, and GAMs? It seems that DLNMs alone could have answered all of the questions asked. This paragraph does not provide an adequate justification for the paper's flow and the unique need met by each modeling approach.**
- Thanks for the comment. In this paper, we applied SARIMA, Holt-Winters model for single-factor time series prediction of HFRS incidence, and then applied BRT model to explore its interaction. While in the past, Spearman analysis was used to explore the interaction terms. We found that wind speed and humidity, humidity and SO_2_ may have interaction. After initially fitting their model effects, DLNM model was applied to explore the lagged effects of meteorology and pollutants on HFRS. And then the GAM model was applied

to fit the previously explored interaction factors to analyze the interaction, because the generalized linear model applied by DLNM is slightly inferior to the generalized summation model in terms of interaction.

**Materials and methods:**

- **1: cite these so that readers can access the criteria**
- Thanks. We have added the diagnostic criteria for HFRS selected for this study.

*HFRS is divided into suspected cases, clinically diagnosed cases and confirmed cases. The diagnostic criteria for suspected cases are: a history of travel to the infected area within 2 months prior to the onset of the disease or a history of direct or indirect contact or suspected contact with rodents or their excreta (feces, urine) or secretions within 2 months prior to the onset of the disease, and does not support the diagnosis of other febrile diseases. The diagnostic cases of clinical diagnosis cases are: suspected cases with both renal damage, hypotensive shock, typical course of disease divided into febrile phase, hypotensive shock phase, oliguric phase, polyureic phase and recovery phase (five phases elapsed), blood routine examination of febrile phase with increased peripheral blood leukocyte count and thrombocytopenia, the presence of heterogeneous lymphocytes: hemoconcentration or hemodilution, urine routine: positive urine protein, blood biochemical examination: blood Creatinine, urea nitrogen elevation in at least one of the Confirmed cases: clinically diagnosed or suspected cases with positive serum specific IgM antibodies, 4-fold or higher titers of serum specific IgG antibodies in the recovery phase compared to the acute phase, detection of hantavirus RNA in patient specimens, and isolation of at least one hantavirus from patient specimens.*

(Materials and methods, page 5, line 163)

- **2: (**[**www.data.cma.cn**](http://www.data.cma.cn/)**) needs proper website citation in the bibliography&** **more information needed about the variables, with specific citations of the internet sources in the bibliography.**
- Thanks for your comments. We have corrected the correct website and data source and filled in the appropriate study variables.

*We obtained daily weather data for 2005 to 2019 from the China Meteorological Data Sharing Service (data.cma.cn). Meteorological factors include air pressure, sunshine, air temperature, air humidity, wind speed and rainfall. In 2013, the national air pollution population health impact monitoring project was officially launched, and Liaoning Province, one of the 16 provinces with heavy air pollution levels, also participated in the air pollution population health impact monitoring project, and Shenyang became the first monitoring city in Liaoning Province. Pollutants information for 2014 to 2019 were originally from 11 state-controlled environmental air quality automatic monitoring stations through the website of Shenyang Bureau of Ecology and Environment due to ensure the accuracy [26]. Pollutants include PM_2.5_, PM_10_, SO_2_, NO_2_, CO and O_3_.*

(Materials and methods, page 5, line 182)

- **3: needs clarification for the broad PNTDs readership, along with a citation.**
- Thanks. We agree and have added the citation in the principals of SARIMA.

*The QAIC information criterion is then used to determine the best combination of parameters for the model, and the model satisfies the residual white noise test [27].*

(Materials and methods, page 6, line 212)

- **4: are there not more seminal or comprehensive papers to cite on BRTs?**
- Thanks. We agree and have added the citation in the comprehensive analysis of interactions of BRT_S_. Since BRT has not been studied much in the medical field, especially in terms of interactions, the literature is scarce.

*Recent studies have shown that the BRT model can explain the interactions between exposures in observational studies [31].*

(Materials and methods, page 7, line 231)

- **5: meaning of simpli-categorical is unclear.**
- We apologize for the error. We have revised the technical vocabulary of the BRT principles accordingly.

*where βm is the expansion factor and b(x; γm) represent the individual trees with parameters y and variables x.*

(Materials and methods, page 7, line 236)

- **6: here and elsewhere, "wind speed" is two words and should not be capitalized.** **Chinese should be capitalized.** **minimum should be lowercase.**
- We apologize for the error. We have corrected the incorrect vocabulary and other similar errors.

*wind speed, Chinese, minimum.*

(Materials and methods, page 7, line 250-260)

- **7: sorry if I am misunderstanding, but the incubation period (lag from infection to disease onset) is not the only consideration here. What about the lag from environmental factors to changes in vulnerability of the human populations to infection?**
- Thanks. In this study, the confirmation of lag days was mainly based on the QACI criterion for sensitivity analysis, but the latency period of HFRS was also compared to verify its accuracy and to ensure that the mathematical statistics corresponded to the actual situation.
- **8: “We” correct to lowercase and clarify the meaning of this sentence.**
- We apologize for the error. We have corrected the error.

*we estimated the effect of extreme environmental factors on the incidence of HFRS by comparing the 25th or 75th percentile of environmental variables.*

(Materials and methods, page 8, line 267)

- **9: no need to capitalize variable names. did all models include an interaction? why was no model considered without an interaction?**
- We apologize for the error. We have modified the errors. In this study, the DLNM model with glm() function was first used to explore the single lag of meteorology and pollution, when there was no interaction term, and then the GAM model with gam() function was used to explore the interaction between meteorology and pollution on the onset of HFRS.

*humidity, wind speed*

(Materials and methods, page 8, line 276)

- **10: meaning is unclear, and a supplement should be included in the results to describe the approach and indicate how many values were replaced and during which time periods. Linear interpolation may not approximate true incidence, especially for extended periods with missing values. &Days? you had used days above.**
- Thanks. We have completed the missing value cases and also corrected the errors. The amount of missing data was small and mainly concentrated at the end of the year, so we applied the linear interpolation method and the results look better for predicting the missing values in this study.

*A total of 10 sets of data were missing, located in the years 2011-2019, mainly in 2016 and 2019.*

*16 days*

(Materials and methods, page 8, line 283、288)

**Results:**

- **1: Add more references to figures and tables to ensure that all results have clear indications of the evidence to support them. As written, the references to figures are sparse, with many statements made without an apparent link to a particular figure or table.**
- We thank the reviewer for the suggestion. We have added more references to figures and tables to ensure that all results have clear indications of the evidence to support them, which have been marked in red.
- **2: meaning of this is not clear, and this wording is not correct in English.** **this seems to be a period prevalence for 2005-2019? Please clearly indicate the time period for this prevalence, as the usual meaning of prevalence pertains to the fraction of individuals with a disease at a particular time point.**
- Thanks. We have revised the word.

*Men are more susceptible than women at a ratio of 3.67:1 (1477:403).* *For 2005-2019, the incidence of HFRS in Shenyang was 25.03 per 100,000, and the case fatality rate was 0.691% (Table 1).*

(Results, page 8, line 298)

- **3: This statement is unclear. This speaks of incidence rates as if they occur as singular events. Please revise for clarity.**
- Thanks for your advice. We have revised the sentences.

*HFRS in Shenyang peaked twice a year in the first and fourth quarter.*

(Results, page 11, line 313)

- **4: what does this mean? please revise for clarity.**
- Thanks. We provide an additional explanation of the methods used to model them.

*After modifying the model according to the model parameters, the incidence of HFRS from 2005-2018 was used as the training set and the training set was applied to train the established model, 2019 was used as the validation set to validate the model established by the training set.*

(Results, page 12, line 323)

- **5: what is the basis for saying this is known? If this is based on other literature and not your own findings, this should not be a part of results but could be mentioned in methods or discussion.**
- Thanks. We have revised accordingly. These were the results of this study, not a summary of previous results.

*According to the interaction fitting function in Figure 5(A, B), environmental factors fit better at moderate levels of interaction.*

(Results, page 12, line 345)

- **6: meaning? reword - I think this means at levels (not lags) of each variable that are above moderate.**
- Thanks. We have revised accordingly.

*Figure S7 shows that the non-linear exposure–lag–response association among daily humidity, wind speed, SO_2_ and HFRS incidence cases, which indicated that these factors are relatively high risk above their median levels at different lag days.*

(Results, page 12, line 348)

- **7: “varied lagging modes”unclear？**
- Thanks. We have revised accordingly.

*varied lagging features*

(Results, page 12, line 358)

- **8: these figures appear after the first references to FIg 5. please correct and check all figure references for accuracy.**
- Thanks. In this study, the figures were placed separately in the first draft and in the supplementary material according to the focus on which the results were highlighted, so there were instances where the serial numbers appeared to be incorrect. We have revised accordingly.

*Generally, analogous trends in exposure-response and lag-response relationships among environmental variables and HFRS cases across gender, age, and delay days of groups compared to total cases are shown in Figures S8, 3, and 4.*

(Results, page 13, line 372)

- **9: confusing wording - statistical significance is a result of fitting the GAM, but which term(s) are significant? The significance of an entire model has little value for inference.**
- Thanks. We have removed the word “significance”. When the GAM model was used as an interaction, significance tests were done separately for the interaction terms and confounding factors of the variables, and significant features emerged in the interaction terms. However, because of the small number of variables included in this study and the slightly inferior effect of pollutants on HFRS compared to other direct effects such as viruses in terms of the parameters of the model (R^2^, MSE, etc.), the test results were not included in this study. Therefore, the test results were not included in this study. However, it is still relevant for the interaction between pollutants and weather on HFRS.

*Statistically GAMs were built to show the interaction effect among humidity, wind speed and SO2 on HFRS incidence (Figure 5).*

(Results, page 15, line 416)

**Figures:**

- **1:** **why are some variables missing prior to 2014? All variables should be provided for the entire period.**
- Thanks. Since the region in which this study was conducted began to respond to the national call to establish monitoring stations for environmental pollutants only after 2013, we chose the pollutant data from 2014-2019 in the pollutant study to maintain accuracy. And there were also many studies that showed good validation of pollution studies in Shenyang after 2014.

*Liaoning Province, one of the 16 provinces with heavy air pollution levels, also participated in the air pollution population health impact monitoring project, and Shenyang became the first monitoring city in Liaoning Province. Pollutants information for 2014 to 2019 were originally from 11 state-controlled environmental air quality automatic monitoring stations through the website of Shenyang Bureau of Ecology and Environment due to ensure the accuracy*

(Materials and methods, page 6, line 185)

- **2:** **your model predictions are on the scale of a 1-16 days in advance, with each prediction conditional on the data up to that point in the time series. Predictions appear to be very accurate when viewed over the 15-year range, even for poor models. To make this more useful, can you provide a more objective comparison of the same model structure's predictive performance with and without the climatic variables included?**
- Thanks. In this study, the prediction was better on the single-factor time series analysis conducted without including environmental factors. While after including environmental factors, in addition to conducting a lagged study, we also performed multiple regression prediction, choosing a machine learning approach. The results were average, which it was not

as good as on the single-factor prediction on the test set. So, this study did not show the results

of multiple regression prediction after including environmental factors.

- **3:** **labels on all figures are too small and should be enlarged to make them legible.**
- Thanks. We have revised accordingly. (Figure S3)
- **4:** **clarify - are these relative risks comparing the 75th percentile value (red) and the 25th percentile value (blue) to the median for each variable? Using words like wet/dry, windy/windless, and high/low could be a bit misleading, and 25th percentile is not "windless".**
- Thanks. These are relative risks comparing the 75th percentile value (red) and the 25th percentile value (blue) to the median for each variable. We modified Windeless to Low windy and Windy to High windy. (Figure S4)
- **5:** **which plots are for which model? and positions of the horizontal axes are reversed, making comparisons more difficult. Please correct this. Also ensure that all axes are labeled (vertical axis labels are missing in the 3rd and 4th figures).**
- Thanks. We labeled the symbols, filled and reversed positions of the horizontal axes. A, B plots represent the fit of BRT to explore the interaction factors, while C,D,E,F plots represent the interaction effects among the factors analyzed by GAM. (Figure 5)
- **6:** **this should not be %? These seem to be incidence values per 100,000, not %.&should this be %? compare this to the text. I believe these are percentages, not percentages x 10^-2?&** **If this a percentage as stated in the text, correct the units above.(Table 1)**
- Thanks. These should to be incidence values per 100,000, not %. We have revised accordingly. (Table 1)
- **7:** **the value of the DLNM is in estimation of the associations over the range of lags using a smooth function. Why are these lagged effects apparently averaged to a single number? Some of the associations shown in Fig 4 apparently reverse over the period, making these cumulative effects of environmental conditions of questionable value and potentially misleading. Also, the cumulative effects presume persistence of the conditions, which may or may not be true for a given situation.**
- Thanks. Some of the associations shown in Fig 4 apparently reverse over the period. We believe that the impact on morbidity may show different effect profiles with lag days, possibly due to the instability of pollutants and meteorology before the maximum cumulative lag days occur. For example, in the high incidence age group of 20-50 years old, high levels of SO_2_ showed protective effects at short-term lag days, but reversed as a risk factor at long-term lag days. All these may be because Shenyang was at high pollution levels in winter and spring, while young people were on alert for a short period of time. But as lag days increase, so does the need for activity in young people, leading to greater exposure to external pathogens. The chance of exposure to outside pathogens will also increase, followed by a dangerous effect. However, it is true that the lag days we chose in calculating the original Table 4 were not accurate. Therefore, the suggestions raised by the reviewer were also indeed accurate and the result was indeed somewhat misleading. Bases on the reviewer's suggestion, we decided to delete the original Table 4. (Original Table 4)
- **8:** **y and x axis labels are unclear and seem to be derived from default output from statistical software. please rename to clearly define each. Change “index” to a more informative title for x axes where appropriate. Are these months?**
- Thank you for your suggestion, this image is indeed the default image run on the software, we have changed the x and y axis of the image and optimized the image, located on the left side is the split of quarterly features and the right side is the split of monthly features. (Figure S2)
- **9:** **indicate lag units - months? or years?**
- Thanks. These lag charts are quarterly and monthly. I have optimized the images and combined figures S3 and S4. Figures S3 and S4 show the ACF and PACF plots for the SARIMA model with fixed order of parameters, respectively; A and B are quarterly models and C,D are monthly models. (Figure S3)
