# Supplementary material for: Time series analyses based on the joint lagged effect analysis of pollution and meteorological factors of hemorrhagic fever with renal syndrome and the construction of prediction model
Source: PLoS Negl Trop Dis. 2023 Jul 24;17(7):e0010806. doi: 10.1371/journal.pntd.0010806 (PMC10399869; doi:10.1371/journal.pntd.0010806)
Supplement: Attachment — Submitted filename: Responses to Reviewers Comments(Letter).doc [file pntd.0010806.s014.doc]

**Ms. Ref. No.: PNTD-D-22-01175**

**Title:** **Time series analyses based on the joint lagged effect analysis of pollution and meteorological factors of hemorrhagic fever with renal syndrome and the construction of prediction model**

**Journal: PLOS Neglected Tropical Diseases**

**Dear Editor and the Reviewers,**

Thank you very much for your time and effort in reviewing our manuscript entitled “Time series analyses based on the joint lagged effect analysis of pollution and meteorological factors of hemorrhagic fever with renal syndrome and the construction of its prediction model”. To aid in the re-review of this manuscript, we have included a point-by-point response to each comment. The reviewers’ comments are highlighted using **bold font**. In addition, we have used "*italic text*" to denote major revisions in response to the reviewers’ comments. The changes in the revised manuscript were marked in red. We also provided a clean copy of the revised manuscript.

We appreciate the excellent suggestions and comments by the reviewers and the editor. As a consequence of the valuable suggestions, we believe that our manuscript has been improved.

**Here are my answers to the reviewers' questions as follows：**

- **1 Reviewer: Spell out "hemorrhagic fever with renal syndrome" as suggested in the first review. Throughout the manuscript, as pointed out in the first review, in-text citation numbers must be preceded by a space as required by PNTD guidelines. This was not corrected.**
- Thanks. We have revised accordingly.

*Time series analyses based on the joint lagged effect analysis of pollution and meteorological factors of hemorrhagic fever with renal syndrome and the construction of prediction model*

(Title, page 1, line 1-3)

- **2 Reviewer: lines 111-112: as before, this statement seems to compare cases over a whole decade (1980s) to one year. Please correct to compare annual averages. A single year does not imply a trend. Can you not point instead to the range of cases from 2000-2009, similar to the data for the 1980s?**
- Thanks. We have revised accordingly.

*The country began to show a declining trend in HFRS cases in the early 1990s and the annual number has fallen more significantly since 2000, from 37,814 cases in 2000 to 11,248 cases in 2007 [4, 16].*

(Introduction, page 4, line 110-112)

- **3 Reviewer: lines 119-120: Climatic conditions are plural. Change to "Climatic conditions are broadly regarded as some of the most pivotal factors affecting rodent population dynamics...".**
- Thanks. We have revised accordingly.

*Climatic conditions are broadly regarded as some of the most pivotal factors affecting rodent population dynamics and contributing to more cases of HFRS in humans [20].*

(Introduction, page 4, line 118-120)

- **4 Reviewer: lines 138-143: The need for so many different modeling approaches remains weakly justified, but I will accept this as written. Names of models should be plural and should not be capitalized in this paragraph (e.g., boosted regression trees, distributed lag nonlinear models, generalized additive models).**
- Thanks. We have revised accordingly.

*In this study, the incidence rate was predicted by comparison using time series analysis using HFRS surveillance data in Shenyang, followed by the application of boosted regression trees (BRT) verifying the fit and interaction among the environmental factors, and the lag effect and interaction of meteorological and pollutant factors were investigated using distributed lag nonlinear models (DLNM) and generalized additive models (GAM).*

(Introduction, page 4-5, line 139-142)

- **5 Reviewer: lines 161-163: Which Ministry of Health? Local Shenyang MoH, or all of China? Are these guidelines not published? If they are, please cite the website or other source.**
- Thanks. We have revised accordingly. All patients were diagnosed according to the Criteria and Management Principles of Renal Syndrome Hemorrhagic Fever issued by the Ministry of Health of the People's Republic of China (Ministry of Health 1998). References have been attached.

*All patients were diagnosed according to the Criteria and Management Principles of Renal Syndrome Hemorrhagic Fever issued by the Ministry of Health of the People's Republic of China* *(Ministry of Health 1998).*

(Materials and methods, page 5, line 160-162)

- **6 Reviewer: lines 168-179: This entire section needs rewording for clarity. There are problems with redundancy (e.g., "diagnostic cases of clinical diagnosis cases") and also many issues with sentence structure. Colons (:) are used, apparently to separate diagnostic criteria, and the entire section is included as if it were a single sentence (no periods are found in the 12-line section). Also, it is impossible to determine the case definition from the long list of criteria - is every condition listed required in order to be a clinically diagnosed case? The minimum requirements to be counted as a diagnosed case must be clear.**
- Thanks. We have revised accordingly.

*The number of HFRS morbidity was diagnosed according to Diagnostic Criteria and Principles of Management of Epidemic Hemorrhagic Fever (GB 15996-1995). For an HFRS case to be confirmed, the affected person must have either traveled to an endemic area or had contact with rodent feces, saliva, or urine within the two months preceding the onset of their illness. They must have experienced an acute illness characterized by abrupt onset of at least two of the following clinical features: fever, chills, hemorrhage, headache, back pain, abdominal pain, acute renal dysfunction, and hypotension. Furthermore, they must have received at least one laboratory confirmation test, such as a positive result for hantavirus-specific immunoglobulin M, a 4-fold increase in titers of hantavirus-specific immunoglobulin G, positive hantavirus-specific ribonucleic acid results by reverse transcription polymerase chain reaction in clinical specimens, or hantavirus isolated from clinical specimens, to meet the diagnostic criteria for HFRS [21, 26].*

(Materials and methods, page 5, line 162-175)

- **7 Reviewer: line 181: As stated previously, (www.data.cma.cn) needs a proper website citation in the bibliography. Websites are cited as references, not simple URLs in the text. See style #38 here: https://www.nlm.nih.gov/bsd/uniform_requirements.html**
- Thanks. We have revised accordingly.

*The weather dataset is available from* *China Meteorological Data Sharing Service System (http://data.cma.cn).*

(Materials and methods, page 5, line 178-179)

- **8 Reviewer: lines 263-264: As stated previously, the incubation period (lag from infection to disease onset) is not the only consideration here. What about the lag from environmental factors to changes in vulnerability of the human populations to infection? The response to the comment did not answer the question asked. I understand that the model considered a delay of up to 16 days based on QAIC, but why were longer lags not considered that would account for the lagged effects of the environment on vulnerability to infection and disease followed by the incubation period?**
- Thanks. In this study, the long-term lag effect of environmental factors on HFRS was investigated, so the range of the maximum lag days was chosen with partial reference to the latency period of HFRS. We applied the QAIC criterion to calculate the minimum QAIC with a maximum lag time of 16 days, and the maximum lag time is 10-20 days according to many references on the environmental lag time of HFRS. However, in this study, similar QAIC values of 17-20 d were also conducted in the pre-experiment and found to be insignificant in terms of lag effect, so the maximum lag days of 16 d was finally chosen with good results.
- **9 Reviewer: lines 268-269: Sentence remains unclear. What is meant by "comparing the 25th or 75th, percentile of environmental variables"? Did you compare the 75th to the 25th percentile? Please reword for clarity.**
- Thanks. We have revised accordingly. And to determine the effect of extreme environmental factors on the HFRS, the estimated effects were examined by comparing the 25th or 75th percentile of relative humidity, wind speed and SO_2_ with their median values. In Results, Table 4 shows the cumulative impact of the lag factor extremes on the HFRS at 16 lag days. The distributed lagged effects of extreme environmental factors at various lag days for all groups were showed in Figure 5.

*we divided environmental variables into two categories based on the 25th and 75th percentile - “low” and “high” - by their median value, in order to explore the stratified effect of modification and qualitatively study the association among environmental factors and HFRS cases.*

(Materials and methods, page 8, line 264-267)

- **10 Reviewer: lines 284-285: This clarification helps to know what was missing, but "sets of data" is still unclear. A data set can be quite large. Please specify exactly what percentage of data were missing for each of the indicated years and the length of the periods that were missing.**
- Thanks. We have revised accordingly.

*Of these, only a single month was missing in 2011, 2012, 2014, 2015, and 2017, while two months were missing in 2016 (September-October) and three months were missing in 2019 (February, July, and October).*

(Materials and methods, page 8, line 284-286)

- **11 Reviewer: line 321: The phrase "modifying the model according to the model parameters" has no clear meaning. Please reword.**
- Thanks. We have revised accordingly.

*After building a suitable model based on the model parameters, the incidence of HFRS from 2005-2018 was used as the training set and the training set was applied to train the established model, 2019 was used as the validation set to validate the model established by the training set.*

(Results, page 12, line 321)

- **12 Reviewer: lines 321-324: Previous questions were not addressed in the authors' responses: Why would you base assessments only on a single year of validation data? Using a single year for validation is relatively weak. It would be better to repeat the exercise for all years, leaving each year out as the validation data, then using all other years for training. If the authors disagree, this needs an explanation in the text.**
- Thanks. It has been shown that in the long-term forecast, for example, the forecast error increases as the number of forecast steps increases with the 3-year forecast. The long term HFRS has more variability components because infectious diseases are influenced by many factors. Therefore, this study used short-term forecasting to validate the model in a single-factor time series forecasting analysis, and Holt-Winters and SARIMA models also showed good forecasting results in several studies of short-term forecasting. (Reference: *SRFIMA model prediction for infectious diseases: application to hemorrhagic fever with renal syndrome and comparing with SARIMA*)
- **13 Reviewer: line 343: As stated in earlier input, Figure 5 is cited before Figures 3 and 4. This is out of order and must be fixed. The authors stated that they corrected this problem, but they did not fix all.**
- Thanks. We have revised accordingly.

(Results, page 12-15, line 343, 370, 373, 402, 415, 418)

- **14 Reviewer: line 414: The word "Statistically" does not add meaning to this sentence and should be deleted.**
- Thanks. We have revised accordingly.

*GAMs were built to show the interaction effect among humidity, wind speed and SO_2_ on HFRS incidence (Figure 6).*

(Results, page 15, line 414)

- **15 Reviewer: Fig 4 caption: As suggested in the earlier review, the final phrase should be corrected to "...is used as a reference level."**
- Thanks. We have revised accordingly.

*Figure 5 Summary of estimated extreme effects at the 25th and the 75th percentile of relative humidity, wind speed and SO_2_ on HFRS cases for total, gender (male, female), age (20-50years, and 50-years) and delay groups (0-4days, 5-9days and 10-days) at different lag days. The median value of each meteorological factor (relative humidity: 61%, wind speed: 2 m/s, SO_2_: 26g/m^3^) is used as a reference level.*

(Figure 5)

- **16 Reviewer: Fig 5 caption: Please correct the wording. What is a "growth regression tree model"? This term is not used elsewhere in the paper.**
- Thanks. We have revised accordingly. We adjusted Figure3-4 to Figure4-5 and modified Figure3 and 6.

*Figure 3 The fitting interactions of the association among humidity, windspeed, SO_2_ and HFRS cases in Shenyang, 2014–2019 based on the boosted regression tree models.*

*Figure 6 The effect interactions of the association among humidity, windspeed, SO_2_ and HFRS cases in Shenyang, 2014–2019 based on the generalized additive models.*

(Figure 3-6)
