# Supplementary material for: Time series analyses based on the joint lagged effect analysis of pollution and meteorological factors of hemorrhagic fever with renal syndrome and the construction of prediction model
Source: PLoS Negl Trop Dis. 2023 Jul 24;17(7):e0010806. doi: 10.1371/journal.pntd.0010806 (PMC10399869; doi:10.1371/journal.pntd.0010806)
Supplement: Attachment — Submitted filename: Revised Manuscript(Marked) editorial changes.docx [file pntd.0010806.s015.docx]

**Time series analyses based on the joint lagged effect analysis of pollution and meteorological factors of hemorrhagic fever with renal syndrome and the construction of prediction model**

Ye Chen ^1, †^, Weiming Hou ^2, †^, Jing Dong ^2,*^

^1^ Department of Infectious Disease, Shenyang Center for Disease Control and Prevention, Shenyang 110100, Liaoning Province, PR China.

^2^ Department of Occupational and Environmental Health, School of Public Health, China Medical University, No.77 Puhe Road, 110122, Shenyang, Peoples’ Republic of China.

^†^ Ye Chen and Weiming Hou contributed equally to this work.

^*^ Corresponding author: Jing Dong	Email: jdong@cmu.edu.cn

**Abstract**

***Background:***

Hemorrhagic fever with renal syndrome (HFRS) is a rodent-related zoonotic disease induced by hantavirus. Previous studies have identified the influence of meteorological factors on the onset of HFRS, but few studies have focused on the stratified analysis of the lagged effects and interactions of pollution and meteorological factors on HFRS.

***Methods:***

We collected meteorological, contaminant and epidemiological data on cases of HFRS in Shenyang from 2005-2019. A seasonal autoregressive integrated moving average (SARIMA) model was used to predict the incidence of HFRS and compared with Holt-Winters three-parameter exponential smoothing model. A distributed lag nonlinear model (DLNM) with a maximum lag period of 16 weeks was applied to assess the lag, stratification and extreme effects of pollution and meteorological factors on HFRS cases, followed by a generalized additive model (GAM) to explore the interaction of SO_2_ and two other meteorological factors on HFRS cases.

***Results:***

The SARIMA monthly model has better fit and forecasting power than its own quarterly model and the Holt-Winters model, with an optimal model of (1,1,0) (2,1,0)_12_. Overall, environmental factors including humidity, wind speed and SO_2_ were correlated with the onset of HFRS and there was a non-linear exposure-lag-response association. Extremely high SO_2_ increased the risk of HFRS incidence, with the maximum RR values: 2.583 (95%CI:1.145,5.827). Extremely low windy and low SO_2_ played a significant protective role on HFRS infection, with the minimum RR values: 0.487 (95%CI:0.260,0.912) and 0.577 (95%CI:0.370,0.898), respectively. Interaction indicated that the risk of HFRS infection reached its highest when increasing daily SO_2_ and decreasing humidity.

***Conclusions:***

The SARIMA model may help to enhance the forecast of monthly HFRS incidence based on a long-range dataset. Our study had shown that environmental factors such as humidity and SO_2_ have a delayed effect on the occurrence of HFRS and that the effect of humidity can be influenced by SO_2_ and wind speed. Public health professionals should take greater care in controlling HFRS in low humidity, low windy conditions and 2-3 days after SO_2_ levels above 200 μg/m^3^.

***Keywords:***

HFRS, Air pollution, Distribution lag nonlinear model, Generalized additive model, Interaction

**Author summary**

China has the highest number of people infected with hemorrhagic fever with renal syndrome (HFRS) in the world, and Shenyang, located in the northeast, is a high prevalence area for infection in China. Previous studies have found that there are several analytical methods on outbreak prediction and that HFRS infection is climate-related. However, HFRS has been less studied in terms of comparative time series prediction, and the link between outbreaks and atmospheric pollution and the identification of the joint effects of meteorological factors affecting this link have not been studied. These are the two main focuses of this study. A synchronous periodicity and seasonality between pollutants, climate change and HFRS infection were found throughout the study area, both located in spring-summer and winter-related. Specifically, on the one hand, high sulfur dioxide concentrations increase the risk of developing HFRS. On the other hand, the combined effect of climate and pollutants on HFRS became increasingly sensitive over time, showing as the highest risk of contracting HFRS when increasing daily sulfur dioxide and decreasing humidity. Time series analysis showed that seasonal SARIMA models are more suitable for prediction, and the association between climate and pollution and HFRS infection has been confirmed within the time series analysis. The above findings help to improve the understanding of the transmission effects of HFRS in different meteorological and pollution levels and the prediction of HFRS outbreak epidemics.

**1.****Introduction**

This hantavirus-like infection has attracted world attention during the Korean War since it was first described in Chinese texts 900 years ago [1]. Two clinical syndromes caused by hantavirus infection have been characterized: hantavirus cardiopulmonary syndrome(HCPS), prevalent mainly in America, and Hemorrhagic fever with renal syndrome(HFRS), found in Eurasia [2]. HFRS [1], characterized by headache, fever, back pain, abdominal pain and acute renal insufficiency [3], has caused a variety of public problems, with 30,000-60,000 cases per year in mainland China in the 1990s [4]. In Europe, over 9000 cases of HFRS are reported annually, and most cases associated with HFRS are diagnosed in parts of Europe in Russia, Finland and Sweden [5, 6]. China has the highest rate of HFRS disease in the world, with domestic HFRS cases constituting 90% of the total number of cases worldwide each year [7, 8]. HFRS is a zoonotic disease associated with rodents and a legally reported disease in China [9].

In the past, descriptive statistical methods were mostly used for the analysis of infectious diseases such as HFRS. Nonetheless, effective prediction of short and medium term HFRS incidence can provide a reliable basis for the Center of Disease Control (CDC), as well as scientific theory and support for national infectious disease prevention and control planning [10]. With the application of big data prediction technology in various fields, it also supplies approaches for the development of HFRS prediction technology. At present, there are many ways to study data prediction technology at home and abroad, and they have been broadly applied good results [11]. Regarding infectious disease forecasting, autoregressive integrated moving average (ARIMA) and Holt-Winters models are one of the most representative and widely used models in time series forecasting [12]. Today, predictive modeling has been used in many studies in epidemiological research. Some researchers applied the seasonal autoregressive fractionally integrated moving average (SARFIMA) model to predict renal syndrome hemorrhagic fever [13]. Furthermore, a European study had applied mathematical modeling to explore the pathogenesis and impact of influenza and pathogens [14]. Junyu He et al. applied prophet and ARIMA models in 2022 to evaluate the predictive effect of HFRS incidence in the Chinese region from 1950-2018 [15].

China began to show a declining trend in HFRS cases in the early 1990s and the annual number has fallen more significantly since 2000, from 37,814 cases in 2000 to 11,248 cases in 2007 [4, 16]. Seven hantavirus serotypes/genotypes have been identified in China [17]. Of these, Hantavirus and Seoul virus are the main pathogens of HFRS, and cases caused by Hantavirus account for about 70% of domestic HFRS cases [18]. Climate and environmental changes might impact the reservoir ecology and dynamics of rodent carriers, thereby triggering the spread of hantavirus transmission [4, 19].

Climatic conditions are broadly regarded as some of the most pivotal factors affecting rodent population dynamics and contributing to more cases of HFRS in humans [20]. Some studies have found a correlation between the climatic factors and HFRS. For instance, a systematic evaluation of climate variability and human hantavirus infection in Europe was previously carried out by J. Roda Gracia et al. In 2010, some researches in China used time-series Poisson regression model to examine the independent effect of climate variables on the spread of HFRS, pointing out the important role of climate variation in the transmission of HFRS in northeastern China [21].It will contribute to future international discussions on zoonotic diseases in the context of climate change [22]. Yizhe Luo et al. found that temperature with a lag of 6 months (RR=3.05) and precipitation with a lag of 0 months (RR=2.08) had the greatest effect on the incidence of HFRS [23]. Also, recent Chinese studies have shown that temperature and relative humidity have an approximately parabolic or linear effect on the incidence of HFRS in 2022 [24]. Yet little research has been done on the correlation between pollutants and HFRS epidemics. And few studies have synthesized the lagged effects of diverse environmental variables on the onset of HFRS and analyzed the interactions among them. Therefore, we explored the relationship between meteorological and pollutant factors and the onset of HFRS, and speculated that the interaction among environmental factors is of attention for HFRS.

In this study, the incidence rate was predicted by comparison using time series analysis using HFRS surveillance data in Shenyang, followed by the application of boosted regression trees (BRT) verifying the fit and interaction among the environmental factors, and the lag effect and interaction of meteorological and pollutant factors were investigated using distributed lag nonlinear models (DLNM) and generalized additive models (GAM).

**2.****Materials and methods**

**2.1 Data collection**

***Ethics statement.***

The present study was approved by Shenyang Center for Disease Control and Prevention (CDC). All the HFRS data were anonymously analyzed for the consideration of confidentiality.

***Setting.***

Shenyang is the capital city of Liaoning Province. It is a district-level city in China, covering both urban and rural locations. Shenyang is located in latitude 41°11′–43°02′N and longitude 122°25′–123°48′E, measures 12,860 Sq km and composed of 13 districts and 214 towns [25]. In 2019, Shenyang City’s average population was 7,511,923. Shenyang city belongs to the temperate semi-humid continental climate zone. The geographical situation of Shenyang is indicated in Figure S1.

***The HFRS dataset.***

We obtained surveillance data on cases of HFRS in Shenyang between 2005 and 2019 from CDC of Shenyang. All patients were diagnosed according to the Criteria and Management Principles of Renal Syndrome Hemorrhagic Fever issued by the Ministry of Health of the People's Republic of China (Ministry of Health 1998). The number of HFRS morbidity was diagnosed according to *Diagnostic Criteria and Principles of Management of Epidemic Hemorrhagic Fever* (GB 15996-1995). For an HFRS case to be confirmed, the affected person must have either traveled to an endemic area or had contact with rodent feces, saliva, or urine within the two months preceding the onset of their illness. They must have experienced an acute illness characterized by abrupt onset of at least two of the following clinical features: fever, chills, hemorrhage, headache, back pain, abdominal pain, acute renal dysfunction, and hypotension. Furthermore, they must have received at least one laboratory confirmation test, such as a positive result for hantavirus-specific immunoglobulin M, a four-fold increase in titers of hantavirus-specific immunoglobulin G between acute and convalescent serum samples, positive hantavirus-specific ribonucleic acid results by reverse transcription polymerase chain reaction in clinical specimens, or hantavirus isolated from clinical specimens, to meet the diagnostic criteria for HFRS [21, 26].

***Pollution and meteorological factors data.***

We obtained daily weather data for 2005 to 2019 from the China Meteorological Data Sharing Service. The weather dataset is available from China Meteorological Data Sharing Service System (http://data.cma.cn). Meteorological factors include air pressure, sunshine, air temperature, air humidity, wind speed and rainfall. In 2013, the national air pollution population health impact monitoring project was officially launched, and Liaoning Province, one of the 16 provinces with heavy air pollution levels, also participated in the air pollution population health impact monitoring project, and Shenyang became the first monitoring city in Liaoning Province. Pollutants information for 2014 to 2019 were originally from 11 state-controlled environmental air quality automatic monitoring stations through the website of Shenyang Bureau of Ecology and Environment due to ensure the accuracy [27]. Pollutants include PM_2.5_, PM_10_, SO_2_, NO_2_, CO and O_3_.

**2.2 SARIMA and Holt-Winters model construction**

Based on the quarterly and monthly data series, additive and multiplicative models were adopted to build factor decomposition models respectively, followed by the application of simple central moving average method to decompose the following four factor maps respectively: (1) long-term trend. (2) cyclical fluctuations. (3) seasonal variations. (4) random fluctuations. Auto regressive integrated moving average model [10] is a time series forecasting method proposed by Geogre Box and Gwilym Jenkins. The ARIMA model is a classical time-series analysis method and is extensively used. The SARIMA model is developed on the foundation of the ARIMA model. The SARIMA model is based on a further development of the ARIMA model, which is particularly suitable for cases where both trend and seasonality are present in the series. The SARIMA model is abbreviated as SARIMA (P, D, Q) _S_, where p and q are the orders of autoregressive and moving average, P and Q are the orders of seasonal autoregressive and moving average, d is the number of variances, D is the number of seasonal variances, and S is the seasonal period and cycle length [9]. The construction of the SARIMA model is shown as following equation:

$$\begin{matrix} \Phi(L)A_{P}\left(L^{s}\right)\left(\nabla^{d}\nabla_{s}^{D}x_{i}\right)=\Theta_{q}(L)B_{q}\left(L^{s}\right)\varepsilon_{t}, \\ E\left(\varepsilon_{t}\right)=0,\mathrm{Var}\left(\varepsilon_{t}\right)=\sigma_{s}^{2},E\left(\varepsilon_{t}\text{∣}\varepsilon_{s}\right)=0,s\neq t, \\ E\left(x_{s}\varepsilon_{t}\right)=0,s<t \end{matrix}$$

where L is the delay operator, *A_P_*(*L^s^*) is the p-order autoregressive operator, *A_q_*(*L^s^*) is the q-order seasonal moving average operator, ∇*^d^*=(1-*L*)*^d^* is the difference operation, and ∇*D s*=(1-*L^s^*)*^d^* is the seasonal difference operation. The order of approximation of the model is determined based on the autocorrelation function. The QAIC information criterion is then used to determine the best combination of parameters for the model, and the model satisfies the residual white noise test [28].

The Holt-Winters model is a forecasting technique proposed by Holt and Winters in 1960 that is based on speculative smoothing. Unlike ARIMA, Holt's linear equation has a built-in equation for seasonal factors that directly captures seasonality [29].

Three smoothing equations are used to calculate and evaluate deseasonalized series, trends and seasonality variables. The Holt–Winters’ additive method can be written as follows:

$$L_{t}=\alpha\left(y_{t}-S_{t-s}\right)+(1-\alpha)\left(L_{t-1}+b_{t-1}\right)$$

$S_{t}=\delta\left(y_{t}-L_{t}\right)+(1-\delta)S_{t-1}$ $b_{t}=\gamma\left(L_{t}-L_{t-1}\right)+(1-\gamma)b_{t-1}$

$$S_{t}=\delta\left(y_{t}-L_{t}\right)+(1-\delta)S_{t-1}$$

where *t* =1, ..., n, *S* represents the length of seasonality (months), *L_t_* represents the level of the series, and *b_t_* denotes the trend and seasonal components. The constants used in this model are *α* (horizontal smoothing constant), *γ* (trend smoothing constant) and *δ* (seasonal smoothing constant) [30].

**2.3 BRT model construction**

BRT methods have been successfully applied to research fields such as disease modeling [31]. The BRT method produces a series of trees, each of which grows on the remnant of the previous tree. Recent studies have shown that the BRT model can explain the interactions between exposures in observational studies [32]. In the BRT model, f(*x*) is an evaluation of the response *y* based on a vector predictor of *x*, which in turn is integrated as an additive form of b(*x*; *γ_m_*), as follows:

$$f(x)=\sum_{m} f_{m}(x)=\sum_{m} \beta_{m}b\left(x;\gamma_{m}\right)$$

where *β_m_* is the expansion factor and *b*(*x*; *γ_m_*) represent the individual trees with parameters *y* and variables *x*. The coefficient *β_m_* reflects the weights allocated to the nodes of each tree and identifies the type of combination predicted for each tree. In this approach, the three regularization parameters, number of trees, learning rate (lr) and tree complexity (tc), should be optimized. The complexity (tc) should be optimized [33]. To this purpose, in this study, various nt, tc (1-10), and lr (0.001, 0.05, and 0.01) are allocated to the training of the BRT model in order to maximize the model performance [34].

**2.4 DLNM model and GAM construction**

DLNM has been extensively used to assess the exposure-lag-response relationship between environmental factors and human diseases, such as congenital heart disease, HFRS, non-accidental deaths and so on [35, 36]. The model can be written as follows:

$$\log\left[E\left(Y_{t}\right)\right]=\alpha+NS(M,df,\mathrm{lag},df)+\Sigma NS\left(X_{i}\right)+NS(\text{ Time },df)+\beta DOW_{t}+\gamma\text{ Holiday }_{t}$$

To analyze the lag and extreme effects of environmental factors, Humidity, wind speed and SO_2_ were taken and applied to the cross-basis functions of DLNM. Here, *Y_t_* was the number of daily counts of HFRS cases in daily t; *α* was the intercept of the whole model; *NS* is a natural cubic spline that acts as a smooth function of the model; *M* represents the estimated environmental variable related to HFRS; *X_t_* is the other environmental variables in the pathogenesis of HFRS that requires nonlinear confounding effect adjustment; *NS* was used to adjust for daily confounding in the model; *DOW* is a categorical variable for day of week; Holiday is a binary variable used to control the effect of Chinese public holidays, *β* and *γ* are the regression coefficients; The optimal degrees of freedom (*df*) for the spline function were estimated by Akaike information criterion for quasi-Poisson (Q-AIC) and minimum partial regression coefficient (PACF_min_) criteria; NS of 4,6 and 8 *df* were used for wind speed, SO_2_ and relative humidity respectively, and the lag space was set to 3 *df*. NS with 5*df*/year was applied to time variable. In addition, as the incubation period for human hantavirus infection is typically 7-14 days, our model applied the Q-AIC guidelines using a delay of up to 16 days.

In our study, the median environmental variable has been used as a reference value to compute the relative risk. We then compared the 25th and 75th percentile of each environmental variable (“low” and “high”) to its median value, in order to explore the stratified effect of modification and qualitatively study the association among environmental factors and HFRS cases. The impact of environmental factors was analyzed by stratifying by gender, age group and number of diagnostic delayed days in order to identify susceptible populations and their corresponding sensitivities.

Subsequently, the GAM method was used to explore the interaction of meteorological and pollutant factors on the prevalence of HFRS. The model can be written as follows:

$$\log[E(Yt)]=\alpha2+s_{1}(X_{1},X_{2})+s_{2}(X_{3})+s_{3}(\text{ day })$$

*α*2 is the intercept; *X*_1_ indicates one of the environmental factors (humidity, wind speed and SO_2_) whereas *X*_2_ and *X*_3_ denote the other two; *s* () indicates penalized spline function. *s*_1_(*X*_1_, *X*_2_) is a spline function of the interaction between the parameters *X*_1_ and *X*_2_*.* (*X*_1_, *X*_2_, *X*_3_ are all 16 lagged variables.)

**2.5 Statistical analysis**

As there were missing values in the incidence data of HFRS in Shenyang, we performed linear interpolation to compensate as soon as possible in order to better apply Box-Jenkins and exponential smoothing methods for incidence prediction. A total of 10 sets of data were missing, located in the years 2011-2019, mainly in 2016 and 2019. Of these, only a single month was missing in 2011, 2012, 2014, 2015, and 2017, while two months were missing in 2016 (September-October) and three months were missing in 2019 (February, July, and October). For the influence of meteorological and pollutant factors on the number of HFRS cases, Spearman correlation analysis was used for feature selection, followed by BRT to fit the selected features to the variables and interaction tests. We developed a DLNM with a maximum lag of 16 days to evaluate the lagged, stratification and extreme effects of pollution and meteorological factors on the cases of HFRS. A GAM then was established to explore the interaction of SO_2_ and two other meteorological factors on HFRS cases.

All analyses in our study were performed in R software (version 4.1.3).

**3.Results**

**3.1 Descriptive characteristics of HFRS cases and environmental factors**

A total of 1,880 cases of HFRS were reported in Shenyang from 2005-2019, of which the incidence of HFRS was predominantly in young adults aged 20-50, accounting for 71.81% of all cases. Men are more susceptible than women at a ratio of 3.67:1 (1477:403). For 2005-2019, the incidence of HFRS in Shenyang was 25.03 per 100,000, and the case fatality rate was 0.691% (Table 1).

**Table 1. Distribution of the hemorrhagic fever with renal syndrome (HFRS) cases by age and season group in Shenyang, 2005-2019.**

| Characteristic | | 0-20 | 20-50 | ＞50 | Total | Population | Incidence | No of Deaths | Mortality | Case fatality |
| --- | --- | --- | --- | --- | --- | --- | --- | --- | --- | --- |
|  |  | No of HFRS cases (%) | | |  |  | （per 10^5^） |  | （per 10^5^） | （%） |
| Year | 2005 | 31(6.33 %) | 368(75.10%) | 91(18.57 %) | 490 | 6962186 | 7.04 | 4 | 0.057 | 0.816 |
|  | 2006 | 18(5.44 %) | 250(75.53%) | 63(19.03 %) | 331 | 7010640 | 4.72 | 3 | 0.043 | 0.906 |
|  | 2007 | 11(5.91 %) | 139(74.73 %) | 36(19.35 %) | 186 | 7066666 | 2.63 | 2 | 0.028 | 1.075 |
|  | 2008 | 5(2.78 %) | 139(77.22 %) | 36(20%) | 180 | 7116384 | 2.53 | 0 | 0.000 | 0.000 |
|  | 2009 | 6(4.65 %) | 88(68.22 %) | 35(27.13 %) | 129 | 7150272 | 1.80 | 1 | 0.014 | 0.775 |
|  | 2010 | 6(6.38 %) | 60(63.83 %) | 28(29.79%) | 94 | 7180769 | 1.31 | 0 | 0.000 | 0.000 |
|  | 2011 | 5(5.81 %) | 59(68.60 %) | 22(25.58 %) | 86 | 7211479 | 1.19 | 0 | 0.000 | 0.000 |
|  | 2012 | 3(3.70 %) | 59(72.84 %) | 19(23.46%) | 81 | 7237420 | 1.12 | 0 | 0.000 | 0.000 |
|  | 2013 | 1(1.37 %) | 49(67.12 %) | 23(31.51 %) | 73 | 7259528 | 1.01 | 0 | 0.000 | 0.000 |
|  | 2014 | 0(0%) | 28(54.90 %) | 23(45.10 %) | 51 | 7289761 | 0.70 | 0 | 0.000 | 0.000 |
|  | 2015 | 1(2%) | 35(70%) | 14(28%) | 50 | 7306224 | 0.68 | 0 | 0.000 | 0.000 |
|  | 2016 | 2(6.06 %) | 25(75.76%) | 6(18.18 %) | 33 | 7324009 | 0.45 | 1 | 0.014 | 3.030 |
|  | 2017 | 1(2.22 %) | 23(51.11 %) | 21(46.67 %) | 45 | 7356745 | 0.61 | 0 | 0.000 | 0.000 |
|  | 2018 | 3(8.33%) | 21(58.33 %) | 12(33.33%) | 36 | 7414719 | 0.49 | 2 | 0.027 | 5.556 |
|  | 2019 | 1(6.67 %) | 7(46.67 %) | 7(46.67 %) | 15 | 7511923 | 0.20 | 0 | 0.000 | 0.000 |
| Seasons | Spring(Mar-May) | 14(4.53 %) | 209(67.64 %) | 86(27.83 %) | 309 | - | - | 4 | - | 1.294 |
|  | Summer(Jun-Aug) | 33(4.51 %) | 543(74.18 %) | 156(21.31 %) | 732 | - | - | 3 | - | 0.410 |
|  | Autumn(Sep-Nov) | 24(7.08 %) | 223(65.78 %) | 92(27.14 %) | 339 | - | - | 2 | - | 0.590 |
|  | Winter(Dec-Feb) | 23(4.60%) | 375(75%) | 102(20.40%) | 500 | - | - | 4 | - | 0.800 |
| Total | | 94(5%) | 1350(71.81%) | 436(23.19%) | 1880 | 7511923 | 25.03 | 13 | 0.173 | 0.691 |

The onset of HFRS showed significant differences in seasonality, age, and delayed days in diagnosis of onset (*p*<0.05) (Table 2). From Table 2 meaningful subgroups differed in seasonal distribution, showing that different age groups were mainly concentrated in summer and winter, while groups with different days of delayed onset were mainly concentrated in spring and winter. Summary statistics of all HFRS cases and environmental variables in Shenyang are shown in Table S1. Figure 1 shows the time series distribution of daily cases of HFRS and environmental factors from 2005-2019. There are distinct seasonal variations in both HFRS and environmental conditions.

**Table 2. Stratified characteristics of the hemorrhagic fever with renal syndrome (HFRS) seasonal cases of Shenyang.**

| Characteristic | | Spring | Summer | Autumn | Winter | Total | *P*-value |
| --- | --- | --- | --- | --- | --- | --- | --- |
|  |  | No of HFRS cases (%) | | | |  |  |
| Age | 0-20 | 14(4.53 %) | 33(4.51 %) | 24(7.08 %) | 23(4.60%) | 94 | ＜0.05 |
|  | 20-50 | 209(67.64 %) | 543(74.18 %) | 223(65.78 %) | 375(75%) | 1350 |  |
|  | ＞50 | 86(27.83 %) | 156(21.31 %) | 92(27.14 %) | 102(20.40%) | 436 |  |
| The interval of days | 0-4 | 428(38.35%) | 219(19.62%) | 169(15.14%) | 300(26.88%) | 1116 | ＜0.05 |
|  | 5-9 | 248(42.18%) | 96(16.33%) | 94(15.99%) | 150(25.51%) | 588 |  |
|  | 10- | 56(31.82%) | 24(13.64%) | 46(26.14%) | 50(28.41%) | 176 |  |
| Occupation | Farmer | 210(33.49%) | 135(21.53%) | 109(17.38%) | 173(27.59%) | 627 | >0.05 |
|  | Home | 219(35.44%) | 116(18.77%) | 119(19.26%) | 164(26.54%) | 618 |  |
|  | Other | 303(47.72%) | 88(13.86%) | 81(12.76%) | 163(25.67%) | 635 |  |

**3.2 Time-series analysis of HFRS% of Holt-Winters and SARIMA model in monthly and seasonal prediction.**

From the factor decomposition diagrams in Figure S2, the annual incidence rate of HFRS is trending down, and after removing the trend effect from the original series, the difference in the average seasonal index among different quarters is the difference caused by the seasonal effect. HFRS in Shenyang peaked twice a year in the first and fourth quarter. The monthly HFRS has the characteristics of bimodal distribution, with the first peak in March-May, the second peak in November-December, and the seasonal inelasticity rising from April-August. The incidence of HFRS in Shenyang is characterized by a bimodal monthly distribution, with the first peak in March-May, the second peak in November-December, and an exponential decline in April-August, and a seasonal inverse rise starting in September.

The model fixed-order plots in Figures S3 allow the SARIMA seasonal and monthly models to be parameterized for the ACF, PACF plots, combined with the “auto.arima()” function to correct for the AR and MA parameters (parameter estimates < 2 times the sample standard deviation). The model parameters and tests are shown in Table 3, it gives the forecasting accuracy of two models for the HFRS series. The SARIMA model has lower values for RMSE, MAE and MAPE, which means the SARIMA is more accurate.

**Table 3. Performance measures of time series techniques for the hemorrhagic fever with renal syndrome (HFRS) incidence in Shenyang.**

| Model | Best parameters | Method | Box-Ljung test | | | RMSE | MAE | MAPE |
| --- | --- | --- | --- | --- | --- | --- | --- | --- |
|  |  |  | X-squared | Df | p-value |  |  |  |
| Holt-Winters seasonal model | α= 0.27, β=0.38 ,γ=1 | Additive | 0.087 | 2 | 0.957 | 0.198 | 0.13 | 40.215 |
| Holt-Winters monthly model | α= 0.24, β=0.05 , γ=0.75 | Additive | 7.386 | 6 | 0.287 | 0.078 | 0.054 | 71.535 |
| SARIMA seasonal model | ((1,2),1,0) (0,1,0) [4] | Additive | 6.59*10^-6^ | 1 | 0.998 | 0.182 | 0.116 | 38.031 |
| SARIMA monthly model | (1,1,0) (2,1,0) [12] | Multiple | 0.381 | 1 | 0.537 | 0.071 | 0.049 | 65.968 |

After building a suitable model based on the model parameters, the incidence of HFRS from 2005-2018 was used as the training set, and 2019 was used as the validation set to validate the model established by the training set. The Holt-Winters and SARIMA models were applied to predict the incidence of HFRS, respectively, and the prediction effects were plotted as obtained in Figure 2 and Table S2. From them the Holt-Winters model predicts trends closer to the actual values than the SARIMA model. the 95% confidence interval for the SARIMA model is narrower than the Holt-Winters model and its interval contains all the actual values. Figure S4-S5 show the tests of goodness of fitness and significance for the series of HFRS incidence from the two methods.

**3.3 Feature selection and fitting of environmental factors for HFRS**

Spearman correlation analysis showed that HFRS was significantly correlated with humidity (r=-0.10, p<0.01), wind speed (r=0.07, p<0.05) and SO_2_ (r=0.09, p<0.01) (Table S3). Furthermore, to fit the BRT model, we set the model parameters: tree complexing was 5, learning rate was 0.005, and bag. fraction was 0.5. According to Figure S6, the degree of fit of each of the three environmental factors fitting functions was like the Spearman correlation results, and the trend with the number of HFRS incidence was significant.

Depending on the distribution of observations in the environment space, the fitting function can give a distribution of fitted values relating to each predictor. The values at the top of each graph indicate the weighted average of the fitted values associated with each non-factor predictor. According to the interaction fitting function in Figure 3(A, B), environmental factors fit better at moderate levels of interaction.

**3.4 The lag relationship between environmental variables and the incidence of HFRS**

Figure S7 shows that the non-linear exposure–lag–response association among daily humidity, wind speed, SO_2_ and HFRS incidence cases, which indicated that these factors are relatively high risk above their median levels at different lag days. Different lag times correspond to different effects, specifically the effect of low wind speed occurs rapidly but lasts for a short time, the effect of high wind speed has a longer lag time but has a greater impact, while high humidity can have a transient effect on HFRS and high levels of SO_2_ can have a transient or continuous effect on HFRS, with the effect initially concentrated in the lag time of 0-5 days and after 15 days.

Estimates of the impact of meteorological and pollutant factors on HFRS cases show varied lagging features. Figure S8 shows the overall effect of environmental variables for total, gender, age and delayed-days HFRS cases within 16 days. Overall, these meteorological and pollutant variables were significantly relevant to HFRS cases. We found that RRs increased with the improvement of humidity, wind speed, and SO_2_, suggesting that higher humidity, wind speed, and SO_2_ increased the risk of HFRS. Yet humidity and SO_2_ separately reached the peak at 98% and 229.1μg/m^3^, then began to decrease or stabilize. Wind speed peaked at 3m/s and decreased. In general, similar trends in exposure-response relationships between environmental variables and cases of HFRS disease by gender, age and delayed-days group compared to total cases are shown in Figure S8. The minimum risk of incidence (RR_min_ values for environmental factors such as humidity, wind speed and SO_2_ were 16%, 8.1m/s and 223μg/m^3^ respectively.

Generally, analogous trends in exposure-response and lag-response relationships among environmental variables and HFRS cases across gender, age, and delay days of groups compared to total cases are shown in Figures S8, 4, and 5.

**3.5 Exposure-response relationships for environmental factors with different lag times**

As shown in Figure 4, the effects of humidity and SO_2_ on HFRS differed across lag times and stratification factors when the study lag time points were 0 and 16 days. The RR of the effect of humidity on HFRS cases tended to increase at lag 0 and 16 days, with humidity RR values reaching a maximum at 20-40% and above 90% at lag 0, while lag 16 days only showed a maximum RR value at high humidity. The effect values of wind speed and SO_2_ on HFRS cases at lags 0 and 16 days showed a trend of increasing and then decreasing RR, with RR values in the range of 2-4m/s and 200-250μg/m^3^, respectively. Within the different grouping intervals, the trend of RR effect values for humidity within Delay 0-4 days was slightly different from the overall, and the RR effect values for wind speed within Female, Delay 0-4, Delay 5-9 days, and SO_2_ within Delay 0-4 days were significantly different.

**3.6 Effects of extreme environmental variables on HFRS cases**

To determine the effect of extreme environmental factors on the HFRS, the estimated effects were examined by comparing the 25th or 75th percentile of relative humidity, wind speed and SO_2_ with their median values. Table 4 shows the cumulative impact of the lag factor extremes on the HFRS at 16 lag days. We found that extreme high levels of SO_2_ were positively linked to the onset of HFRS, while extreme low levels of SO_2_, with no wind effect, had a protective effect, and the RR values of cumulative effects of were 2.583 (1.145,5.827) for high SO_2_ effect and 0.577 (0.370, 0.898) for cold SO_2_ effect. At 16 lag days, significant cumulative effects of low windy conditions were observed in males (RR value: 0.490 (0.241,0.997)), in the over 50 years age group (RR value: 0.335 (0.113,0.992)) and in delayed onset for over 10 days (RR value: 0.324 (0.106,0.983)).In turn, women at extreme SO_2_ levels and patients with a delayed onset of 5-9 days are susceptible, with their RR values: 8.122 (1.009,65.403) for high SO_2_ and 0.285 (0.090,0.898) for low SO_2_, 4.491(1.246,16.193) for high SO_2_ and 0.427(0.213, 0.858) for low SO_2_, respectively.

**Table 4. The cumulative effects of extreme (25^th^ and 75^th^ percentile vs. median level) meteorological and air-pollution factors on HFRS cases of children by sex, age and delay.**

| Series | Variables | Cumulative effects(95%CI) | | | | | |
| --- | --- | --- | --- | --- | --- | --- | --- |
|  |  | Dry effect | Wet effect | Low windy effect | High windy effect | Low SO_2_ effect | High SO_2_ effect |
|  | Total cases | 0.761(0.158,3.673) | 0.557(0.129,2.405) | **0.487(0.260,0.912)** | 1.617(0.982,2.663) | **0.577(0.370,0.898)** | **2.583(1.145,5.827)** |
| Sex | Male | 0.461(0.078,2.737) | 0.462(0.088,2.428) | **0.490(0.241,0.997)** | 1.593(0.906,2.801) | 0.692(0.425,1.126) | 1.832(0.750,4.474) |
|  | Female | 9.817(0.203,473.684) | 4.091(0.131,127.899) | 0.683(0.153,3.039) | 1.075(0.347,3.334) | **0.285(0.090,0.898)** | **8.122(1.009,65.403)** |
| Age | 20-50 years | 0.745(0.094,5.870) | 0.535(0.083,3.451) | 0.523(0.225,1.213) | 1.544(0.787,3.032) | 0.645(0.363,1.146) | 2.031(0.706,5.845) |
|  | 50- years | 1.574(0.097,25.407) | 0.723(0.053,9.803) | **0.335(0.113,0.992)** | 2.007(0.855,4.711) | 0.530(0.240,1.167) | 3.287(0.767,14.079) |
| Delay | 0-4 days | 0.303(0.005,19.164) | 0.061(0.002,2.207) | 0.589(0.129,2.677) | 1.515(0.460,4.993) | 0.408(0.126,1.319) | 3.652(0.438,30.408) |
|  | 5-9 days | 0.897(0.088,9.191) | 1.540(0.175,13.525) | 0.451(0.180,1.133) | 1.874(0.884,3.971) | **0.427(0.213,0.858)** | **4.491(1.246,16.193)** |
|  | 10- days | 1.285(0.094,17.643) | 0.149(0.012,1.899) | **0.324(0.106,0.983)** | 1.392(0.596,3.254) | 0.638(0.314,1.297) | 2.104(0.570,7.762) |

Bold font indicates statistical significance at the 0.05 level.

The distributed lagged effects of extreme environmental factors at various lag days for all groups were showed in Figure 5. We found that the dry effect indicated a maximum RR value on the current day, peaking at 4 lag days and then showed a U-shaped curve along the lag days, and the RR value subsequently decreased for the next days and then turned to rise along the lag days, whereas wet effect showed the opposite trend. The curve of dry and wet effect was roughly similar among different stratified groups. On the low windy effect, the 2 lag days is a peak followed by a decline, while the opposite is true for the high windy effect. The overall patients reached their highest effect at extreme high or low levels of SO_2_, usually at a lag of 1 day, followed by a gradual downward trend. female, aged >1 years and delayed 5-9 days remain the most sensitive people.

**3.7 Environmental interaction during humidity, wind speed, SO_2_ and HFRS cases**

GAMs were built to show the interaction effect among humidity, wind speed and SO_2_ on HFRS incidence (Figure 6). The program on the top side of Figure 6(A, B) shows the interaction effect of wind speed and humidity on HFRS. The HFRS infection risk increased as daily wind speed and humidity decreased. The plot to the bottom of Figure 6(C, D) indicates the interaction effect of SO_2_ and humidity, HFRS tends to occur in higher SO_2_ and lower humidity environmental conditions.

**4.Discussion**

From the time series and seasonal decomposition of the incidence of HFRS in Shenyang, combined with studies in various regions of China [37], it can be seen that there is a clear seasonal trend in the incidence of HFRS in China, with a decreasing trend year upon year. The peak in case reporting differs between single and double peaks across regions, with the main peak occurring between March and May each year. This study also shows a second peak in November to December, which may occur for the following reasons: (1) The peak of population movement is about March each year, when the mobility of urban life becomes more complex and the fast pace of life and consumption leads to a gradual decline in the demand for health. (2) After November, the cooler temperatures in the city lead to larger crowds and a greater temperature difference between indoors and outdoors, allowing host animals to enter human life more closely and the disease to be more contagious.

Currently, many scholars have conducted research on predictive models for the onset of infectious diseases. The SARIMA model had been used to predicted the incidence of HFRS [13, 38]. It shows that SARIMA has the characteristics of being unconstrained by data type and high applicability, integrating factors such as trend, periodicity and random error, so that it can be used in prediction studies of infectious diseases with periodic morbidity characteristics. Pritthijit Nathet al.applied both the SARIMA model and the Holt-winters seasonal model for the prediction of airborne particulate matter in eastern India, the Holt - winters model was considered to be simple in principle and had a high predictive accuracy for diseases with a cyclical pattern of onset [39]. In this study we discussed the effect of the SARIMA model applied to the HFRS series and compared it with the Holt-Winters model. In terms of model mechanism, the SARIMA model is suitable for predicting series that are smooth and stable over time compared to the Holt - winters model, which is suitable for predicting models with a single trend of change. However, research on time series has limitations, as both methods in this study are extrapolated forecasts based on historical data, usually considering the characteristics of the series itself, and cannot predict sudden changes in the data due to changes in external factors. Moreover, the occurrence and prevalence of infectious diseases are influenced by multiple natural factors, climate and other social factors. Our study thus explored the lagging, interactive and stratified effects of meteorological and pollutant factors on the prevalence of HFRS in Shenyang.

Firstly, we need to select and fit the features using the spearman method combined with a BRT. The results were chosen from three environmental factors, wind speed, humidity and SO_2_, in order to achieve a precise study of the influence of environmental factors on HFRS. Subsequently, the DLNM method was applied to examine the exposure-lag-response relationship between the average daily cases of HFRS disease and environmental factors in Shenyang from 2014-2019. The results showed a non-linear lagged relationship among meteorological, pollutant factors and HFRS. Extremely high concentration levels of SO_2_ increased the risk of HFRS, while low windy and low concentration levels of SO_2_ were protective against HFRS from 0-16d. It was also found that the lagged effects of different climatic and pollutant factors were not identical. The different delay periods reflect the fact that the lagged effect of each environmental variable may be related to the spread of infection influenced by various factors, including the growth of the virus in the external environment, the inclination of people to go outside, and seasonal changes in rodent populations [21, 40]. We demonstrated that high concentrations of SO_2_ significantly influenced the spread of HFRS after 0-5 and 15 lag days. Extreme low windy were strongly associated with HFRS from lag 0 to a maximum lag 16 days, suggesting that the incidence of HFRS may be lagged by approximately 16 days at low windy. It could be similar to the study by Zhang [41] et al. on HFMD, mainly because low windy may inhibit the spread of hantavirus-containing particles [42]. The effect of humidity on HFRS cases tended to increase at days 0 and 16 when the lag time was 16 days, with the greatest effect at 20-40% at lag 0 days and at over 90% at lag 16 days. Higher humidity levels may indicate that humidity affects the survival of the rodent host, in addition to affecting the infection and stability of the virus in the in vitro environment [21]. Furthermore, in contrast to previous studies, our study included pollutants in the HFRS influencing factors and explored the non-linear lag between SO_2_ and HFRS. Our study found that elevated SO_2_ concentrations increased the risk of HFRS infection at levels in the range of 200-250 μg/m^3^ and were significant in women and in patients with a delayed onset of 5-9 days. There is still controversy about the effect of SO_2_ on HFRS, which may be related to regional, population differences and the proportion of pollutants in the air. However, regarding the effect of SO_2_ on other infectious diseases, there were different reports showing a significant protective effect of SO_2_ against influenza [43](RR=0.892, 95% CI: 0.840-0.948), which probably due to the higher outdoor pollutants, resulting in a population more dependent on the indoor environment and less exposed to the virus.

Stratified analysis showed that the effects of meteorological and pollutant factors varied by sex, age group, and number of days delayed onset. Men were more sensitive to extreme low windy than women, and women were more sensitive to extreme SO_2_ concentration levels than men. Similar results have been reported in several studies on other infectious diseases [43].Patients over 50 years were more significantly affected by extreme low windy and showed a protective effect, as compared to other age groups. But based on previous studies, wind speed effects were generally significantly associated with lower age [44]. This study showed that HFRS mainly affected people under 50 years of age at low windy, which might be attributed to underlying factors such as social factors, population distribution, etc. In terms of delay days, patients with a delay of 5-9 days were more sensitive to extreme SO_2_ concentrations and patients with a delay of over 10 days were more susceptible to extreme low wind speed. The delay between the onset of HFRS and the time of diagnosis led to a lagging effect of environmental factors reinforced by the length of time the patient spent in the environment after onset. Additionally, we did not take temperature, barometric pressure into account in our study of the correlation between environmental factors and HFRS. Some studies had shown that mean and extreme temperatures were negatively correlated with cases of HFRS [20]. This study did not discuss the relationship with HFRS cases in terms of temperature, barometric pressure, and rainfall factors, showing different findings of HFRS in Shenyang before 2011 and after 2014, suggesting possible spatial and temporal variability.

The results of the interaction analysis showed that higher SO_2_ and lower humidity environments were the dangerous environmental conditions for the occurrence of HFRS. It was demonstrated that NO_X_ and SO_2_ in the air showed strong seasonal variations and that their concentrations were closely related to meteorological factors such as wind speed, temperature and relative humidity. Air pollution may impact the frequency of HFRS cases by modifying viral infectivity and immunity in humans and rodents [45, 46].The combined effect of low windy and low humidity also affected the development of HFRS disease. Analysis of the geographical distribution of the country suggested that this result could be attributed to the region's location in a climatic zone [47].

Our research benefits cover: (1) the study period is long, and the study collected case and environmental data over many years. (2) For the time series analysis, we applied two different models for comparison and also split the data into monthly and seasonal data for accurate comparison and forecasting. (3) Our study applies advanced statistical methods, not only applying spearman to feature selection, but also applying the BRT method to fit the screened variables and their interactions, followed by DLNM and GAM to analyze the lagged, extreme and cumulative effects of environmental factors. Our findings can provide evidence and guidance on the lagged effects and interactions of environmental factors on HFRS. It is worth pointing out that there were some limitations to our study. Firstly, there are cases of HFRS in this study that have been diagnosed both clinically and through the laboratory, which may be subject to diagnostic bias and are under-reported. Moreover, due to the regional limitations of this study, other regions should be referred to with caution in studying the impact and prediction of HFRS disease, considering regional characteristics, and making changes in model selection, parameters and factor selection.

**5.Supporting information**

**S1 Table.** **Descriptive statistics for daily HFRS cases, meteorological and air-pollution factors in Shenyang, 2005–2019.**

**S2 Table.** **Comparison of the prediction results of the two models for 2019 in Shenyang.**

**S3 Table.** **Spearman correlation between daily HFRS cases and the affected variables in Shenyang.**

**S1 Figure.** **The geographical location of Shenyang City in China.** The map was created by ArcGIS 10.3 (Environmental Systems Research Institute; Redlands, CA, USA). The base map was acquired from the data center for geographic sciences and natural sources research, CAS (http://www.resdc.cn/data.aspx?DATAID=201).

**S2** **Figure.** **Decomposition of determinants in HFRS cases with seasonal and monthly model.**

**S3 Figure.** **Autocorrelation function (ACF) and partial ACF charts of seasonal (A, B) and monthly (C, D) HFRS incidence with SARIMA model.**

**S4 Figure.** **Tests of goodness of fit for the error series of HFRS incidence from the Holt-Winters method.** (a)Autocorrelation function (ACF) plot for the seasonal Holt-Winters residual series; (A)Autocorrelation function (ACF) plot for the monthly Holt-Winters residual series; (b) Partial autocorrelation function (PACF) plot for the seasonal Holt-Winters residual series; (B) Partial autocorrelation function (PACF) plot for the monthly Holt-Winters residual series; (c) Standardized residual seasonal Holt-Winters series; (C) Standardized residual monthly Holt-Winters series. These manifested its adequacy and suitability of this data-driven hybrid model for the data.

**S5 Figure. The significance of SARIMA seasonal(A) and monthly(B) model.**

**S6 Figure. Fitting function variables and distribution of fitted values created based on the boosted regression tree models.**

**S7 Figure. Relative risk of meteorological and air-pollution variables on HFRS incidence over 16 lag days, including relative humidity, wind speed and SO_2_.**

**S8 Figure. Effect of different meteorological and air-pollution variables on the incidence of HFRS at different days for total, gender (male, female), age (20-50 years, and 50-years) and delay groups (0-4 days, 5-9 days and 10-days) in Shenyang.**

**6.Author Contributions**

**Conceptualization:** Ye Chen, Weiming Hou, Jing Dong.

**Data curation:** Ye Chen, Weiming Hou.

**Formal analysis:** Ye Chen, Weiming Hou.

**Investigation:** Ye Chen.

**Methodology:** Ye Chen, Weiming Hou, Jing Dong.

**Software:** Ye Chen, Weiming Hou.

**Validation:** Ye Chen, Weiming Hou, Jing Dong.

**Visualization:** Ye Chen, Weiming Hou.

**Writing –** original draft: Ye Chen, Weiming Hou, Jing Dong.

**Writing –** review & editing: Ye Chen, Weiming Hou, Jing Dong.

**References**

1.	Avsic-Zupanc T, Saksida A, Korva M. Hantavirus infections. Clin Microbiol Infect. 2019;21S:e6-e16. Epub 2014/04/23. doi: 10.1111/1469-0691.12291. PubMed PMID: 24750436.

2.	Gut AK, Gut R, Pencuła M, Jarosz MJ. New cases of suspected HFRS (Hantavirus infection) in south-eastern Poland. Ann Agric Environ Med. 2013;20(3):544-8. Epub 2013/09/28. PubMed PMID: 24069862.

3.	Sun W, Liu X, Li W, Mao Z, Sun J, Lu L. Effects and interaction of meteorological factors on hemorrhagic fever with renal syndrome incidence in Huludao City, northeastern China, 2007-2018. PLoS Negl Trop Dis. 2021;15(3):e0009217. Epub 2021/03/26. doi: 10.1371/journal.pntd.0009217. PubMed PMID: 33764984; PubMed Central PMCID: PMCPMC7993601.

4.	Zhang Y-Z, Zou Y, Fu ZF, Plyusnin A. Hantavirus Infections in Humans and Animals, China. Emerging Infectious Diseases. 2010;16(8):1195-203. doi: 10.3201/eid1608.090470.

5.	Vapalahti O, Mustonen J, Lundkvist Å, Henttonen H, Plyusnin A, Vaheri A. Hantavirus Infections in Europe. The Lancet Infectious Diseases. 2003;3(10):653-61. doi: <https://doi.org/10.1016/S1473-3099(03)00774-6>.

6.	Heyman P, Vaheri A, Lundkvist Å, Avsic-Zupanc T. Hantavirus infections in Europe: from virus carriers to a major public-health problem. Expert Review of Anti-infective Therapy. 2009;7(2):205-17. doi: 10.1586/14787210.7.2.205.

7.	Schmaljohn C, Hjelle B. Hantaviruses: a global disease problem. Emerg Infect Dis. 1997;3(2):95-104. Epub 1997/04/01. doi: 10.3201/eid0302.970202. PubMed PMID: 9204290; PubMed Central PMCID: PMCPMC2627612.

8.	He J, Christakos G, Wu J, Cazelles B, Qian Q, Mu D, et al. Spatiotemporal variation of the association between climate dynamics and HFRS outbreaks in Eastern China during 2005-2016 and its geographic determinants. PLoS Negl Trop Dis. 2018;12(6):e0006554. Epub 2018/06/07. doi: 10.1371/journal.pntd.0006554. PubMed PMID: 29874263; PubMed Central PMCID: PMCPMC6005641.

9.	Chen HX, Qiu FX. Epidemiologic surveillance on the hemorrhagic fever with renal syndrome in China. Chin Med J (Engl). 1993;106(11):857-63. Epub 1993/11/01. PubMed PMID: 7908258.

10.	Liu Q, Liu X, Jiang B, Yang W. Forecasting incidence of hemorrhagic fever with renal syndrome in China using ARIMA model. BMC Infect Dis. 2011;11:218. Epub 2011/08/16. doi: 10.1186/1471-2334-11-218. PubMed PMID: 21838933; PubMed Central PMCID: PMCPMC3169483.

11.	Frazer J, Notin P, Dias M, Gomez A, Min JK, Brock K, et al. Disease variant prediction with deep generative models of evolutionary data. Nature. 2021;599(7883):91-5. Epub 2021/10/29. doi: 10.1038/s41586-021-04043-8. PubMed PMID: 34707284.

12.	Qiu H, Zhao H, Xiang H, Ou R, Yi J, Hu L, et al. Forecasting the incidence of mumps in Chongqing based on a SARIMA model. BMC Public Health. 2021;21(1):373. Epub 2021/02/19. doi: 10.1186/s12889-021-10383-x. PubMed PMID: 33596871; PubMed Central PMCID: PMCPMC7890879.

13.	Qi C, Zhang D, Zhu Y, Liu L, Li C, Wang Z, et al. SARFIMA model prediction for infectious diseases: application to hemorrhagic fever with renal syndrome and comparing with SARIMA. BMC Med Res Methodol. 2020;20(1):243. Epub 2020/10/01. doi: 10.1186/s12874-020-01130-8. PubMed PMID: 32993517; PubMed Central PMCID: PMCPMC7526348.

14.	Opatowski L, Baguelin M, Eggo RM. Influenza interaction with cocirculating pathogens and its impact on surveillance, pathogenesis, and epidemic profile: A key role for mathematical modelling. PLoS Pathog. 2018;14(2):e1006770. Epub 2018/02/16. doi: 10.1371/journal.ppat.1006770. PubMed PMID: 29447284; PubMed Central PMCID: PMCPMC5814058.

15.	He J, Wang Y, Wei X, Sun H, Xu Y, Yin W, et al. Spatial-temporal dynamics and time series prediction of HFRS in mainland China: A long-term retrospective study. J Med Virol. 2023;95(1):e28269. Epub 2022/11/03. doi: 10.1002/jmv.28269. PubMed PMID: 36320103.

16.	Xiao H, Huang R, Gao L-D, Huang C-R, Lin X-L, Li N, et al. Effects of Humidity Variation on the Hantavirus Infection and Hemorrhagic Fever with Renal Syndrome Occurrence in Subtropical China. The American Society of Tropical Medicine and Hygiene. 2016;94(2):420-7. doi: 10.4269/ajtmh.15-0486.

17.	Zou Y, Xiao QY, Dong X, Lv W, Zhang SP, Li MH, et al. Genetic analysis of hantaviruses carried by reed voles Microtus fortis in China. Virus Res. 2008;137(1):122-8. Epub 2008/07/23. doi: 10.1016/j.virusres.2008.06.012. PubMed PMID: 18644410.

18.	Zhang X, Chen HY, Zhu LY, Zeng LL, Wang F, Li QG, et al. Comparison of Hantaan and Seoul viral infections among patients with hemorrhagic fever with renal syndrome (HFRS) in Heilongjiang, China. Scand J Infect Dis. 2011;43(8):632-41. Epub 2011/03/25. doi: 10.3109/00365548.2011.566279. PubMed PMID: 21428852.

19.	Tian H, Yu P, Bjørnstad ON, Cazelles B, Yang J, Tan H, et al. Anthropogenically driven environmental changes shift the ecological dynamics of hemorrhagic fever with renal syndrome. PLoS Pathog. 2017;13(1):e1006198. Epub 2017/02/01. doi: 10.1371/journal.ppat.1006198. PubMed PMID: 28141833; PubMed Central PMCID: PMCPMC5302841.

20.	Liu X, Jiang B, Gu W, Liu Q. Temporal trend and climate factors of hemorrhagic fever with renal syndrome epidemic in Shenyang City, China. BMC Infect Dis. 2011;11:331. Epub 2011/12/03. doi: 10.1186/1471-2334-11-331. PubMed PMID: 22133347; PubMed Central PMCID: PMCPMC3247297.

21.	Zhang WY, Guo WD, Fang LQ, Li CP, Bi P, Glass GE, et al. Climate variability and hemorrhagic fever with renal syndrome transmission in Northeastern China. Environ Health Perspect. 2010;118(7):915-20. Epub 2010/02/10. doi: 10.1289/ehp.0901504. PubMed PMID: 20142167; PubMed Central PMCID: PMCPMC2920909.

22.	Roda Gracia J, Schumann B, Seidler A. Climate Variability and the Occurrence of Human Puumala Hantavirus Infections in Europe: A Systematic Review. Zoonoses Public Health. 2015;62(6):465-78. Epub 2015/01/06. doi: 10.1111/zph.12175. PubMed PMID: 25557350.

23.	Luo Y, Lv H, Yan H, Zhu C, Ai L, Li W, et al. Meteorological change and hemorrhagic fever with renal syndrome epidemic in China, 2004-2018. Sci Rep. 2022;12(1):20037. Epub 2022/11/23. doi: 10.1038/s41598-022-23945-9. PubMed PMID: 36414682; PubMed Central PMCID: PMCPMC9681842.

24.	Lv CL, Tian Y, Qiu Y, Xu Q, Chen JJ, Jiang BG, et al. Dual seasonal pattern for hemorrhagic fever with renal syndrome and its potential determinants in China. Sci Total Environ. 2023;859(Pt 2):160339. Epub 2022/11/26. doi: 10.1016/j.scitotenv.2022.160339. PubMed PMID: 36427712.

25.	Dai MY, Zhang CQ. Analysis on epidemiological characteristics of scarlet fever,Shenyang city,2013-2017. Preventive Medicine Tribune. 2018.

26.	Zhang WY, Wang LY, Liu YX, Yin WW, Hu WB, Magalhaes RJ, et al. Spatiotemporal transmission dynamics of hemorrhagic fever with renal syndrome in China, 2005-2012. PLoS Negl Trop Dis. 2014;8(11):e3344. Epub 2014/11/21. doi: 10.1371/journal.pntd.0003344. PubMed PMID: 25412324; PubMed Central PMCID: PMCPMC4239011.

27.	Liu G, Sun B, Yu L, Chen J, Han B, Liu B, et al. Short-term exposure to ambient air pollution and daily atherosclerotic heart disease mortality in a cool climate. Environ Sci Pollut Res Int. 2019;26(23):23603-14. Epub 2019/06/17. doi: 10.1007/s11356-019-05565-5. PubMed PMID: 31203548.

28.	Mao Q, Zhang K, Yan W, Cheng C. Forecasting the incidence of tuberculosis in China using the seasonal auto-regressive integrated moving average (SARIMA) model. J Infect Public Health. 2018;11(5):707-12. Epub 2018/05/08. doi: 10.1016/j.jiph.2018.04.009. PubMed PMID: 29730253; PubMed Central PMCID: PMCPMC7102794.

29.	Zhu Y, Zhao Y, Zhang J, Geng N, Huang D. Spring onion seed demand forecasting using a hybrid Holt-Winters and support vector machine model. PLoS One. 2019;14(7):e0219889-e. doi: 10.1371/journal.pone.0219889. PubMed PMID: 31344050.

30.	Harrison E, Uchendu B, Victor-Edema U. Arima Fit to Nigerian Unemployment Data. 2012.

31.	Mazaheri H, Ghaedi M, Ahmadi Azqhandi MH, Asfaram A. Application of machine/statistical learning, artificial intelligence and statistical experimental design for the modeling and optimization of methylene blue and Cd(ii) removal from a binary aqueous solution by natural walnut carbon. Phys Chem Chem Phys. 2017;19(18):11299-317. Epub 2017/04/19. doi: 10.1039/c6cp08437k. PubMed PMID: 28418055.

32.	Orsini N, Moore A, Wolk A. Interaction Analysis Based on Shapley Values and Extreme Gradient Boosting: A Realistic Simulation and Application to a Large Epidemiological Prospective Study. Front Nutr. 2022;9:871768. Epub 2022/08/05. doi: 10.3389/fnut.2022.871768. PubMed PMID: 35923201; PubMed Central PMCID: PMCPMC9340268.

33.	De'ath G. Boosted trees for ecological modeling and prediction. Ecology. 2007;88(1):243-51. Epub 2007/05/11. doi: 10.1890/0012-9658(2007)88[243:btfema]2.0.co;2. PubMed PMID: 17489472.

34.	Foroughi M, Ahmadi Azqhandi MH, Kakhki S. Bio-inspired, high, and fast adsorption of tetracycline from aqueous media using Fe(3)O(4)-g-CN@PEI-β-CD nanocomposite: Modeling by response surface methodology (RSM), boosted regression tree (BRT), and general regression neural network (GRNN). J Hazard Mater. 2020;388:121769. Epub 2019/12/19. doi: 10.1016/j.jhazmat.2019.121769. PubMed PMID: 31848088.

35.	Zhang Q, Sun S, Sui X, Ding L, Yang M, Li C, et al. Associations between weekly air pollution exposure and congenital heart disease. Sci Total Environ. 2021;757:143821. Epub 2020/11/30. doi: 10.1016/j.scitotenv.2020.143821. PubMed PMID: 33248761.

36.	Cao R, Wang Y, Huang J, He J, Ponsawansong P, Jin J, et al. The Mortality Effect of Apparent Temperature: A Multi-City Study in Asia. Int J Environ Res Public Health. 2021;18(9). Epub 2021/05/01. doi: 10.3390/ijerph18094675. PubMed PMID: 33924779; PubMed Central PMCID: PMCPMC8124769.

37.	Wu G, Xia Z, Wang F, Wu J, Cheng D, Chen X, et al. Investigation on risk factors of haemorrhagic fever with renal syndrome (HFRS) in Xuancheng City in Anhui Province, Mainland China. Epidemiol Infect. 2020;148:e248. Epub 2020/10/03. doi: 10.1017/s0950268820002344. PubMed PMID: 33004084; PubMed Central PMCID: PMCPMC7592102.

38.	Sun L, Zou LX. Spatiotemporal analysis and forecasting model of hemorrhagic fever with renal syndrome in mainland China. Epidemiol Infect. 2018;146(13):1680-8. Epub 2018/08/07. doi: 10.1017/s0950268818002030. PubMed PMID: 30078384.

39.	Nath P, Saha P, Middya AI, Roy S. Long-term time-series pollution forecast using statistical and deep learning methods. Neural Comput Appl. 2021;33(19):12551-70. Epub 2021/04/13. doi: 10.1007/s00521-021-05901-2. PubMed PMID: 33840911; PubMed Central PMCID: PMCPMC8019307.

40.	Zhao Q, Yang X, Liu H, Hu Y, He M, Huang B, et al. Effects of climate factors on hemorrhagic fever with renal syndrome in Changchun, 2013 to 2017. Medicine (Baltimore). 2019;98(9):e14640. Epub 2019/03/01. doi: 10.1097/md.0000000000014640. PubMed PMID: 30817583; PubMed Central PMCID: PMCPMC6831229.

41.	Zhang Z, Xie X, Chen X, Li Y, Lu Y, Mei S, et al. Short-term effects of meteorological factors on hand, foot and mouth disease among children in Shenzhen, China: Non-linearity, threshold and interaction. Sci Total Environ. 2016;539:576-82. Epub 2015/09/20. doi: 10.1016/j.scitotenv.2015.09.027. PubMed PMID: 26386448.

42.	Lighthart B, Mohr AJ. Estimating downwind concentrations of viable airborne microorganisms in dynamic atmospheric conditions. Appl Environ Microbiol. 1987;53(7):1580-3. Epub 1987/07/01. doi: 10.1128/aem.53.7.1580-1583.1987. PubMed PMID: 3662508; PubMed Central PMCID: PMCPMC203913.

43.	Li X, Xu J, Wang W, Liang JJ, Deng ZH, Du J, et al. Air pollutants and outpatient visits for influenza-like illness in Beijing, China. PeerJ. 2021;9:e11397. Epub 2021/06/19. doi: 10.7717/peerj.11397. PubMed PMID: 34141466; PubMed Central PMCID: PMCPMC8179240.

44.	Yu G, Li Y, Cai J, Yu D, Tang J, Zhai W, et al. Short-term effects of meteorological factors and air pollution on childhood hand-foot-mouth disease in Guilin, China. Sci Total Environ. 2019;646:460-70. Epub 2018/07/30. doi: 10.1016/j.scitotenv.2018.07.329. PubMed PMID: 30056233.

45.	Ciencewicki J, Jaspers I. Air pollution and respiratory viral infection. Inhal Toxicol. 2007;19(14):1135-46. Epub 2007/11/08. doi: 10.1080/08958370701665434. PubMed PMID: 17987465.

46.	Weber TP, Stilianakis NI. Inactivation of influenza A viruses in the environment and modes of transmission: a critical review. J Infect. 2008;57(5):361-73. Epub 2008/10/14. doi: 10.1016/j.jinf.2008.08.013. PubMed PMID: 18848358; PubMed Central PMCID: PMCPMC7112701.

47.	Xiao H, Huang R, Gao LD, Huang CR, Lin XL, Li N, et al. Effects of Humidity Variation on the Hantavirus Infection and Hemorrhagic Fever with Renal Syndrome Occurrence in Subtropical China. Am J Trop Med Hyg. 2016;94(2):420-7. Epub 2015/12/30. doi: 10.4269/ajtmh.15-0486. PubMed PMID: 26711521; PubMed Central PMCID: PMCPMC4751944.

**Figures legend list**

**Figure 1** The time series distribution of daily HFRS cases, meteorological and air-pollution factors in Shenyang from 2005 – 2019.

**Figure 2** Resulting comparisons of the HFRS seasonal and monthly incidences using the preferred two models. The deep shaded regions indicate 80% confidence intervals, the light shaded regions indicate 95% confidence intervals.

**Figure 3** The fitting interactions of the association among humidity, windspeed,

SO_2_ and HFRS cases in Shenyang, 2014–2019 based on the boosted regression tree models.

**Figure 4** Summary of slices lag1-16 days exposure-response relationship between meteorological factors, air-pollution and HFRS cases for total, gender (male, female), age (20-50years, and 50-years) and delay groups (0-4days, 5-9days and 10-days) in Shenyang.

**Figure 5** Summary of estimated extreme effects at the 25th and the 75th percentile of relative humidity, wind speed and SO_2_ on HFRS cases for total, gender (male, female), age (20-50years, and 50-years) and delay groups (0-4days, 5-9days and 10-days) at different lag days. The median value of each meteorological factor (relative humidity: 61%, wind speed: 2 m/s, SO_2_: 26g/m^3^) is used as a reference level.

**Figure 6** The effect interactions of the association among humidity, windspeed, SO_2_ and HFRS cases in Shenyang, 2014–2019 based on the generalized additive models.
